# Supplementary material for: Structure-based evolution of a promiscuous inhibitor to a selective stabilizer of protein–protein interactions
Source: Nat Commun. 2020 Aug 7;11:3954. doi: 10.1038/s41467-020-17741-0 (PMC7414219; doi:10.1038/s41467-020-17741-0)
Supplement: Supplementary file 2 — Supplementary Information [file 41467_2020_17741_MOESM2_ESM.pdf]

SUPPLEMENTARY INFORMATION

Structure-based evolution of a promiscuous inhibitor to a selective stabilizer of protein-protein interaction stabilizers

Sijbesma et al.

Table of Contents

Supplementary Tables …………………………………………………………………………….... S2-S4

Tables 1 - 4: Chemical structures and docking scores of compounds A1-A6, B1-B4, C1-C3 and C3.1-C3.8

Table 5: Representative 14-3-3 client-derived (phospho-)peptide motifs for ligand selectivity analyses

Supplementary Figures ……………………………………………………………………………… S5-S24

Figure 1 - 3: Docking poses of AMP and selected Molport compounds

Figure 4: Fluorescence anisotropy titration data for AMP and selected Molport compounds

Figure 5: ITC data for stabilization of the 14-3-3β/ChREBP interaction by AMP

Figure 6: Observed polar contacts for the ChREBP-α2 peptide in the 14-3-3/ChREBP/**3** co-crystal structure

Figure 7: Crystallographic overlays of docking poses and co-crystal structures of 14-3-3/ChREBP/**3** and AMP

Figure 8: Structure-based comparisons of inhibitors and **3** stabilizer with common scaffold

Figure 9: Structure-based observations for optimization of the **3** interactions with 14-3-3 and ChREBP

Figure 10: Fluorescence anisotropy data for ligand titrations (SAR series)

Figure 11: Fluorescence anisotropy data for titrations series while varying concentrations of peptide and protein

Figure 12: Binding affinity and binding mode of representative 14-3-3 client motifs

Figure 13: Fluorescence anisotropy controls (FC-A and DMSO) for compound titrations to various client peptides

Figure 14: Protein characterization by ultra-high resolution liquid chromatography (UPLC)

Figure 15: Analytical LC-MS of the purified a2-peptides (acetylated and FITC-labeled)

Figure 16: Part I. Protein crystallization for the protein/peptide/compound complex of 14-3-3σ ΔC / ChREBP-α2 / **3**

Figure 17: Part II. Protein crystallization for the protein/peptide/compound complex of 14-3-3σ ΔC / ChREBP-α2 / **3**

Figure 18: Portion of the electron density maps for 6YGJ and 6YE9

Figure 19: Workflow in KNIME to filter the Molport library to prepare a subset for virtual screening

Figure 20: KNIME workflow to extract docking results

Figure 21: Analytical LC/MS of AMP

Figure 22: Analytical LC/MS of selected compounds from docking, in class A (A1 – A7)

Figure 23: Analytical LC-MS of selected compounds from docking, in class B (B1, B2, B3(=compound **1**))

Figure 24: Analytical LC-MS of selected compounds from docking, in class C (C1, C2, C3(=compound **2**))

Figure 25: Analytical LC-MS of SAR-by-catalog derivatives of C3 (**2**): (compounds **3** - **10**)

Supplementary Methods ………………………………………………………………………..... S25-S42

Virtual Screening and Molecular Docking Procedures

Organic Synthesis and Characterization

Supplementary References ………………………………………………………………………. S43

Supplementary Tables

Supplementary Table 1. Structure, docking and flexible docking scores for the AMP-like phosphates (class A)

ID	Structure	Docking score	IFD score	ID	Structure	Docking score	IFD score
**AMP**	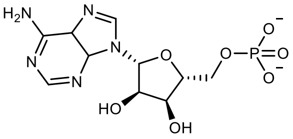	-12.06	-13.06	**A4**	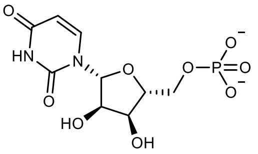	-12.74	-13.35
**A1**	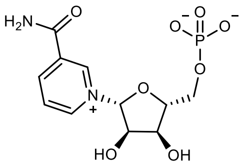	-13.64	-13.31	**A5**	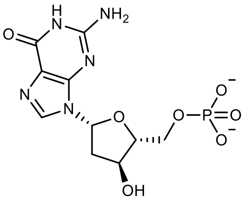	-12.72	-9.72
**A2**	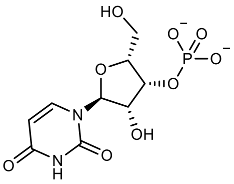	-13.26	-13.28	**A6**	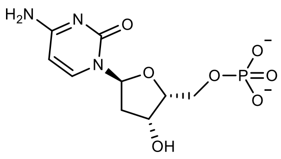	-12.55	-11.87
**A3**	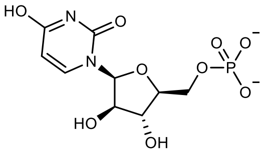	-13.09	-14.39	**A7**	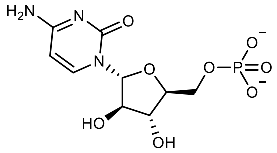	-12.48	-12.08

Supplementary Table 2. Structure, docking and flexible docking scores for the non-AMP-like phosphates (class B)

ID	Structure	Docking score	IFD score	ID	Structure	Docking score	IFD score
**B1**	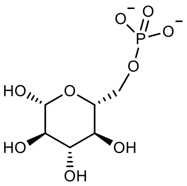	-12.19	-11.28	**B3 (1)**	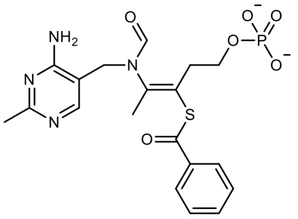	-12.12	-13.31
**B2**	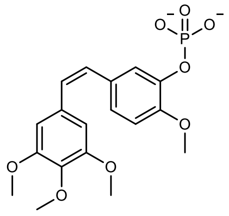	-11.76	-12.81				

Supplementary Table 3.Structure, docking and flexible docking scores for the non-AMP-phosphonates (class C)

ID	Structure	Docking score	IFD score	ID	Structure	Docking score	IFD score
**C1**	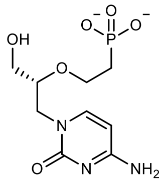	-10.50	-10.33	**C3** (**2**)	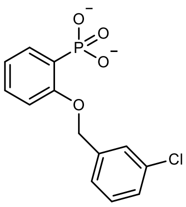	-10.17	-9.73
**C2**	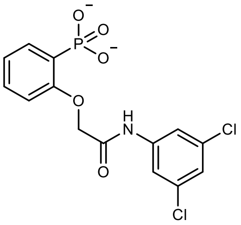	-9.81	-10.59				

Supplementary Table 4. Structure, docking and flexible docking scores for derivatives of **2** (analog-by-catalog SAR study)

ID	Structure	Docking score	IFD score	ID	Structure	Docking score	IFD score
**3**	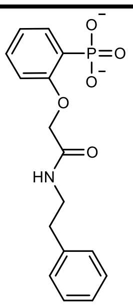	-9.26	-9.44	**7**	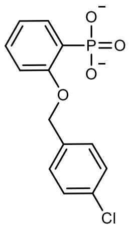	-9.09	-8.47
**4**	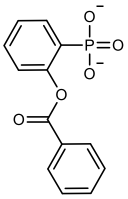	-8.83	-8.92	**8**	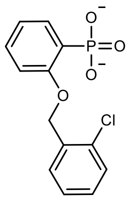	-9.04	-8.61
**5**	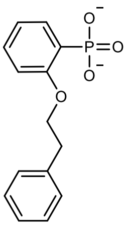	-8.69	-5.50	**9**	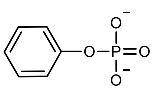	-9.05	-8.80
**6**	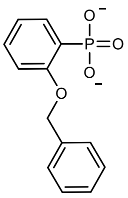	-8.89	-8.03	**10**	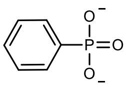	-8.44	-7.67

Supplementary Table 5. Representative 14-3-3 client-derived (phospho-)peptide motifs to assess selectivity of 14-3-3/ChREBP phosphonate hits

Client protein	14-3-3 binding mode	Phospho -site	Peptide sequence(protein residue #)	Peptide C-terminus	Reference PDB entries
**ChREBP**	phospho-independent (α-helix)	-	^{117}RDKIRLNNAIWRAWYIQYVKRRKSPV^{142}	*-CONH₂*	4GNT, 5F74
**ExoS**	phospho-independent (partial α-helical)	-	^{416}SGHGQGLLDALDLAS^{430}	*-CONH₂*	2O02
**TAZ**	Mode-I/II: xx**{pS/T}**x**P**xxxx	pS89	^{86}RSH{pSER}SPASLQLGT^{98}	*-CONH₂*	5N75
**ERα**	Mode-III: xx**{pS/pT}**x-*COOH* (C-terminal F-domain NR)	pT594	^{488}AEGFPA{pTHR}V^{595}	*-COOH*	4JDD, 4JC3
**p53**	Special type C-terminus: FK**{pT}EGP**DSD-*COOH*	pT387	^{362}SRAHSSHLKSKKGQSTSRHKKLMFK {pTHR}EGPDSD^{393}	*-COOH*	3LW1, 5MHC

Supplementary Figures

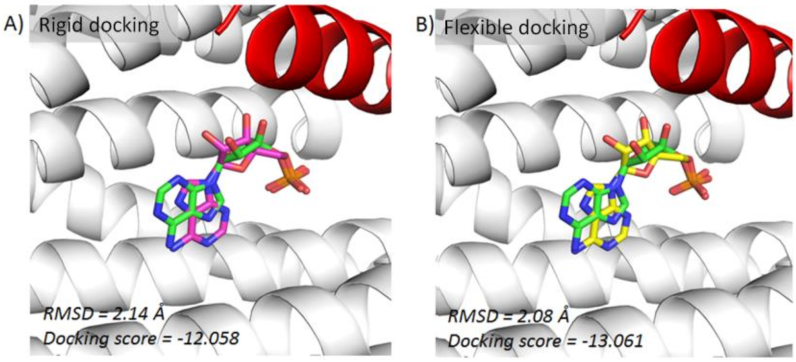

Supplementary Figure 1. Overlays of the AMP conformations in X-ray crystal structure (green sticks) and from A) rigid docking (magenta sticks) and B) induced fit (flexible) docking (IFD) (yellow sticks).

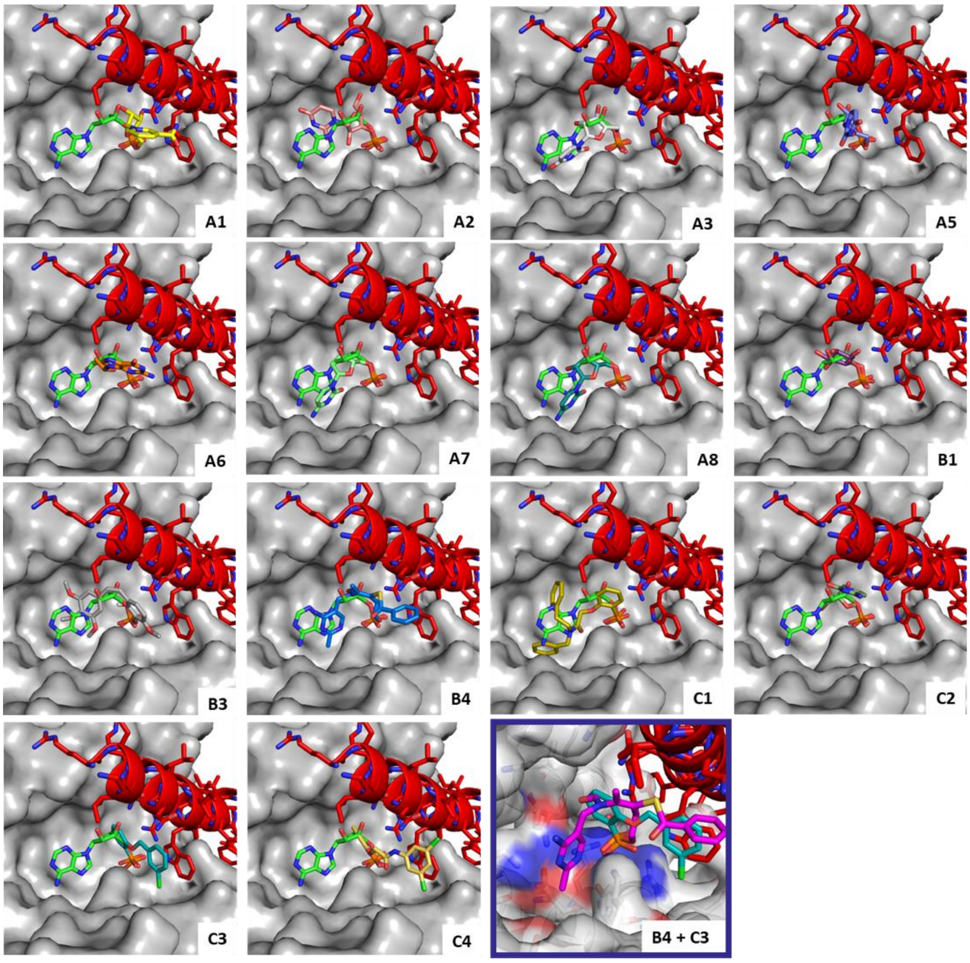

Supplementary Figure 2. Docking poses of AMP-like phosphates (class A); non-AMP-like phosphates (class B); and non-AMP-like phosphonates (class C) (colored sticks) overlay with AMP conformation in X-ray crystal structure (green sticks).

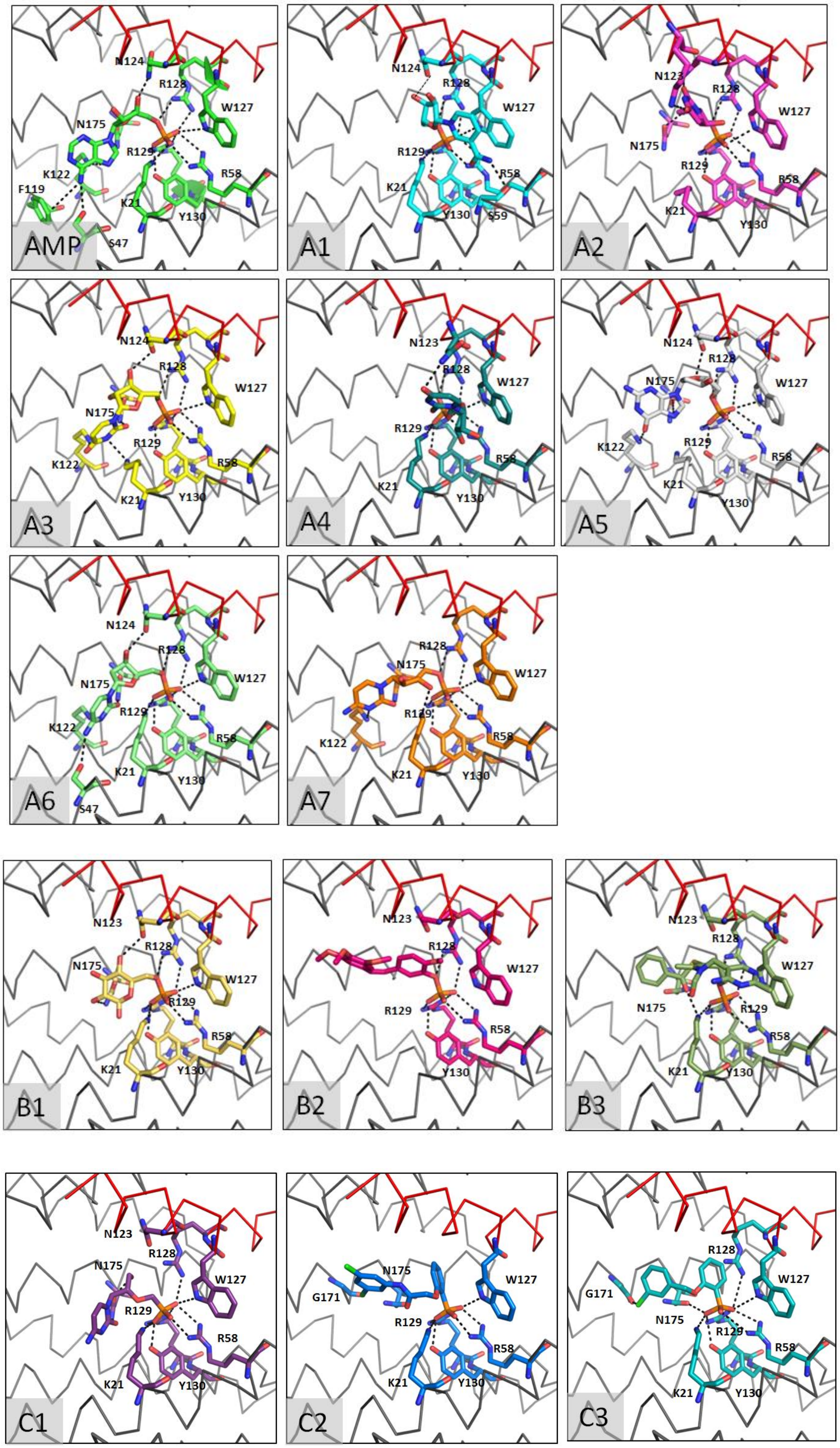

Supplementary Figure 3. Induced fit docking (IFD) poses of AMP-like phosphates (class A); non-AMP like phosphates (class B); and non-AMP-like phosphonates (class C) in ChREBP/14-3-3 pocket (PDB 5F74). The backbone of 14-3-3 (gray) and ChREBP (red) are depicted as ribbons. Amino acid side chains that undergo conformational changes during docking (sticks) are colored identical to the ligand.

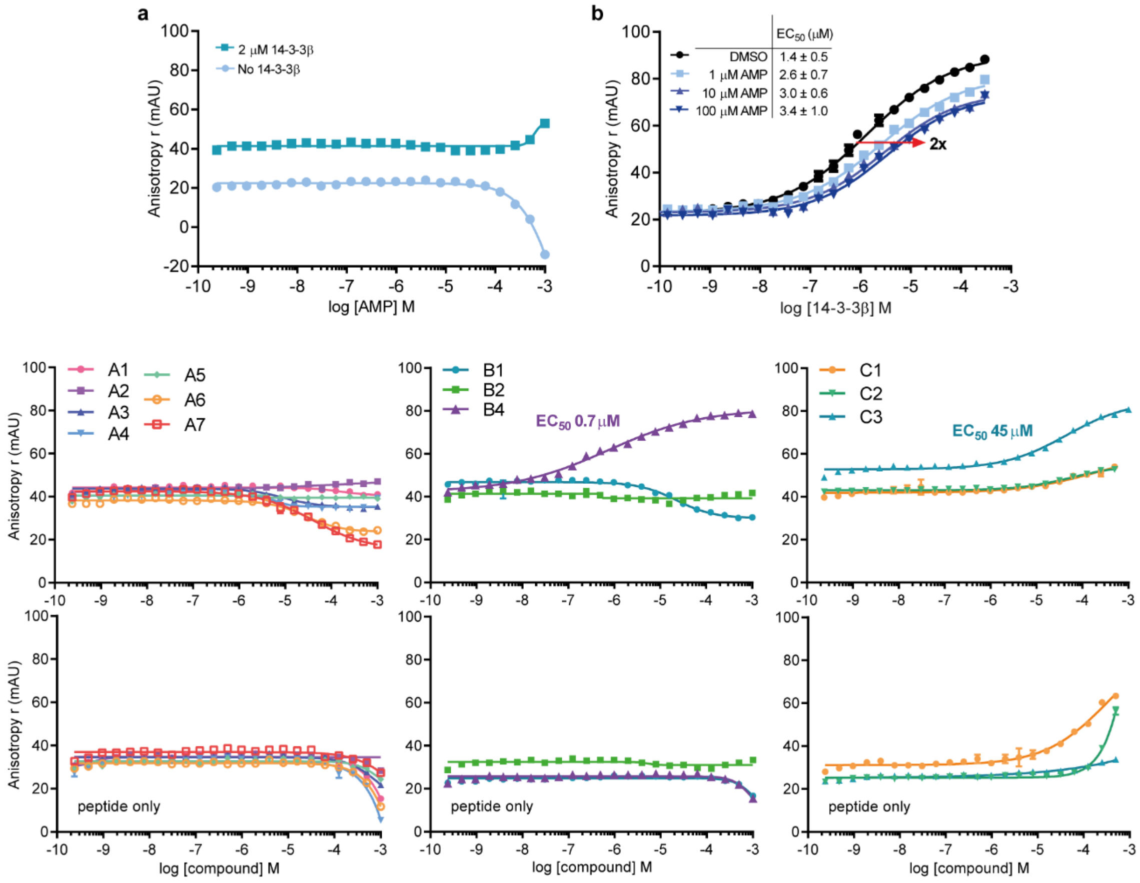

Supplementary Figure 4. Fluorescence anisotropy data for modulation of the ChREBP/14-3-3β interaction by AMP and Molport library hit compounds. a) Fluorescein isothiocyanate (FITC)-labeled ChREBP α2-peptide (residues 117-142; at 100 nM) titrated with AMP in absence or presence of 2 μM 14-3-3β. b) Dose-response curves for ChREBP α2-peptide titrated with 14-3-3β (starting from 300 μM), in the presence of increasing AMP concentrations (1, 10, 100 μM). c) FITC-labeled ChREBP α2-peptide and 14-3-3β (2 μM) titrated with 13 hits selected from the virtual screen. All samples contain 1% DMSO. Data points and error bars represent mean ± SEM, n=3 replicates. Source data are provided as a Source Data file.

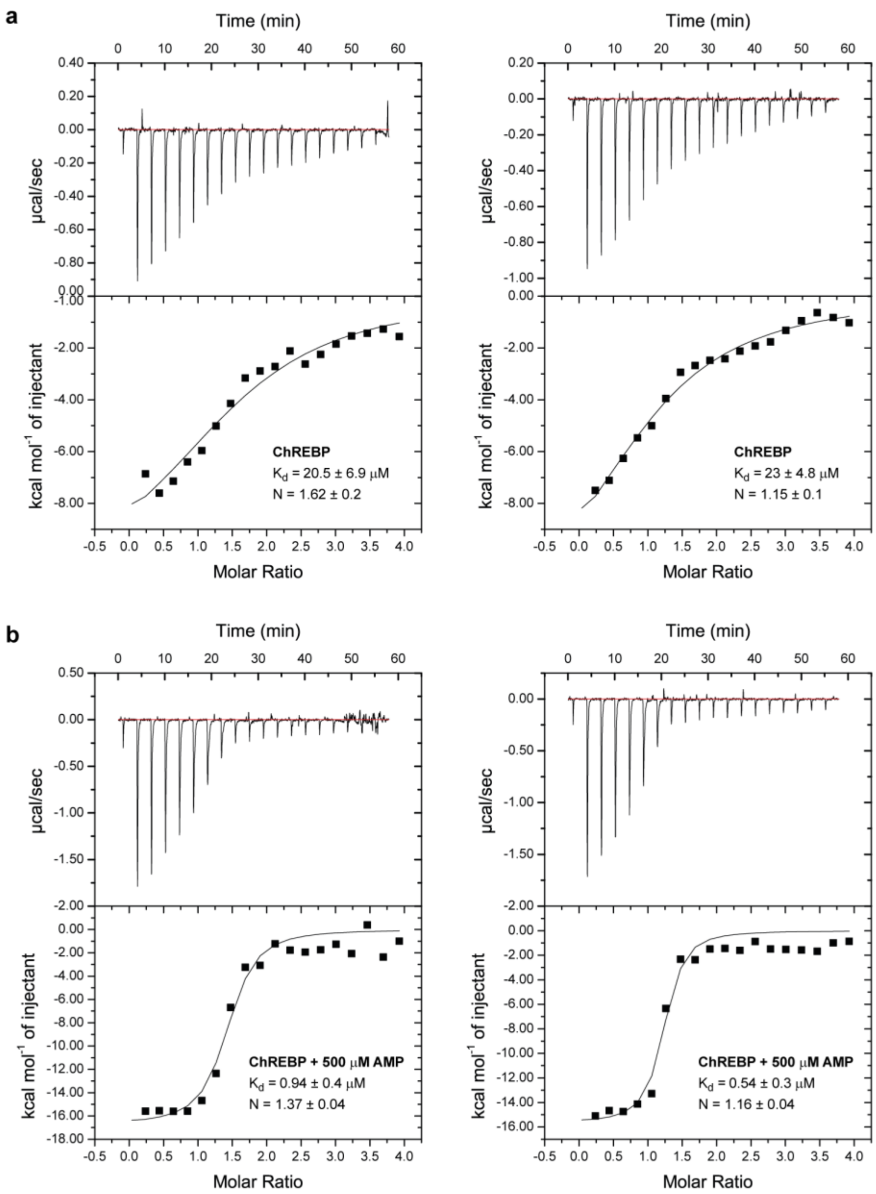

Supplementary Figure 5. Stabilization of the 14-3-3β/ChREBP interaction by AMP. Isothermal titration calorimetry (ITC) replicate data for ChREBP α2-peptide (600 μM) titrated into 14-3-3β (30 μM) in absence (a); or presence of AMP (500 μM) (b).

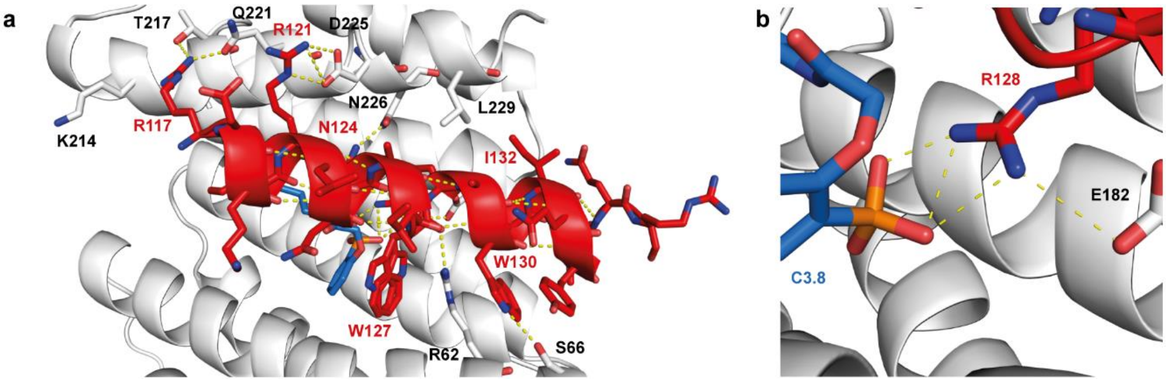

Supplementary Figure 6. Detailed polar contacts (dashed yellow lines) for the ChREBP-α2 peptide in the 14-3-3, ChREBP and **C3.8** co-crystal structure. a) Polar contacts were observed between R117 of ChREBP and 14-3-3's T217, Q221, and the backbone of K214; between R121 of ChREBP and D225 of 14-3-3; N124 of ChREBP and N226 of 14-3-3; the backbone of W127 of ChREBP and R62 of 14-3-3; W130 of ChREBP and S66 of 14-3-3; and Y131 of ChREBP with the backbone of E182 of 14-3-3 and an extensive network of internal polar contacts in ChREBP's backbone maintains its α-helical fold. b) Detailed view of the polar contact between R128 of ChREBP, the phosphonate of **C3.8** (**3**) and E182 of 14-3-3.

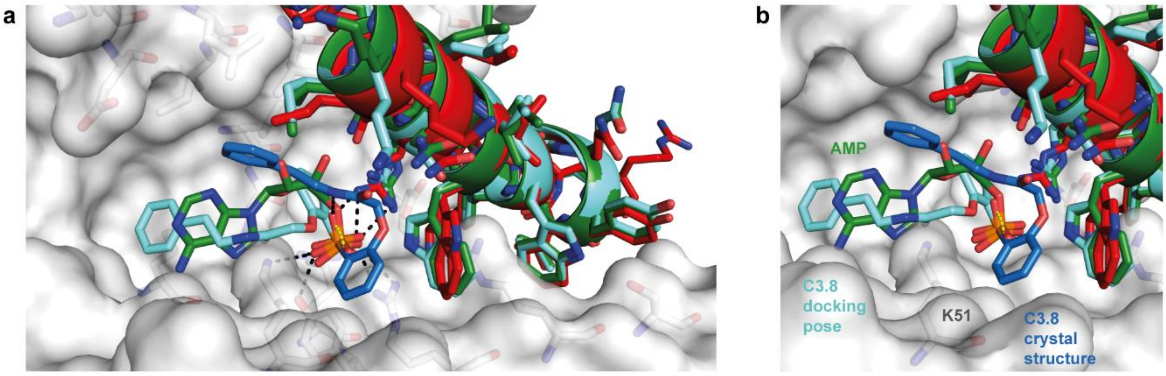

Supplementary Figure 7. Crystallographic overlays of the co-crystal structure of 14-3-3/ChREBP and AMP (PDB 5F74; green sticks and cartoon representation) or **C3.8** (**3**) (blue sticks for the compound, red sticks and cartoon for ChREBP peptide), and the predicted docking pose for **C3.8** (aquamarine sticks and cartoon) showing the entire 14-3-3 binding groove (a) or a zoom-in of the phospho-accepting pocket (b). Note that 14-3-3 (white semi-transparent surface) depicted here is the conformation it has in the co-crystal structure with ChREBP and **C3.8**, with the main difference the orientation of the K51 side chain (sticks representation). Whereas the side chain of 14-3-3's K51 remains highly flexible in many 14-3-3 co-crystal structures, (observed from weak electron density for the atoms, often resulting in multiple conformations), for the ChREBP/**C3.8** co-crystal structure it has a different orientation and interacts with a phosphonate oxygen which was not observed in both reported co-crystal structures of 14-3-3 and ChREBP, PDB 5F74 (with AMP) and 4GNT (with a free sulphate ion in the pocket; structure not shown here).

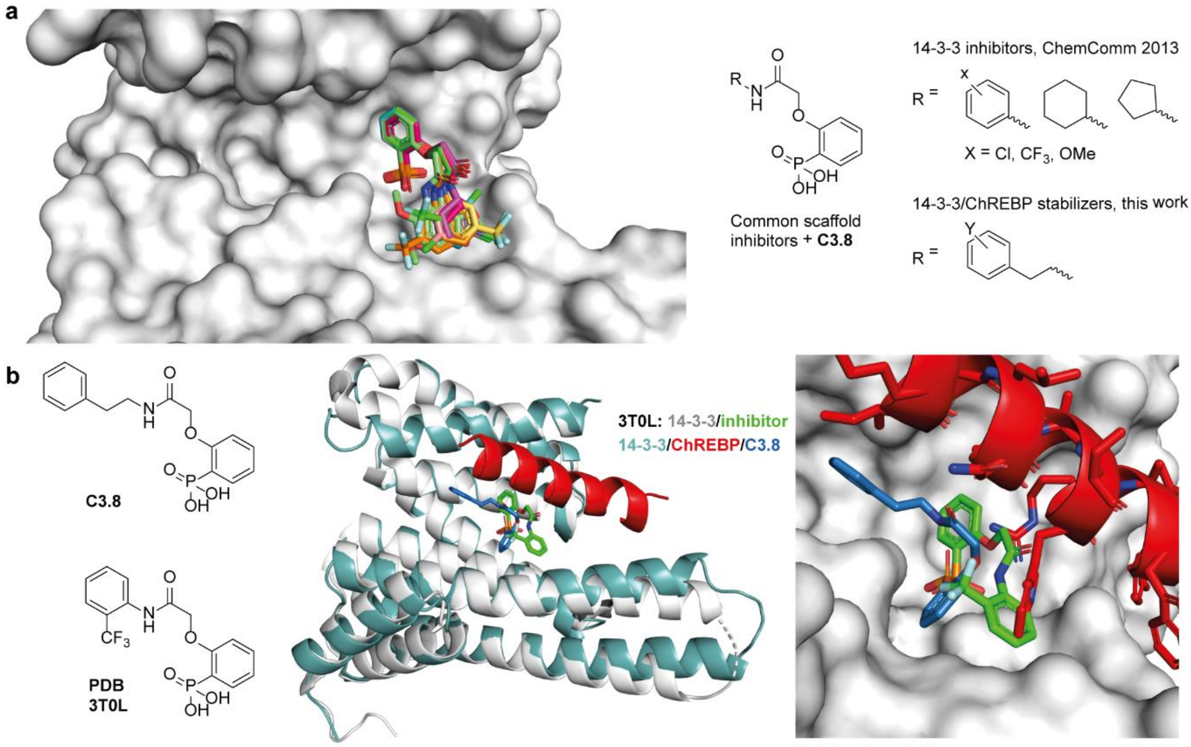

Supplementary Figure 8. Structure-based comparisons of the chemical structures and mode of action of 14-3-3 phosphonate inhibitors reported previously[1] and the 14-3-3/ChREBP stabilizer **C3.8** (**3**) reported in this work. a) Overlay of the eleven reported structures for phenylphosphonate-based inhibitors, all occupying the same shallow pocket in the 14-3-3 central groove. Chemical structure illustrates the common scaffold. b) Chemical structures and crystallographic overlays of **C3.8** (blue sticks) with 14-3-3/ChREBP (teal and red cartoon representation, respectively); and exemplary inhibitor (green sticks) with 14-3-3 (white cartoon) PDB entry 3T0L). The close up view illustrates clashes between the inhibitor and side chains of W127 and R128 (red sticks).

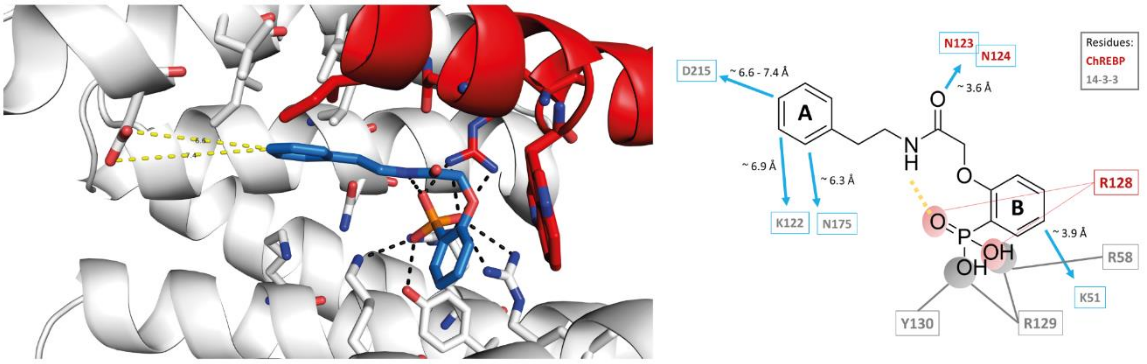

Supplementary Figure 9. Structure-based observations for optimization of the **C3.8** (**3**) interactions with 14-3-3 and ChREBP.

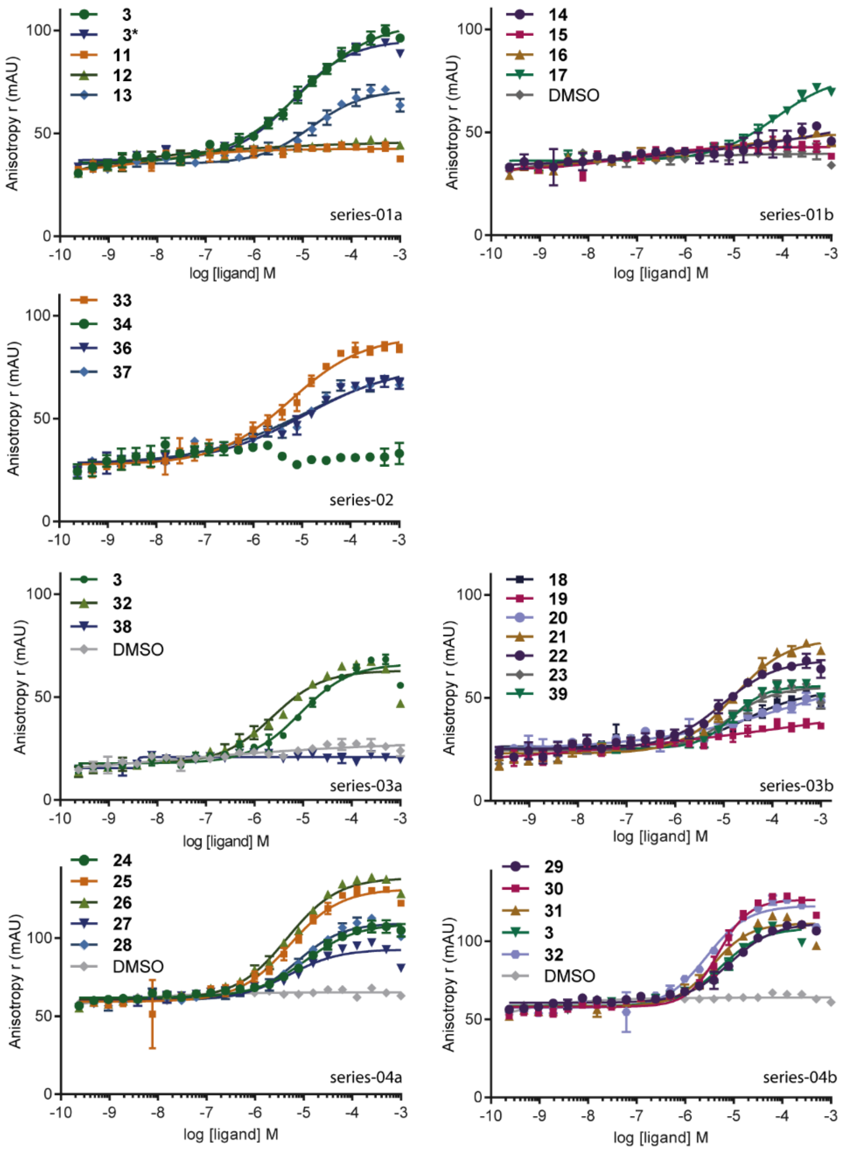

Supplementary Figure 10. Fluorescence anisotropy data for ligand titrations in SAR series of compound **C3.8** (**3**). Each series was tested in separate batches, and each graph represents data per plate of compounds for clarity. Data points and error bars represent mean ± SEM, n=3 replicates. Source data are provided as a Source Data file.

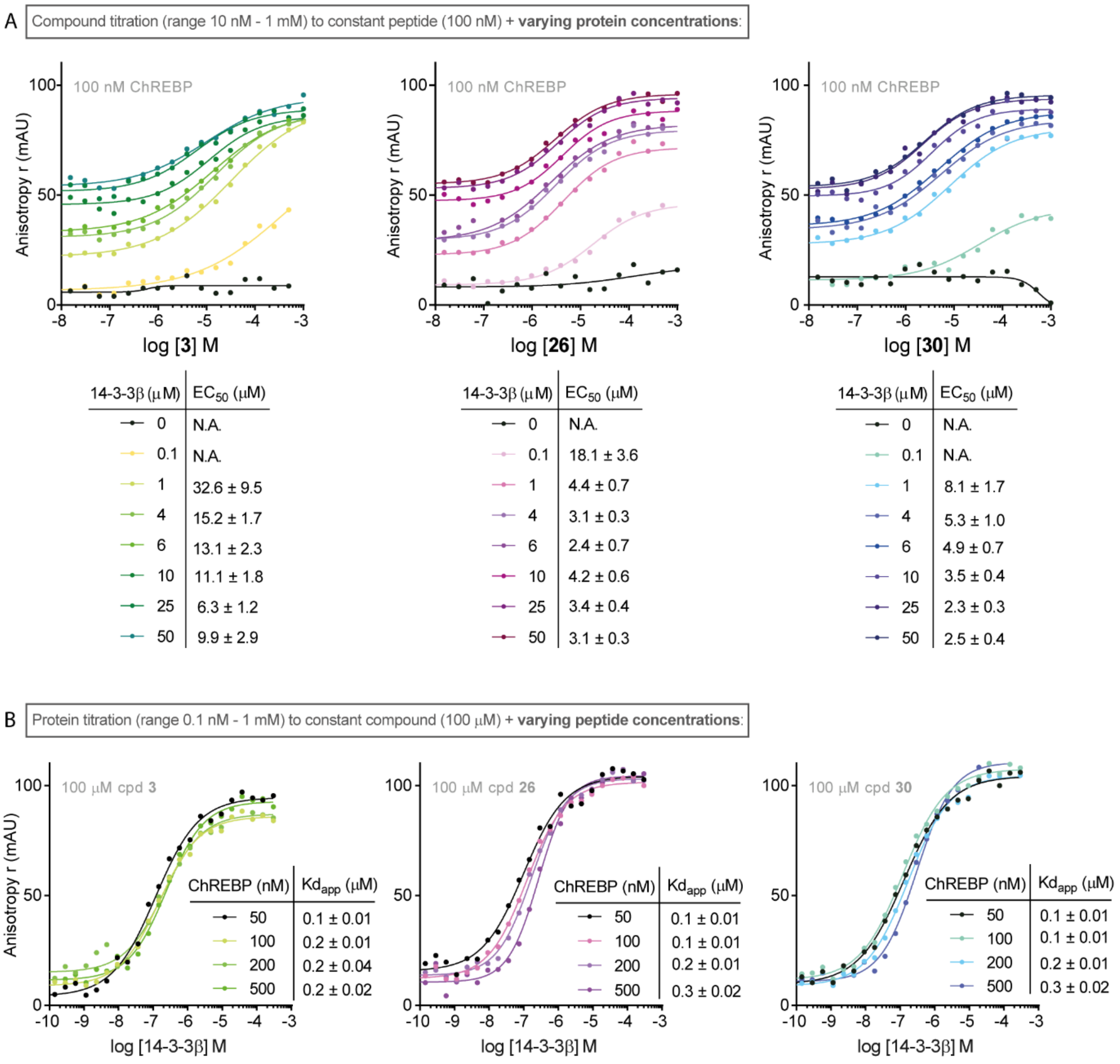

Supplementary Figure 11. Fluorescence anisotropy data for (A) ligand titrations to constant peptide (100 nM) and varying protein concentrations and (B) protein titrations to constant compound (100 μM) and varying peptide concentrations, illustrating dependency of relative components' concentrations for stabilization effect observed for the compounds. Data points and error bars represent mean ± SEM, n=3 replicates. Source data are provided as a Source Data file.

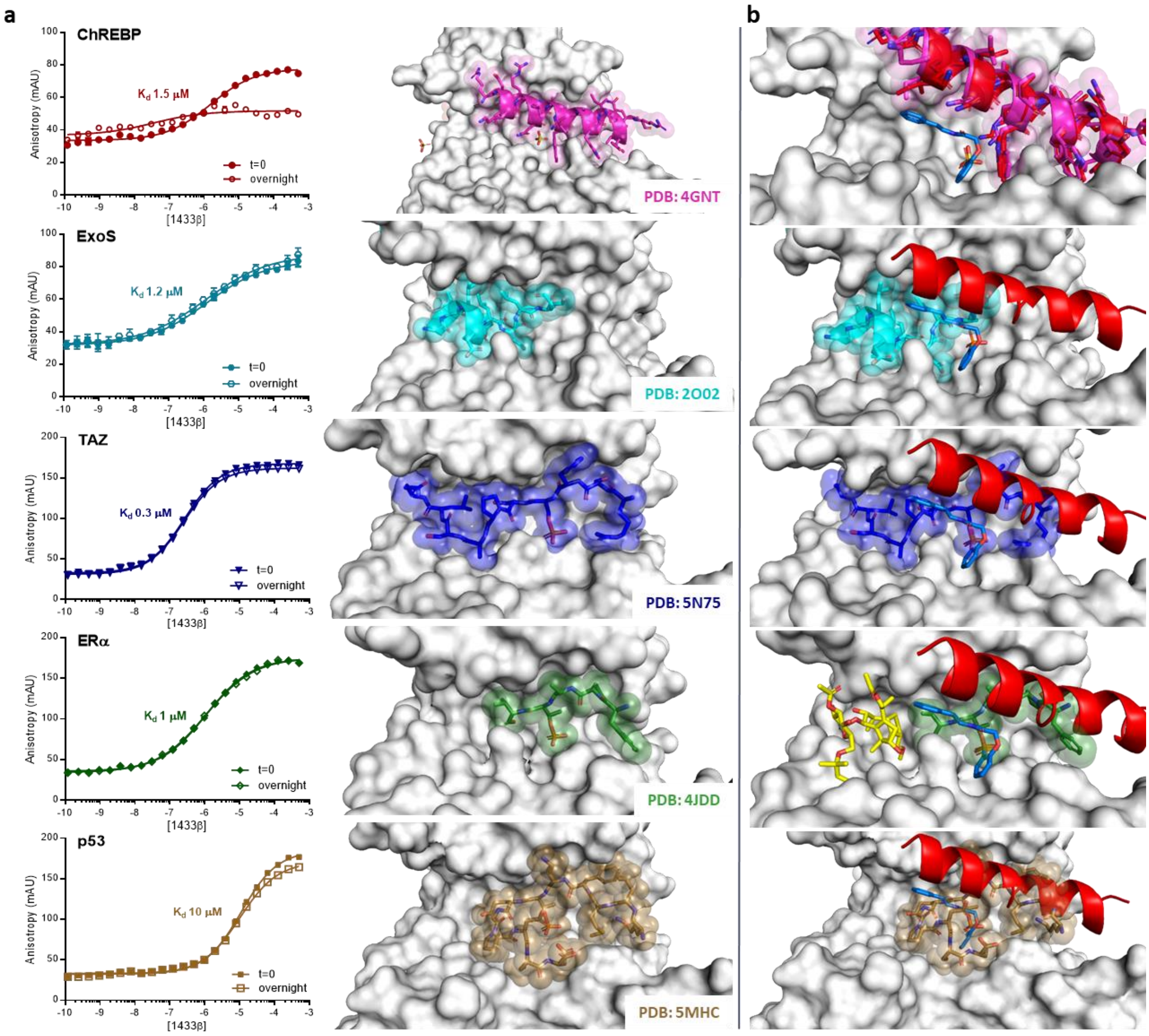

Supplementary Figure 12. Binding affinity and binding mode of representative 14-3-3 (phospho-)peptide motifs derived from ChREBP, ExoS, TAZ, ERα and p53 (Supplementary Table 5). a) Fluorescence anisotropy dose-response data for fluorescein isothiocyanate (FITC)-labeled peptides titrated with 14-3-3β and co-crystal structures for these motifs (various colors; cartoon, stick and spheres representations) binding in the 14-3-3 central groove (white surface). (PDB entries: 4GNT, 2O02, 5N75, 4JDD and 5MHC). b) Crystallographic overlays of the co-crystal structures in (a) and the crystal structure reported in this work, of 14-3-3β bound by the ChREBP α2-peptide (red cartoon) and phosphonate stabilizer **C3.8** (**3**) (blue sticks) in the phospho-accepting pocket. Data points and error bars represent mean ± SEM, n=3 replicates. Source data are provided as a Source Data file.

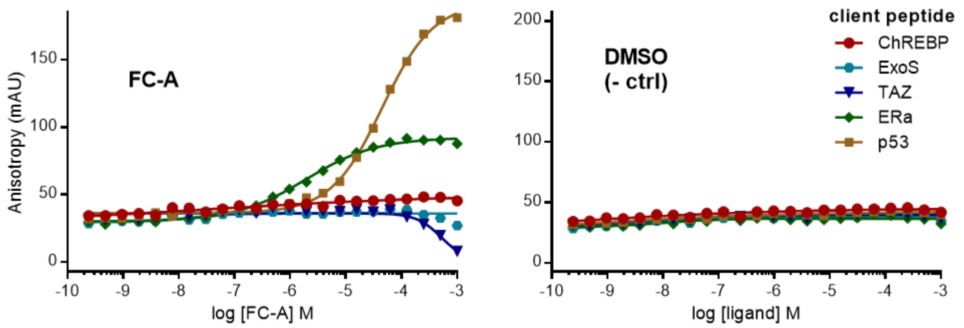

Supplementary Figure 13. Positive (FC-A) and negative (DMSO) controls for compound titrations to 14-3-3β and various client-derived FITC-labeled peptides. Data points and error bars represent mean ± SEM, n=3 replicates. Source data are provided as a Source Data file.

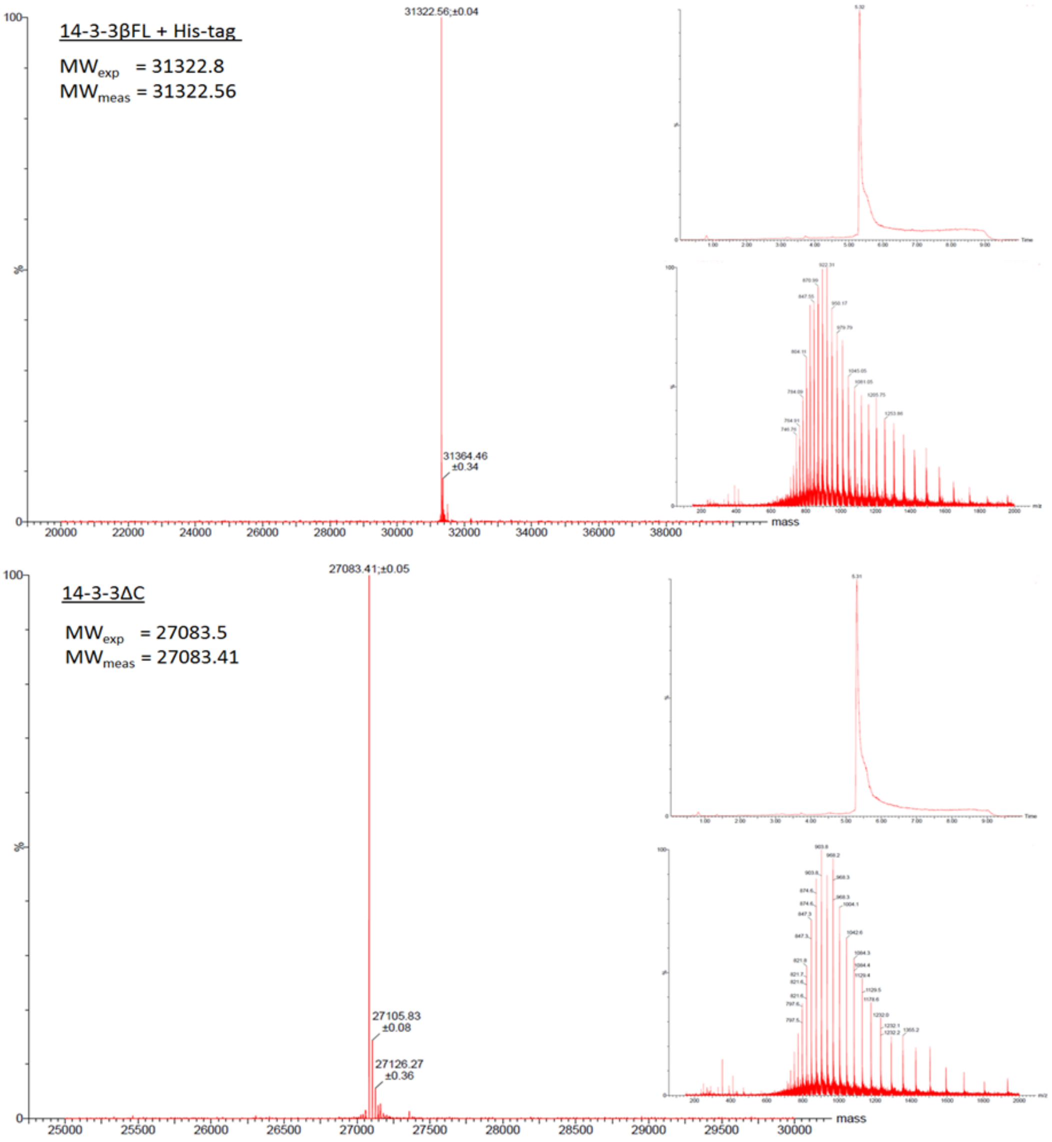

Supplementary Figure 14. Protein characterization by ultra-high resolution liquid chromatography (UPLC). Mass spectra (left), chromatogram and deconvoluted m/z spectra (right) of purified 14-3-3 proteins used in this study.

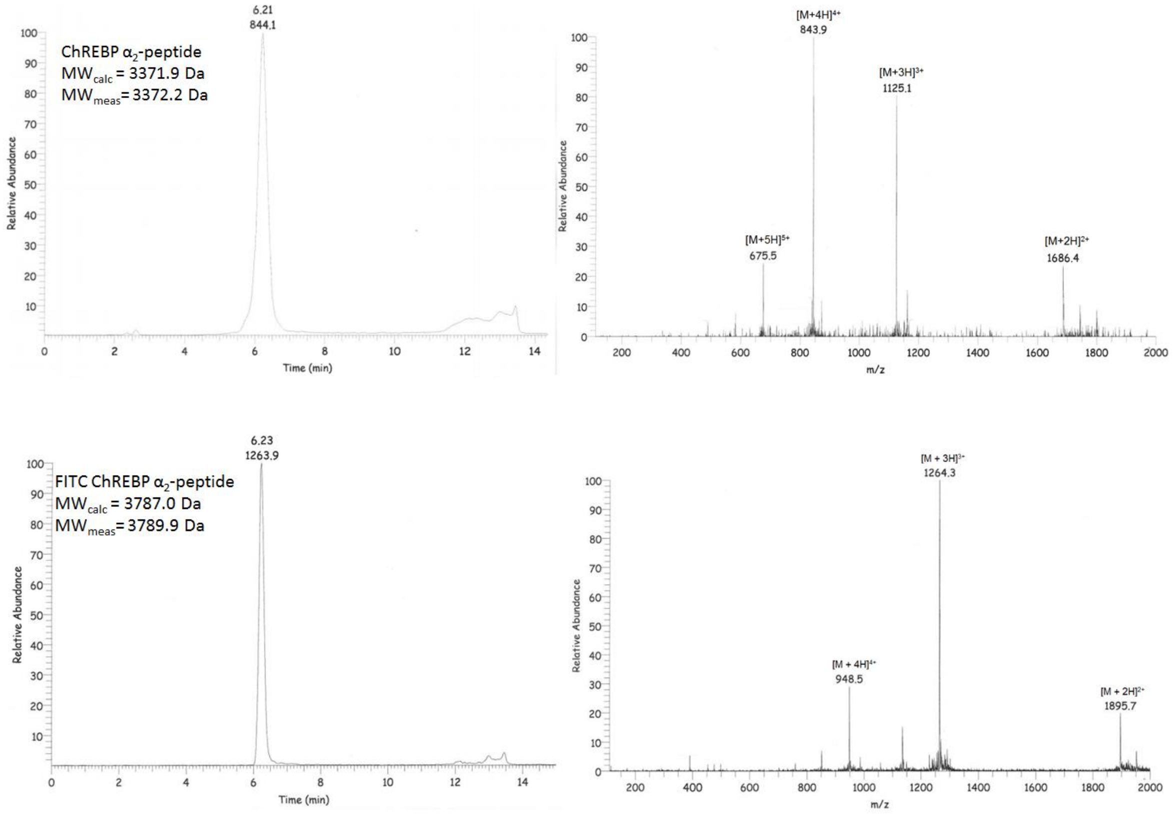

Supplementary Figure 15. Analytical LC-MS of the purified α2-peptides (acetylated and FITC-labeled). The graphs represent the total ion count chromatogram (left) and the m/z spectrum (right). The calculated mass is the monoisotopic mass.

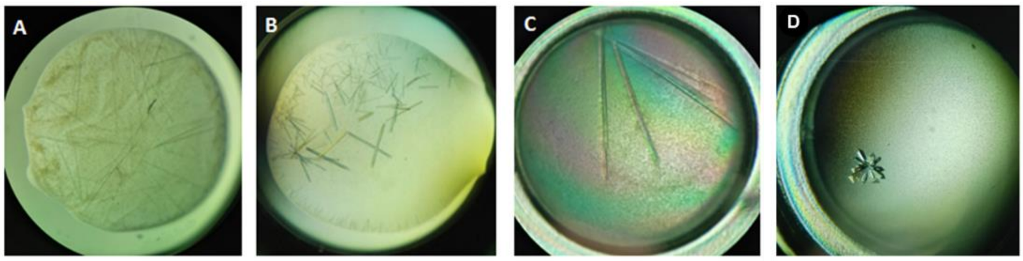

Supplementary Figure 16. First part of the optimization process of protein crystallization for the protein/peptide/compound complex consisting of 14-3-3σ ΔC / ChREBP-α2 / **3**. Needles obtained for MPD Suite #88 (A-C) were optimized for pH and PEG400 concentration and Additive Screen HT #12 resulted in three dimensional crystals, clustered around a single nucleation site.

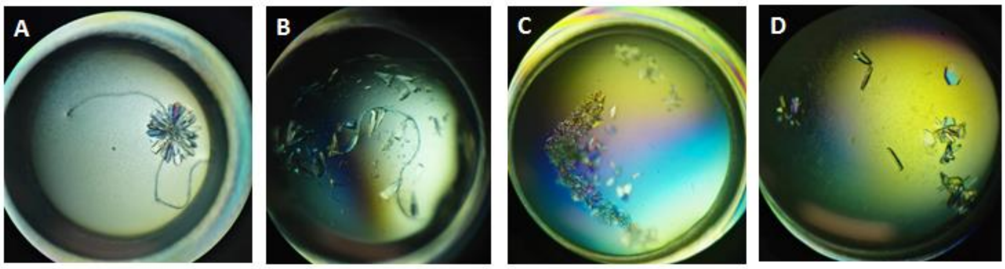

Supplementary Figure 17. Second part of the optimization process of protein crystallization for the protein/peptide/compound complex consisting of 14-3-3σ ΔC / ChREBP-α2 / **3**. Homemade crystallization-liquor (0.1 M HEPES pH 7.1, 30 % MDP, 1 % PEG4000, 0.1 M Ni(II)Cl•6H_2O) resulted in rods (A), which were crushed (B) and introduced into a fresh drop of pre-equilibrated protein complex (C). Single crystals were obtained after 1-3 days (D).

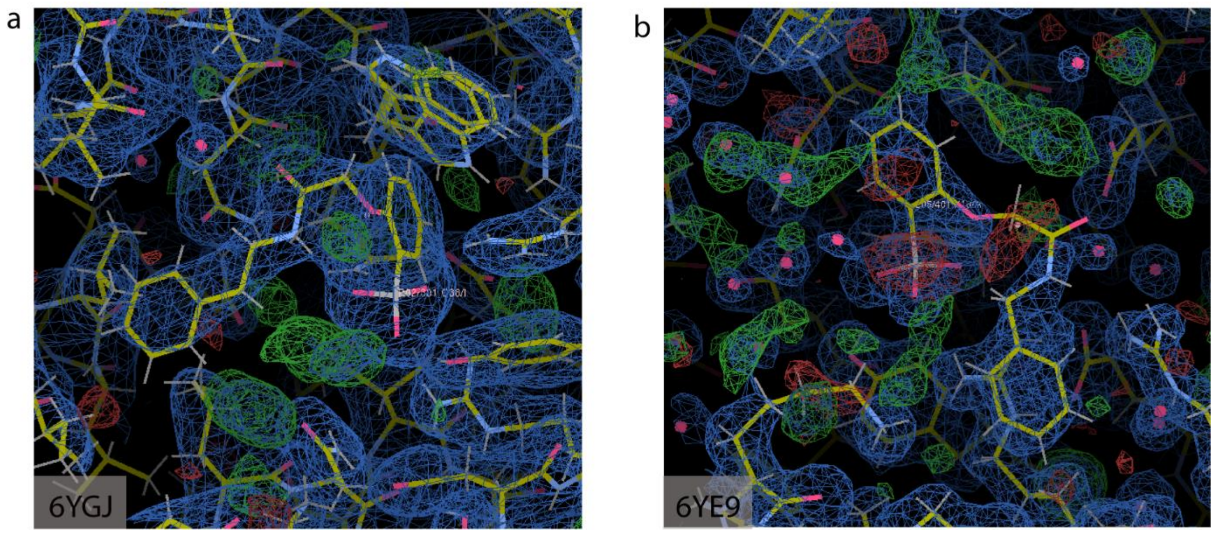

Supplementary Figure 18. Portion of the electron density maps for 6YGJ (a) and 6YE9 (b). Displayed are 2F_o-F_c (blue mesh, contoured at 1.0σ) and F_o-F_c maps (green and red meshes, contoured at 2.5σ).

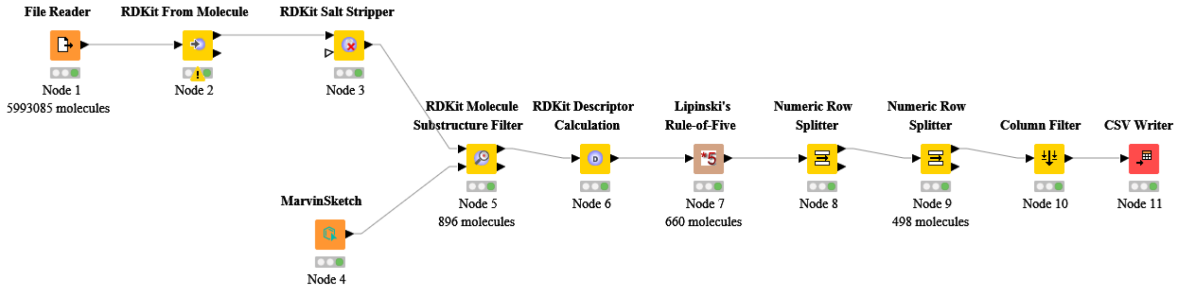

Supplementary Figure 19. Workflow in KNIME to filter the Molport library to prepare a subset for virtual screening. First, the Molport file was read by a File Reader (Node 1), followed by a conversion of the SMILES to RDkit[2] molecules (Node 2). Salts are removed by the RDKit Salt Stripper (Node 3) and the first filter is applied by a Substructure Filter (Node 5), selecting only the molecules which contain a generalized phosphonate structure drawn by MarvinSketch (Node 4). The Lipinski's Rule of Five filter (Node 7) only allows molecules meeting the Lipinski's rules. Lastly, only molecules which contain at least one ring structure are allowed by the Numeric Row Splitter (Node 9, 10).

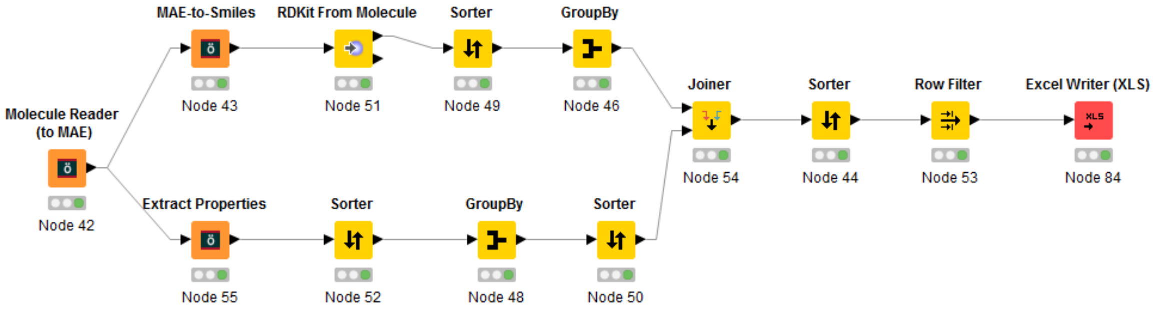

Supplementary Figure 20. KNIME workflow to extract docking results. First, the Schrödinger file was read by a Molecular reader and converted to SMILES followed by a conversion to RDKit molecules. In parallel, the docking score properties were extracted and sorted. The Name and the docking scores of the molecules were joined and sorted, and converted to an excel file.

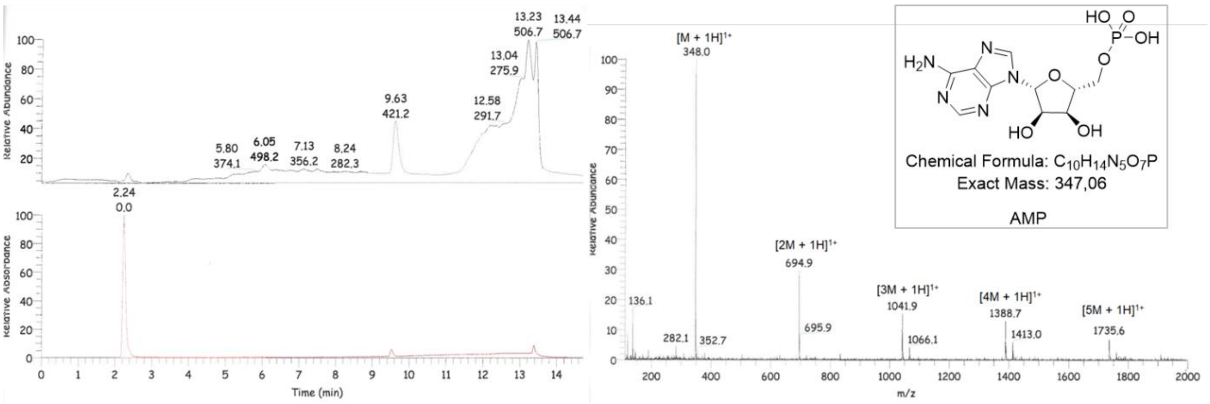

Supplementary Figure 21. Analytical LC-MS of AMP. Depicted are the total ion count chromatogram and UV-absorbance (left) and the m/z spectrum (right). The mass is the monoisotopic mass.

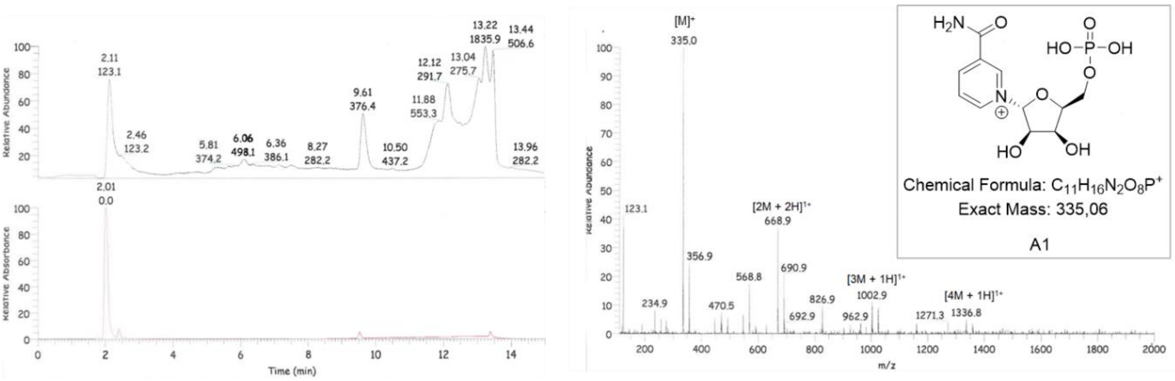

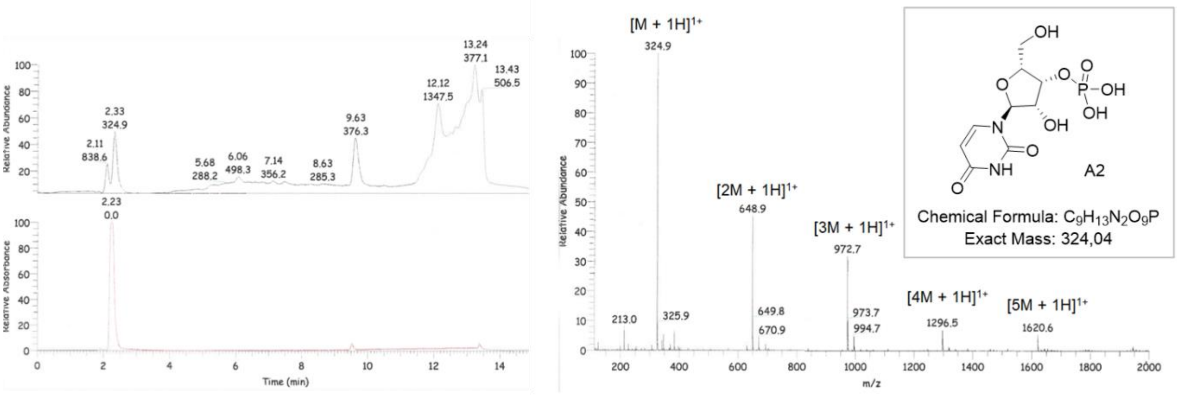

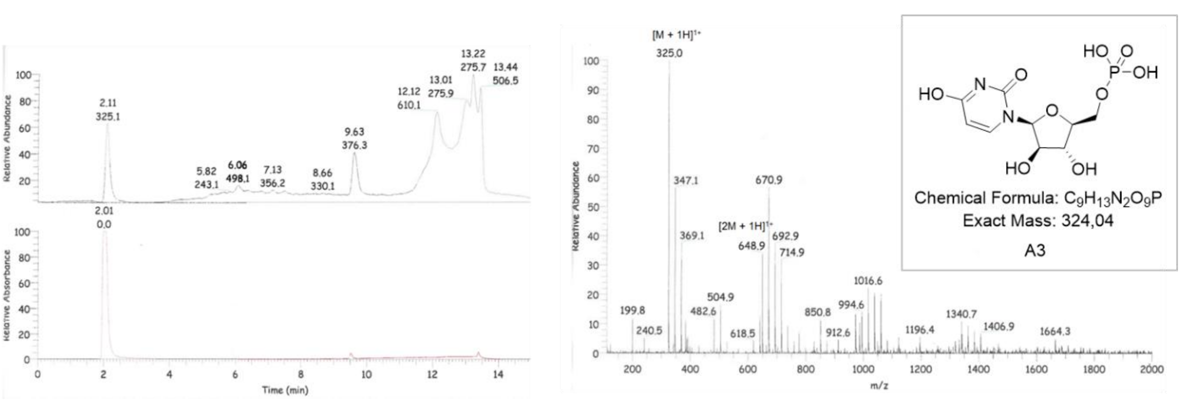

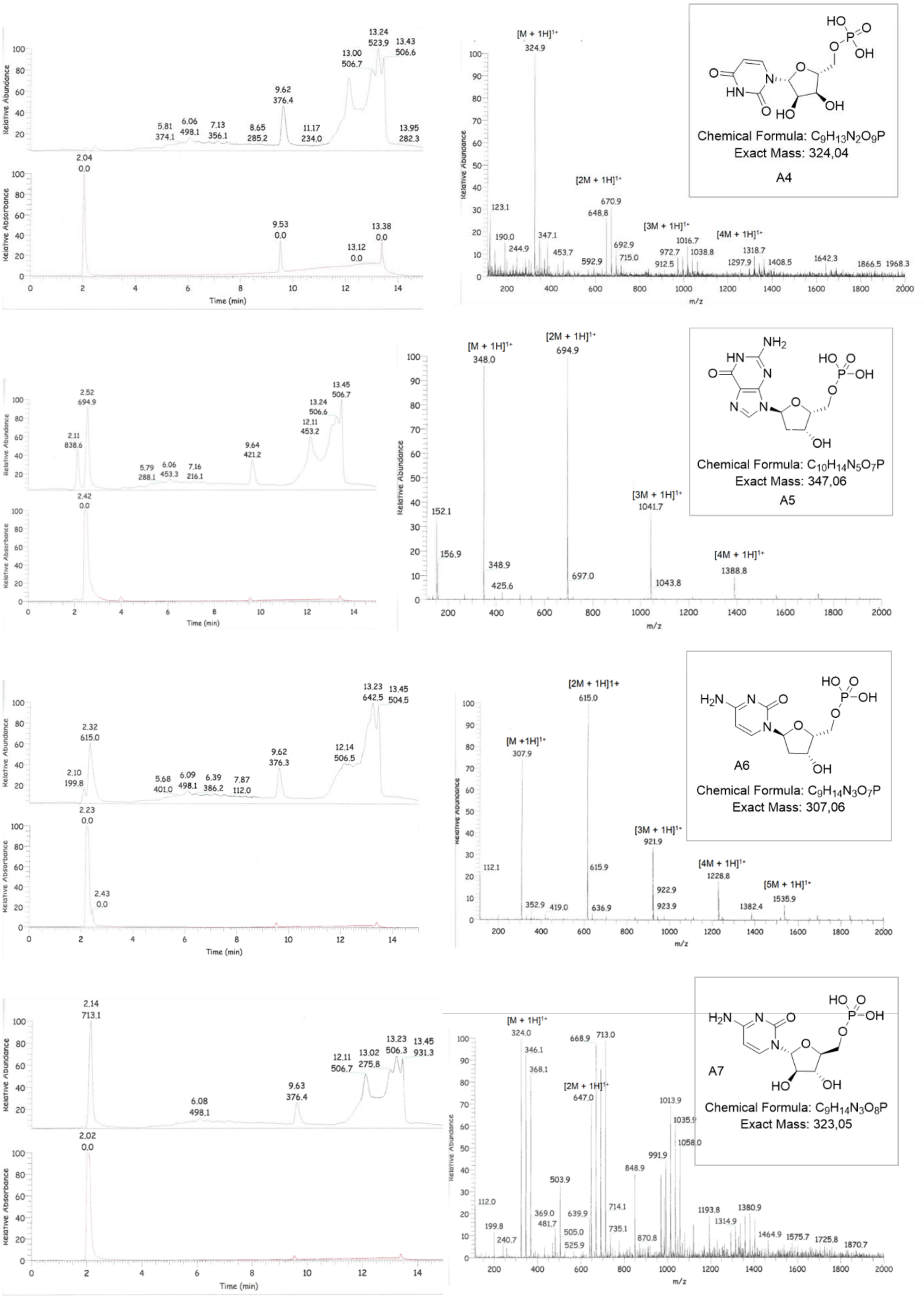

Supplementary Figure 22. Analytical LC-MS of selected compounds from docking, in class A (A1 - A7). Depicted are the total ion count chromatogram and UV-absorbance (left) and the m/z spectrum (right). The mass is the monoisotopic mass.

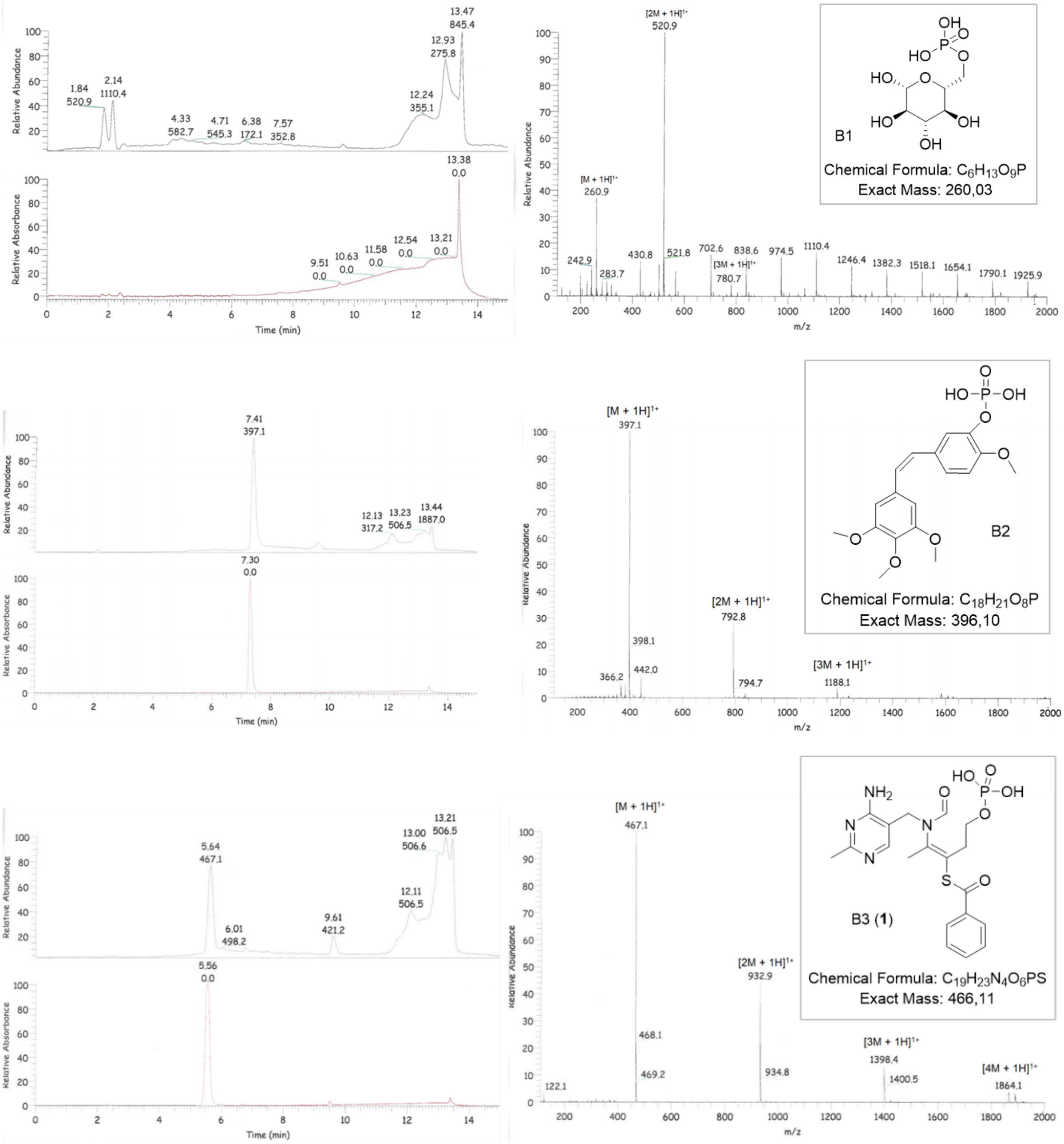

Supplementary Figure 23. Analytical LC-MS of selected compounds from docking, in class B (B1, B2, B3(=compound **1**)). Depicted are the total ion count chromatogram and UV-absorbance (left) and the m/z spectrum (right). The mass is the monoisotopic mass.

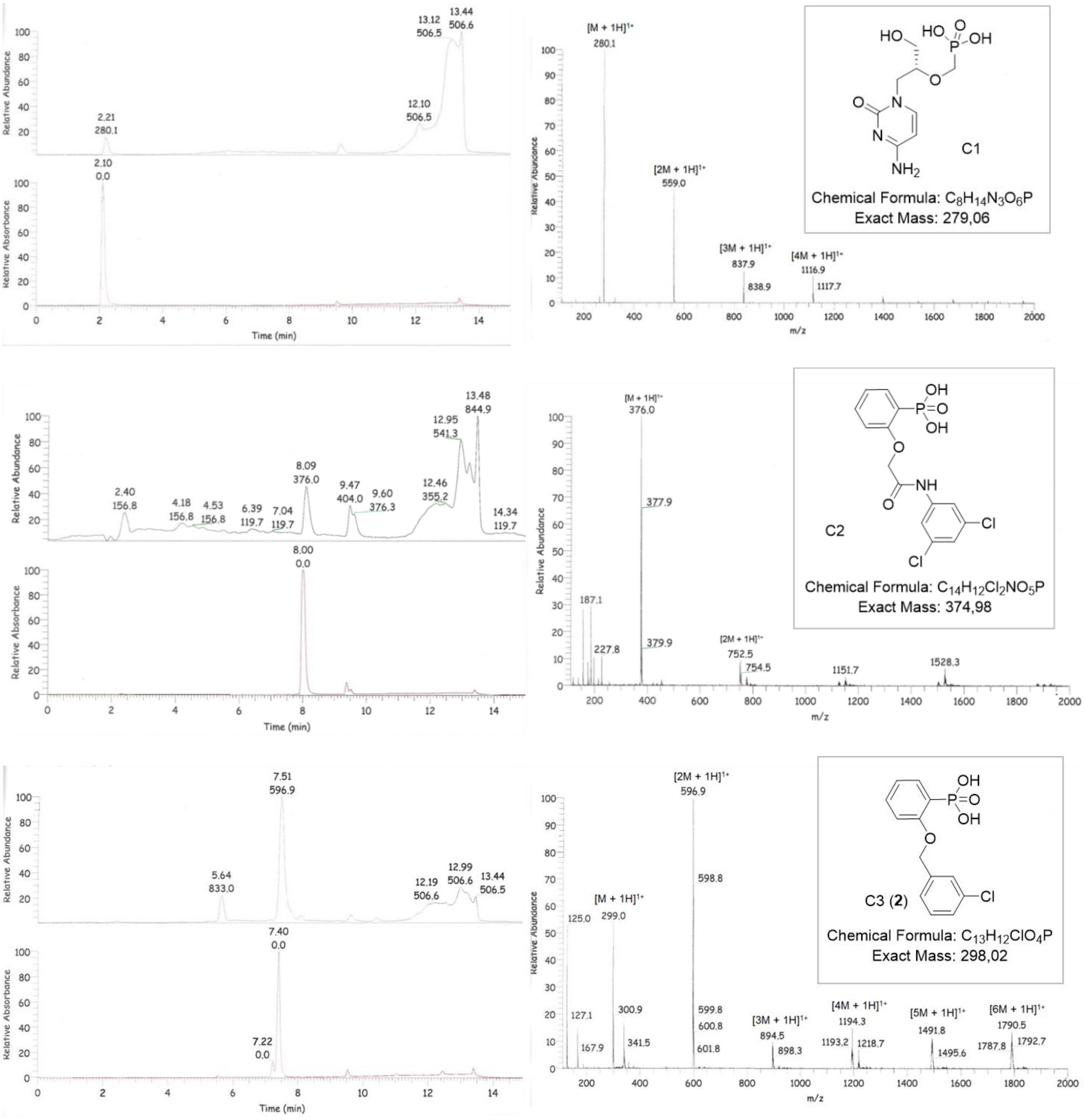

Supplementary Figure 24. Analytical LC-MS of compounds in class C (C1, C2, C3(=compound **2**)). Depicted are the total ion count chromatogram and UV-absorbance (left) and the m/z spectrum (right). The mass is the monoisotopic mass.

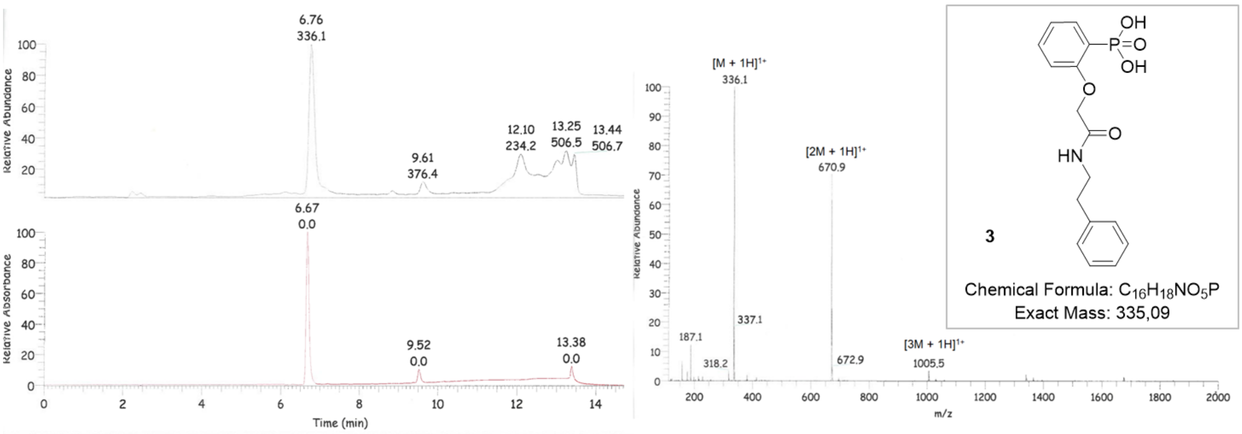
6.76
336.1
9.61
376.4
12.10
234.2
13.25
506.5
13.44
506.7
6.67
0.0
9.52
0.0
13.38
0.0
Relative Abundance
Relative Absorbance
Time (min)
[M + 1H]1+
336.1
[2M + 1H]1+
670.9
187.1
318.2
337.1
672.9
[3M + 1H]1+
1005.5
m/z
3
Chemical Formula: C16H18NO5P
Exact Mass: 335,09

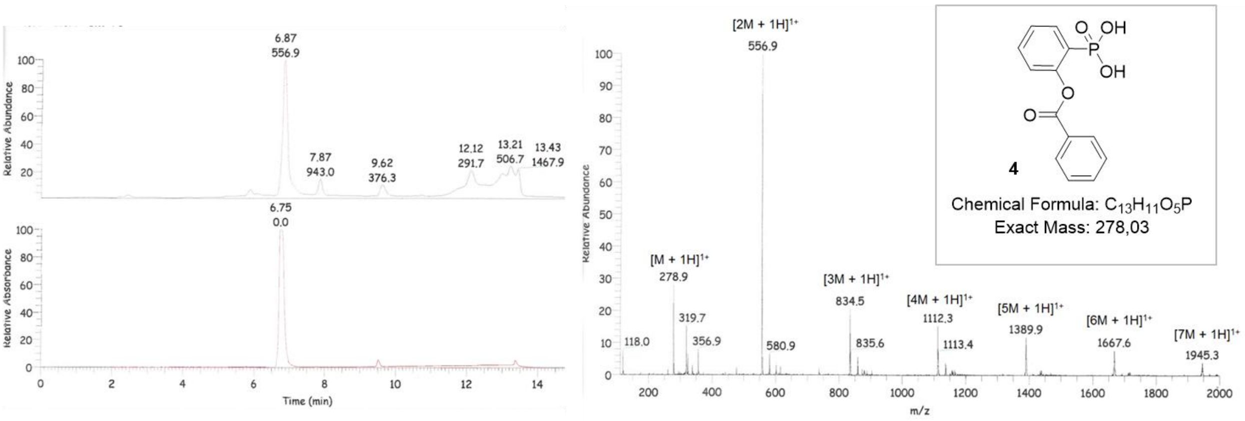
6.87
556.9
7.87
943.0
9.62
376.3
12.12
291.7
13.21
506.7
13.43
1467.9
6.75
0.0
Relative Abundance
Relative Absorbance
Time (min)
[2M + 1H]1+
556.9
[M + 1H]1+
278.9
118.0
319.7
356.9
580.9
[3M + 1H]1+
834.5
835.6
[4M + 1H]1+
1112.3
1113.4
[5M + 1H]1+
1389.9
[6M + 1H]1+
1667.6
[7M + 1H]1+
1945.3
m/z
4
Chemical Formula: C13H11O5P
Exact Mass: 278,03

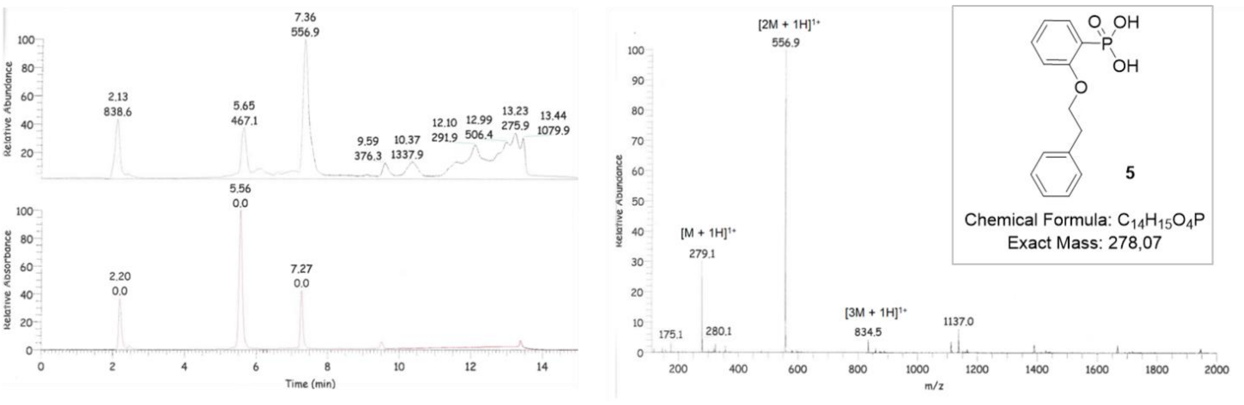
2.13
838.6
5.65
467.1
7.36
556.9
9.59
376.3
10.37
1337.9
12.10
291.9
12.99
506.4
13.23
275.9
13.44
1079.9
5.56
0.0
2.20
0.0
7.27
0.0
Relative Abundance
Relative Absorbance
Time (min)
[2M + 1H]1+
556.9
[M + 1H]1+
279.1
175.1
280.1
[3M + 1H]1+
834.5
1137.0
m/z
5
Chemical Formula: C14H15O4P
Exact Mass: 278,07

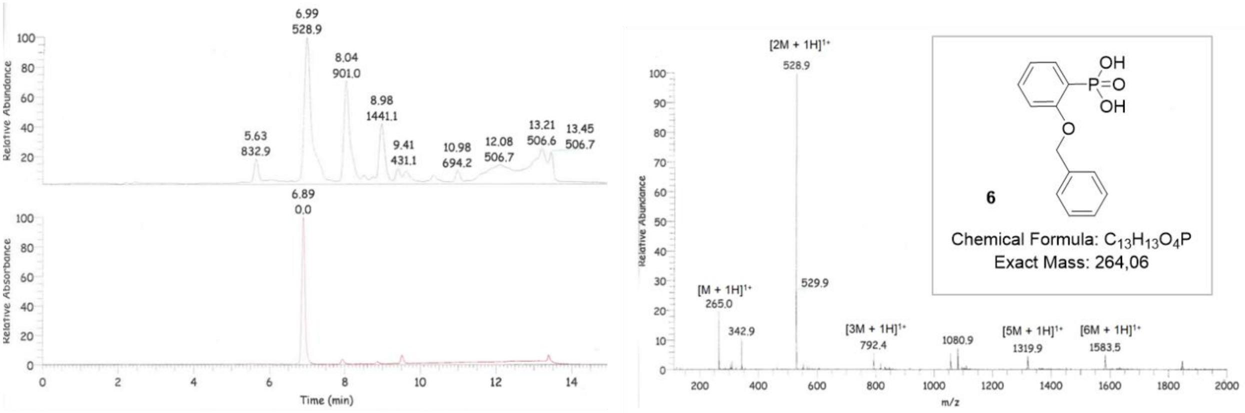
6.99
528.9
8.04
901.0
8.98
1441.1
5.63
832.9
9.41
431.1
10.98
694.2
12.08
506.7
13.21
506.6
13.45
506.7
6.89
0.0
Relative Abundance
Relative Absorbance
Time (min)
[2M + 1H]1+
528.9
[M + 1H]1+
265.0
342.9
529.9
[3M + 1H]1+
792.4
1080.9
[5M + 1H]1+
1319.9
[6M + 1H]1+
1583.5
m/z
6
Chemical Formula: C13H13O4P
Exact Mass: 264,06

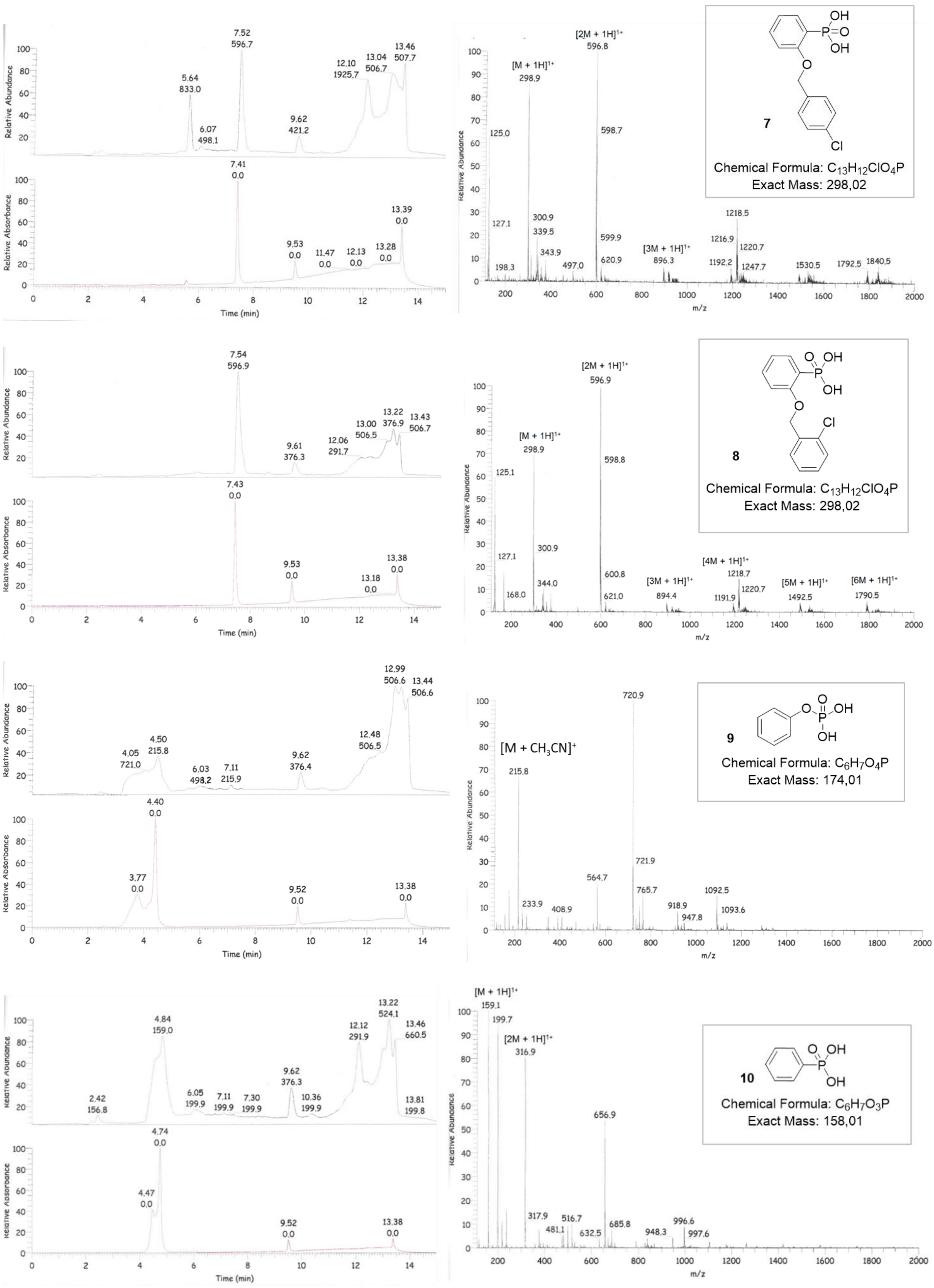

Supplementary Figure 25. Analytical LC-MS of SAR-by-catalog derivatives of C3 (**2**): (compounds **3** - **10**). Depicted are the total ion count chromatogram and UV-absorbance (left) and the m/z spectrum (right). The mass is the monoisotopic mass.

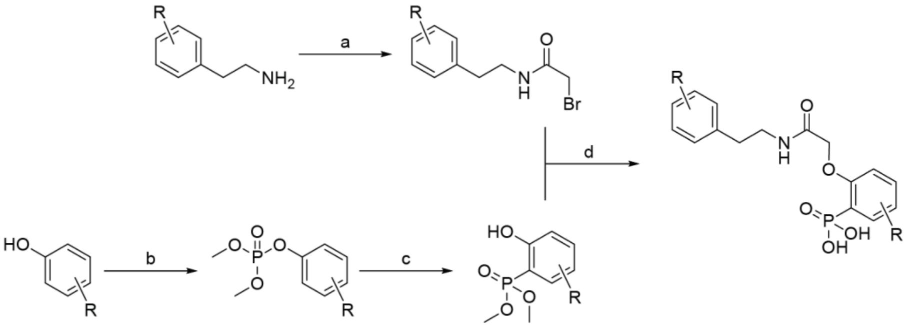

Supplementary Figure 26. Chemical synthesis of derivatives **18 – 29**, **33 – 37** and **38**. Reagents and conditions: a) Bromoacetyl bromide, Et_3N, DCM (dry), 0 °C to rt, 16 h, b) $HPO(OMe)_2$, CCl_4, Et_3N, DCM (dry), 0 °C to rt, 16 h, c) *n*-BuLi, DIPA, THF (dry), -78 °C to rt, 16 h, d) i) K_2CO_3, acetone, rt, 16 h, ii) TMSBr, DCM (dry), 0 °C to rt, 4 h, iii) MeOH/H_2O (3:1), rt, 1 h.

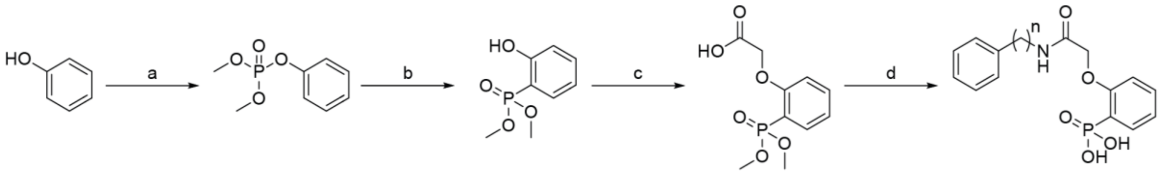

Supplementary Figure 27. Chemical synthesis of derivatives **3** and **11 – 13**. Reagents and conditions: a) $HPO(OMe)_2$, CCl_4, Et_3N, DCM (dry), 0 °C to rt, 16 h, b) *n*-BuLi, DIPA, THF (dry), -78 °C to rt, 16 h, c) i) Methyl bromoacetate, K_2CO_3, acetone, rt, 16 h, ii) LiOH/THF (1:1), rt, 16 h, e) i) amines, EDC, HOBt, DIPEA, DCM, rt, 16 h, ii) TMSBr, DCM (dry), 0 °C to rt, 4 h, iii) MeOH/H_2O (3:1), rt, 1 h.

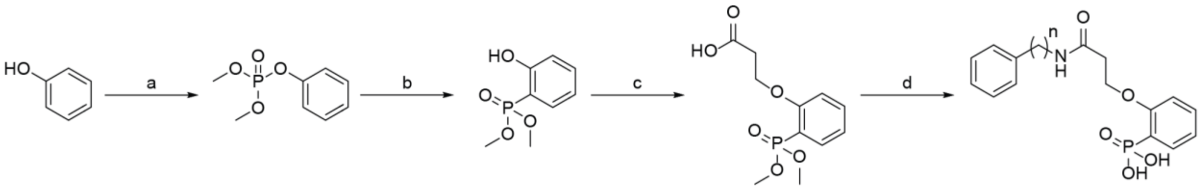

Supplementary Figure 28. Chemical synthesis of derivatives **14 – 17**. Reagents and conditions: a) $HPO(OMe)_2$, CCl_4, Et_3N, DCM (dry), 0 °C to rt, 16 h, b) *n*-BuLi, DIPA, THF (dry), -78°C to rt, 16 h, c) *beta*-Propiolactone, KO*t*Bu, THF (dry), rt, 16 h, e) i) amines, EDC, HOBt, DIPEA, DCM, rt, 16 h, ii) TMSBr, DCM (dry), 0°C to rt, 4 h, iii) MeOH/H_2O (3:1), rt, 1 h.

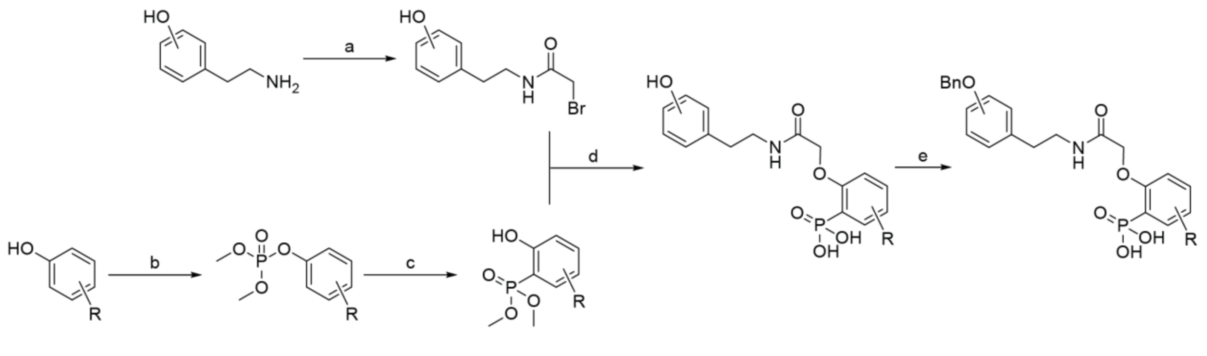

Supplementary Figure 29. Chemical synthesis of derivatives **30 – 32**. Reagents and conditions: a) Bromoacetyl bromide, Et_3N, DCM (dry), 0 °C to rt, 16 h, b) $HPO(OMe)_2$, CCl_4, Et_3N, DCM (dry), 0 °C to rt, 16 h, c) *n*-BuLi, DIPA, THF (dry), -78 °C to rt, 16 h, d) K_2CO_3, acetone, rt, 16 h, e) i) Benzyl bromide, K_2CO_3, acetone, rt, 16 h, ii) TMSBr, DCM (dry), 0 °C to rt, 4 h, iii) MeOH/H_2O (3:1), rt, 1 h.

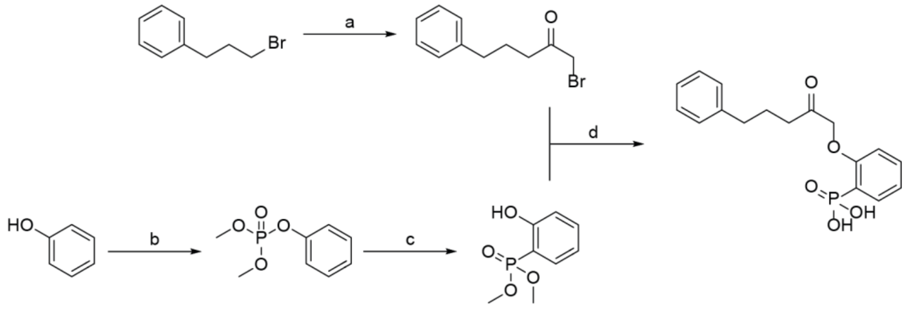

Supplementary Figure 30. Chemical synthesis of derivative **39**. Reagents and conditions: a) i) Mg, $Br(CH_2)_2Br$, Et_2O (dry), reflux, 2 h, ii) Bromoacetyl chloride, Et_2O (dry), 0 °C to rt, 16 h, b) $HPO(OMe)_2$, CCl_4, Et_3N, DCM (dry), 0 °C to rt, 16 h, c) *n*-BuLi, DIPA, THF (dry), -78 °C to rt, 16 h, d) i) K_2CO_3, acetone, rt, 16 h, ii) TMSBr, DCM (dry), 0 °C to rt, 4 h, iii) $MeOH/H_2O$ (3:1), rt, 1 h.

Supplementary Methods

Virtual Screening and Molecular Docking Procedures

Filtering of the Molport library. The Molport library (release date 04-08-2017, 5,993,085 compounds) was screened by implementing it as a single workflow in KNIME Analytics Platform.[3,4] KNIME is a modular data mining platform where interface data can be directed down to certain workflows, from a source input node through other nodes, which then process the data (Supplementary Figure 19).

Rigid receptor docking. For the rigid receptor docking software tools from the Molecular Modeling Platform of Schrödinger were used. The crystal structure of the 14-3-3β/ChREBP complex, stabilized by AMP (PDB entry 5F74) with a resolution of 2.35 Å, was used to create a receptor grid. The receptor was prepared using the *Protein Preparation Wizard*. AMP was removed from the grid and used as a reference position for ligand docking. The surrounding waters were kept for optimization of the hydrogen bond network and were deleted afterwards. The compound selection was prepared using *Ligprep*. The docking was performed with *Glide* in XP mode.[5,6] The top 200-ranked poses were visually inspected according to their docking score, and an excel graph was made using KNIME to convert the docking scores (Supplementary Figure 20). Finally, 13 compounds were selected for experimental validation. The primary selection criterion was the precise overlap of the phosphate or phosphonate group of the docked compounds with the phosphate of AMP**.**

Flexible receptor docking. The 13 compounds selected from the rigid receptor docking, were docked into the 14-3-3/ChREBP complex using the *Induced Fit* protocol of Schrödinger.[6–8] The Box center needed for the docking was created by selecting AMP in the already prepared receptor. In the first stage of the IFD protocol, the *Glide Docking*, 20 initial poses are generated using softened-potential docking. This docking consisted of scaling the van der Waals radii by 0.5. These top 20 poses underwent a full cycle of protein refinement by *Prime Refinement*. First, a list is generated of all residues having at least one atom within 5.0 Å of an atom in any of the 20 ligand poses. Subsequently, these side chains underwent a conformational search and a minimization. These complexes were ranked by *Prime energy* and those with 30 kcal/mol of the minimum energy structure were redocked using *Glide docking* and scored by *GlideScore.*

Purchasing of ligands. The ligands selected from the docking studies (categorized in class A, B and C, including **1** and **2**) and the initial SAR-by-catalog study (compounds **3** - **10**) were obtained from Molport and purity was confirmed by analytical liquid chromatography coupled with mass spectrometry (LC-MS), performed on a C4 Jupiter SuC4300A 150 x 2.0 mm column (using a 15 min. gradient of 5 % to 100 % acetonitrile in H2O (0.1 % formic acid)), connected to a ThermoFischer LCQ Fleet Ion Trap Mass Spectrometer, see Supplementary Figures 21-25.

For description of custom organic synthesis and characterization of the library of analogs (Table 2 and Figure 4 of the main manuscript); compounds **11** – **39** and intermediates, see next section of the Supplementary Methods,Organic Synthesis and Characterization, pages S26 – S41.

Organic Synthesis and Characterization

Synthetic procedures (see Supplementary Figures 26 – 30 for general schemes) were based on previously published work by the Kaiser group.[9]

General Information (chemicals, materials and instrumentation)

Reagents and dry solvents

All reagents were purchased from *ABCR*, *Acros Organics*, *Alfa Aesar*, *Carbolution Chemicals*, *Carl Roth*, *Fisher*, *Fluka*, *Fluorochem*, *Merck*, *Riedel de Häen*, *Sigma Aldrich*, *TCI Chemicals*, *Bernd Kraft* or *VWR Chemicals* and were used without further purification. All dry solvents were purchased from the same supplier with the best quality available.

Column chromatography

Compound purification by column chromatography was achieved using glass columns filled with silica gel (particle size 35 – 70 µm, from *Acros Organics*) as stationary phase and eluent mixtures of different solvents as mobile phase. The exact ratios of the solvents are listed in the corresponding synthesis procedures.

Thin layer chromatography (TLC)

Thin layer chromatography was performed on silica coated aluminum plates (60 F_{254}) from *Merck*. Detection of substances was conducted with UV light (wave length 254 nm or 366 nm). The resulting R_f values including the used solvents are listed in the corresponding synthesis procedures.

Freeze-drying

Freeze-drying of the products was carried out with a lyophilizer ALPHA 2-4 LD plus (CHRIST) at an ice condenser temperature of -80 °C. The drying process is favored by a large ice surface and a low ice thickness. To obtain the largest possible ice surface, an aqueous solution of the corresponding substance was frozen in liquid nitrogen under constant rotation. The corresponding frozen compounds were then put for 24 – 72 h on the lyophilizer.

Reversed-phase liquid chromatography electrospray ionization mass spectrometry (LC-MS)

Reaction control analyses were performed on a LC-MS system from *Thermo Scientific*. The system consisted of a Thermo Scientific Accela™ (peak detection at 210 nm) and a Thermo Scientific UltiMate™ 3000 (peak detection at 230 nm and 260 nm) equipped with an Eclipse XDB-C18 column (particle size 5 µm, from Agilent) and a *Thermo Scientific* LCQ Fleet™ ESI-MS. For analysis, a linear gradient of solvent B (0.1 % formic acid in acetonitrile) in solvent A (0.1 % formic acid in water) at flow rate of 1 mL min^{-1} and the following gradient program: 0 min (10 % B) → 1 min (10 % B) → 10 min (100 % B) → 12 min (100 % B) → 15 min (10 % B) was used.

High-resolution mass spectrometry (HRMS)

HRMS spectra were recorded on an Exactive Plus EMR mass spectrometer from *Thermo Fisher with* an Advion TriVersa NanoMate ESI system from *Advion.*

Preparative reversed-phase high performance liquid chromatography (prep HPLC)

Compound purification by HPLC was achieved using the *Prominence UFLC* system from *Shimadzu* (peak detection at 210 nm and 254 nm). The system was equipped with a reversed-phase C18 column from *Phenomenex* (*Luna*® 5 µm C18(2), 100 x 21.20 mm). For purification a linear gradient of solvent B (0.1 % TFA in acetonitrile) in solvent A (0.1 % TFA in water) at a flow rate of 20 mL min^{-1} was used.

Nuclear magnetic resonance spectroscopy (NMR)

Nuclear magnetic resonance (NMR) spectra were recorded on a *Bruker Avance II* 400 (400 MHz for ^{1}H NMR and 100 MHz for ^{13}C NMR) machine. As solvents deuterated chloroform-d_1, deuterated DMSO-d_6 or deuterated methanol-d_4 were used. The chemical shifts δ are reported in parts per million (ppm). The spectra were referenced to the residual signals of undeuterated solvents ($CDCl_3$: δ (^{1}H) = 7.26 and δ (^{13}C) = 77.16, DMSO: δ (^{1}H) = 2.50 and δ (^{13}C) = 39.52, MeOD: δ (^{1}H) = 4.87 and δ (^{13}C) = 49.00). The coupling constants *J* are reported in Hertz (Hz). The ^{1}H NMR spectral data list the chemical shifts δ, the multiplicities (s: singlet, d doublet, t: triplet, m: multiplet), the coupling constant *J* and the number of protons. The ^{13}C NMR spectra list only the chemical shifts δ.

General Methods

General Method A: Synthesis of the phosphate derivative

The corresponding phenol derivative (1.0 eq) was dissolved in dry DCM (0.5 mL per mmol). Tetrachlormethane (1.0 eq) and triethylamine (1.0 eq) were added. The solution was cooled to 0 °C and dimethyl phosphite (1.5 eq) was added dropwise. The resulting solution was stirred for 16 h, allowing the mixture to slowly reach room temperature. The reaction mixture was washed with 5% $NaHCO_3$ solution (3x), the organic phase was dried over $MgSO_4$ and was removed under reduced pressure. The crude product was purified by column chromatography.

General Method B: Synthesis of the phosphonate derivative

Generation of LDA: Dry di-*iso*propyl amine (1.7 eq) was dissolved in dry THF (4.8 mL per mmol of the phosphate derivative) and cooled to -78 °C. *n*-BuLi (2.5 M, 1.5 eq) was added dropwise. The cooling bath was removed and the mixture was stirred for 1 h.

The LDA mixture was cooled to -78 °C again. The respective phosphate derivative (1.0 eq) was dissolved in dry THF (0.7 mL per mmol of the phosphate derivative), cooled to -78 °C and added to the LDA solution. The resulting mixture was stirred for 16 h, allowing the mixture to slowly reach room temperature. The reaction mixture was quenched with saturated NH_4Cl solution (0.6 mL per mmol of the phosphate derivative), the organic phase was separated and was removed under reduced pressure. The crude product was purified by column chromatography.

General Method C: Amide coupling with HOBt and EDC

The carboxylic acid derivative (1.0 eq) was dissolved in DCM (50 mL per mmol of the carboxylic acid derivative). EDC (4.0 eq), HOBt (4.0 eq) and DIPEA (6.0 eq) were added. The corresponding amine (2.0 eq) was added and the mixture was stirred for 16 h at room temperature. The reaction mixture was washed with 5% $KHSO_4$ solution (3x) and 5% $NaHCO_3$ solution (3x). The organic phase was dried over $MgSO_4$ and was removed under reduced pressure.

General Method D: Amide coupling with bromoacetyl bromide

The amine derivative (1.0 eq) was dissolved in dry DCM (5 mL per mmol) and cooled to 0 °C. Triethylamine (1.1 eq) and bromoacetyl bromide (1.0 eq) were added and the resulting mixture was stirred for 16 h, allowing the mixture to slowly reach room temperature. The reaction mixture was washed with saturated NH_4Cl solution (3x) and the organic phase was dried over $MgSO_4$ and removed under reduced pressure. The crude product was purified by HPLC.

General Method E: Williamson ether synthesis

The respective phosphonate derivative (1.0 eq) was dissolved in acetone (25 mL per mmol of the phosphonate derivative). Potassium carbonate (2.0 eq) was added and the resulting suspension was stirred for 20 min at room temperature. The respective bromide (1.1 eq) was added and the resulting mixture was stirred for 16 h, allowing the mixture to slowly reach room temperature. The solvent was evaporated and the residue was suspended in ethyl acetate (25 mL per mmol of the phosphonate derivative). This suspension was washed with saturated NH_4Cl solution (3x) and the organic phase was dried over $MgSO_4$ and removed under reduced pressure.

General Method F: Deprotection

The protected phosphonate derivative (1.0 eq) was dissolved in dry DCM (6 mL per mmol) and cooled to 0 °C. TMSBr (5.0 eq) was added and the resulting solution was stirred for 4 h, allowing the mixture to slowly reach room temperature. The solvent was removed under reduced pressure. The residue was re-dissolved in a mixture MeOH/H_2O (3:1, 6 mL per mmol of the protected phosphonate derivative) and stirred for 1 h at room temperature. The solvent was removed under reduced pressure and the crude product was purified by HPLC.

Detailed Synthetic Procedures and Characterization of Compounds

Synthesis of dimethyl phenyl phosphate (**SI-1**)

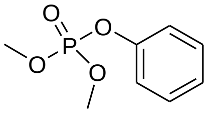

SI-1

The phosphate **SI-1** was synthesized via general method A using phenol (9.41 g, 100 mmol), tetrachlormethane (9.6 mL, 100 mmol), triethylamine (14.9 mL, 100 mmol) and dimethyl phosphite (13.8 mL, 150 mmol) in dry DCM (50 mL). The crude product was purified by column chromatography (cyclohexane/ethyl acetate 2:1 → 1:1) to obtain **SI-1** (11.82 g, 58.5 mmol, 59 %) as a colorless oil. **TLC** (cyclohexane:ethyl acetate, 1:1 v/v): $R_f = 0.4$. **LC-MS (ESI)**: $t_R = 7.13$ min; *m/z* = 203.03 $[M + H]^+$. **^{1}H NMR** (400 MHz, $CDCl_3$): δ = 7.26 – 7.22 (m, 2H), 7.13 – 7.06 (m, 3H), 3.73 (s, 6H). **^{13}C NMR** (101 MHz, $CDCl_3$): δ = 150.81, 129.95, 125.33, 120.05, 120.00, 55.01. **HRMS (ESI)**: *m/z* = 203.0468 calcd. for $[C_8H_{11}O_4P + H]^+$; found: 203.0462.

Synthesis of dimethyl 2-hydroxyphenylphosphonate (**SI-2**)

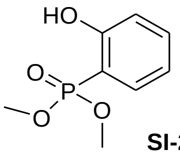

SI-2

The phosphonate **SI-2** was synthesized via general method B using di-*iso*propyl amine (13.8 mL, 98.5 mmol), *n*-BuLi (2.5 M, 34.8 mL, 87.8 mmol) and phosphate **SI-1** (11.80 g, 58.4 mmol) in dry THF (280 mL + 40 mL). The crude product was purified by column chromatography (cyclohexane/ethyl acetate 2:1) to obtain **SI-2** (9.92 g, 49.1 mmol, 84 %) as a white solid. **TLC** (cyclohexane:ethyl acetate, 2:1 v/v): $R_f = 0.4$. **LC-MS (ESI)**: $t_R = 6.02$ min; *m/z* = 202.98 $[M + H]^+$. **^{1}H NMR** (400 MHz, $CDCl_3$): δ = 10.06 (s, 1H), 7.47 – 7.43 (m, 1H), 7.35 (ddd, *J* = 14.3, 7.7, 1.7 Hz, 1H), 6.99 – 6.90 (m, 2H), 3.74 (d, *J* = 11.6 Hz, 6H). **^{13}C NMR** (101 MHz, $CDCl_3$): δ = 162.77, 135.94, 132.01, 120.22, 118.29, 77.80, 77.16, 53.47. **HRMS (ESI)**: *m/z* = 203.0468 calcd. for $[C_8H_{11}O_4P + H]^+$; found: 203.0461.

Synthesis of the derivatives 3, 11 - 13

Synthesis of 2-(2-(methoxyphosphono)phenoxy)acetic acid (**SI-3**)

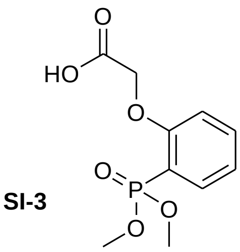

SI-3

The phosphonate derivative **SI-3** was synthesized via general method E using phosphonate **SI-2** (1.822 g, 9.00 mmol), potassium carbonate (2.484 g, 18.0 mmol) and methyl bromoacetate (0.95 mL, 9.90 mmol) in acetone (225 mL). The crude intermediate was dissolved in a mixture THF/ methanol (1:1, 300 mL). Lithium hydroxide (0.647 g, 27.0 mmol, 3.0 eq) was added and the solution was stirred for 16 h at room temperature. The reaction mixture was acidified with 1M HCl solution (to a pH < 3) and the solvent was evaporated under reduced pressure. The crude product was purified by column chromatography (ethyl acetate/methanol 1:1) to obtain **SI-3** (1.56 g, 6.00 mmol, 67 % over two steps) as a white solid. **TLC** (ethyl acetate:methanol, 1:1 v/v): $R_f = 0.5$. **LC-MS (ESI)**: $t_R = 4.92$ min; *m/z* = 261.00 $[M + H]^+$. **^{1}H NMR** (400 MHz, $CDCl_3$): δ = 10.93 (s, 1H), 7.65 – 7.59 (m, 1H), 7.55 – 7.51 (m 1H), 7.12 – 7.06 (m, 1H), 6.97 – 6.92 (m, 1H), 4.72 (d, J = 2.6 Hz, 2H), 3.78 (dd, *J* = 11.4, 2.9 Hz, 6H). **^{13}C NMR** (101 MHz, $CDCl_3$): δ = 169.86, 160.32, 135.53, 134.05, 122.67, 114.19, 67.73, 53.84. **HRMS (ESI)**: *m/z* = 261.0523 calcd. for $[C_{10}H_{13}O_6P + H]^+$; found: 261.0514.

Synthesis of 2-((phenylcarbamoyl)methoxy)phenylphosphonic acid (**11**)

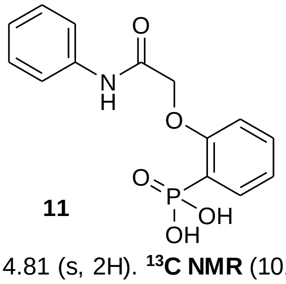

11

The phenylphosphonic acid derivative **11** was synthesized via general method C and general method F. General method C employed the acid **SI-3** (0.130 g, 0.500 mmol), EDC (0.383 g, 2.000 mmol), HOBt (0.270 g, 2.000 mmol), DIPEA (0.51 mL, 3.000 mmol) and aniline (91 µL, 1.000 mmol) in DCM (25 mL). The crude intermediate was deprotected via general method F using TMSBr (0.33 mL, 2.500 mmol) in dry DCM (3 mL) and MeOH/H_2O (3:1, 3 mL). The crude product was purified by HPLC to obtain **11** (9.9 mg, 0.032 mmol, 6 % over two steps) as a white solid. **LC-MS (ESI)**: $t_R = 4.86$ min; *m/z* = 308.04 $[M + H]^+$. **^{1}H NMR** (400 MHz, DMSO): δ = 10.95 (s, 1H), 7.84 (d, *J* = 8.0 Hz, 2H), 7.66 – 7.60 (m, 1H), 7.54 (t, *J* = 7.6 Hz, 1H), 7.32 (t, *J* = 7.3 Hz, 2H), 7.20 (t, *J* = 7.4 Hz, 1H), 7.11 – 7.06 (m, 2H), 4.81 (s, 2H). **^{13}C NMR** (101 MHz, DMSO): δ = 166.39, 158.40, 138.40, 132.81, 132.01, 128.20, 123.17, 120.96, 119.10, 112.78, 76.05, 67.44. **HRMS (ESI)**: *m/z* = 308.0682 calcd. for $[C_{14}H_{14}NO_5P + H]^+$; found: 308.0669.

Synthesis of 2-((benzylcarbamoyl)methoxy)phenylphosphonic acid (**12**)

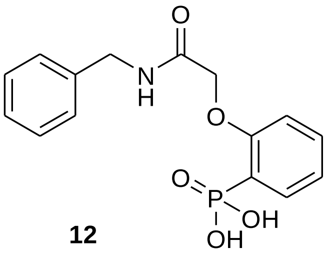

12

The phenylphosphonic acid derivative **12** was synthesized via general method C and general method F. General method C employed the acid **SI-3** (0.130 g, 0.500 mmol), EDC (0.383 g, 2.000 mmol), HOBt (0.270 g, 2.000 mmol), DIPEA (0.51 mL, 3.000 mmol) and benzylamine (109 µL, 1.000 mmol) in DCM (25 mL). The crude intermediate was deprotected via general method F using TMSBr (0.33 mL, 2.500 mmol) in dry DCM (3 mL) and MeOH/H_2O (3:1, 3 mL). The crude product was purified by HPLC to obtain **12** (14.3 mg, 0.044 mmol, 9 % over two steps) as a white solid. **LC-MS (ESI)**: t_R = 4.76 min; *m/z* = 322.07 $[M + H]^+$. **^{1}H NMR** (400 MHz, DMSO): δ = 9.63 (s, 1H), 7.64 – 7.58 (m, 1H), 7.51 (t, *J* = 7.8 Hz, 1H), 7.28 – 7.15 (m, 6H), 7.09 – 7.05 (m, 1H), 4.71 (s, 2H), 4.34 (t, *J* = 5.9 Hz, 2H). **^{13}C NMR** (101 MHz, DMSO): δ = 168.26, 159.14, 139.41, 133.34, 132.76, 132.70, 128.37, 127.31, 126.85, 123.36, 121.43, 113.57, 68.67, 41.83. **HRMS (ESI)**: *m/z* = 322.0839 calcd. for $[C_{15}H_{16}NO_5P + H]^+$; found: 322.0828.

Synthesis of 2-((phenethylcarbamoyl)methoxy)phenylphosphonic acid (**3**)

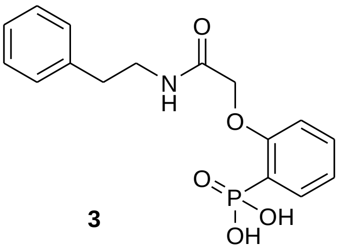

3

The phenylphosphonic acid derivative **3** was synthesized via general method C and general method F. General method C employed the acid **SI-3** (0.130 g, 0.500 mmol), EDC (0.383 g, 2.000 mmol), HOBt (0.270 g, 2.000 mmol), DIPEA (0.51 mL, 3.000 mmol) and phenethylamine (126 µL, 1.000 mmol) in DCM (25 mL). The crude intermediate was deprotected via general method F using TMSBr (0.33 mL, 2.500 mmol) in dry DCM (3 mL) and MeOH/H_2O (3:1, 3 mL). The crude product was purified by HPLC to obtain **3** (7.8 mg, 0.023 mmol, 5 % over two steps) as a white solid. **LC-MS (ESI)**: t_R = 5.17 min; *m/z* = 336.12 $[M + H]^+$. **^{1}H NMR** (400 MHz, DMSO): δ = 9.38 (s, 1H), 7.67 – 7.62 (m, 1H), 7.44 (t, *J* = 7.7 Hz, 1H), 7.26 – 7-16 (m, 5H), 7.09 (t, *J* = 6.9 Hz, 1H), 7.01 (t, *J* = 5.5 Hz, 1H), 4.56 (s, 2H), 3.33 – 3.29 (m, 2H), 2.73 (t, *J* = 7.5 Hz, 2H). **^{13}C NMR** (101 MHz, DMSO): δ = 167.81, 158.84, 128.63, 128.59, 128.53, 128.21, 127.55, 125.95, 114.10, 77.47, 67.64, 34.90. **HRMS (ESI)**: *m/z* = 336.0995 calcd. for $[C_{16}H_{18}NO_5P + H]^+$; found: 336.0982.

Synthesis of 2-((3-phenylpropylcarbamoyl)methoxy)phenylphosphonic acid (**13**)

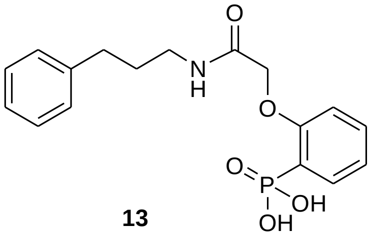

13

The phenylphosphonic acid derivative **13** was synthesized via general method C and general method F. General method C employed the acid **SI-3** (0.130 g, 0.500 mmol), EDC (0.383 g, 2.000 mmol), HOBt (0.270 g, 2.000 mmol), DIPEA (0.51 mL, 3.000 mmol) and 3-phenylpropylamine (142 µL, 1.00 mmol) in DCM (25 mL). The crude intermediate was deprotected via general method F using TMSBr (0.33 mL, 2.500 mmol) in dry DCM (3 mL) and MeOH/H_2O (3:1, 3 mL). The crude product was purified by HPLC to obtain **13** (12.9 mg, 0.037 mmol, 7 % over two steps) as a white solid. **LC-MS (ESI)**: t_R = 5.52 min; *m/z* = 350.16 $[M + H]^+$. **^{1}H NMR** (400 MHz, DMSO): δ = 9.00 (s, 1H), 7.65 – 7.60 (m, 1H), 7.51 (t, *J* = 7.8 Hz, 1H), 7.26 (t, *J* = 7.4 Hz, 1H), 7.18 – 7.14 (m, 4H), 7.08 – 7.05 (m, 1H), 7.01 (t, *J* = 5.5 Hz, 1H), 4.64 (s, 2H), 3.16 – 3.11 (m, 2H), 2.56 – 2.54 (m 2H), 1.76 – 1.69 (m, 2H). **^{13}C NMR** (101 MHz, DMSO): δ = 168.15, 159.49, 142.19, 133.54, 132.91, 128.78, 128.62, 126.08, 123.45, 121.42, 113.50, 68.01, 38.38, 32.86, 31.1. **HRMS (ESI)**: *m/z* = 350.1152 calcd. for $[C_{17}H_{20}NO_5P + H]^+$; found: 350.1135.

Synthesis of the derivatives 14 - 17

Synthesis of 3-(2-(methoxyphosphono)phenoxy)propanoic acid (**SI-4**)

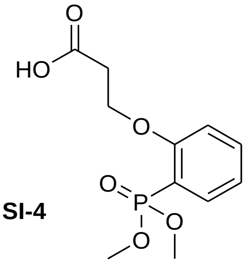

SI-4

The phosphonate **SI-2** (1.011 g, 5.00 mmol, 1.0 eq) was dissolved in dry THF (50 mL) and potassium *tert*-butoxide (0.673 g, 6.00 mmol, 1.2 eq) was added. After 20 min *beta*-propiolactone (0.35 mL, 5.50 mmol, 1.1 eq) was added and the suspension was stirred for 16 h at room temperature. The reaction mixture was acidified with 1M HCl solution (to a pH < 3) and the solvent was evaporated under reduced pressure. The crude product was purified by column chromatography (cyclohexane/ethyl acetate/methanol 2:1:0 → 0:2:1) to obtain **SI-4** (1.16 g, 4.23 mmol, 85 %) as a white solid. **TLC** (ethyl acetate:methanol, 2:1 v/v): R_f = 0.4. **LC-MS (ESI)**: t_R = 5.14 min; *m/z* = 274.90 $[M + H]^+$. **^{1}H NMR** (400 MHz, $CDCl_3$): δ = 10.55 (s, 1H), 7.76-7.70 (m, 1H), 7.50 – 7.46 (m, 1H), 6.99 6.91 (m, 2H), 4.29 (d, *J* = 6.0 Hz, 2H), 3.74 – 3.70 (m, 6H), 2.81 (d, *J* = 6.0 Hz, 2H). **^{13}C NMR** (101 MHz, $CDCl_3$): δ = 173.44, 160.39, 134.92, 120.91, 115.70, 112.26, 64.42, 53.08, 34.30. **HRMS (ESI)**: *m/z* = 275.0679 calcd. for $[C_{11}H_{15}O_6P + H]^+$; found: 275.067.

Synthesis of 2-(2-(phenylcarbamoyl)ethoxy)phenylphosphonic acid (**14**)

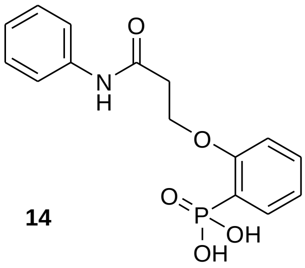

14

The phenylphosphonic acid derivative **14** was synthesized via general method C and general method F. General method C employed the acid **SI-4** (0.137 g, 0.500 mmol), EDC (0.383 g, 2.000 mmol), HOBt (0.270 g, 2.000 mmol), DIPEA (0.51 mL, 3.000 mmol) and aniline (91 µL, 1.000 mmol) in DCM (25 mL). The crude intermediate was deprotected via general method F using TMSBr (0.33 mL, 2.500 mmol) in dry DCM (3 mL) and MeOH/H_2O (3:1, 3 mL). The crude product was purified by HPLC to obtain **14** (8.4 mg, 0.026 mmol, 5 % over two steps) as a white solid. **LC-MS (ESI)**: t_R = 5.21 min; *m/z* = 322.05 [M + H]$^+$. **^{1}H NMR** (400 MHz, DMSO): δ = 10.05 (s, 1H), 7.67 – 7.62 (m, 3H), 7.48 (t, *J* = 7.1 Hz, 1H), 7.28 (t, *J* = 7.7 Hz, 2H), 7.14 – 6.99 (m, 3H), 4.33 (t, *J* = 6.2 Hz, 2H), 2.85 – 2.81 (m, 2H). **^{13}C NMR** (101 MHz, DMSO): δ = 173.35, 159.88, 133.02, 132.68, 131.22, 128.67, 128.60, 119.56, 118.03, 108.24, 64.90. **HRMS (ESI)**: *m/z* = 322.0839 calcd. for [$C_{15}H_{16}NO_5P$ + H]$^+$; found: 322.0828.

Synthesis of 2-(2-(benzylcarbamoyl)ethoxy)phenylphosphonic acid (**15**)

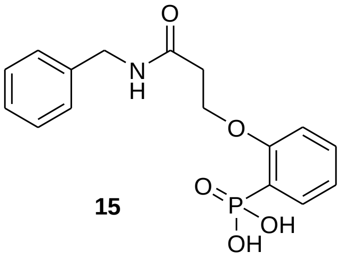

15

The phenylphosphonic acid derivative **15** was synthesized via general method C and general method F. General method C employed the acid **SI-4** (0.137 g, 0.500 mmol), EDC (0.383 g, 2.000 mmol), HOBt (0.270 g, 2.000 mmol), DIPEA (0.51 mL, 3.000 mmol) and benzylamine (109 µL, 1.000 mmol) in DCM (25 mL). The crude intermediate was deprotected via general method F using TMSBr (0.33 mL, 2.500 mmol) in dry DCM (3 mL) and MeOH/H_2O (3:1, 3 mL). The crude product was purified by HPLC to obtain **15** (9.2 mg, 0.027 mmol, 5 % over two steps) as a white solid. **LC-MS (ESI)**: t_R = 5.22 min; *m/z* = 336.06 [M + H]$^+$. **^{1}H NMR** (400 MHz, DMSO): δ = 8.67 (s, 1H), 7.67 – 7.61 (m, 1H), 7.48 (t, *J* = 7.9 Hz, 1H), 7.30 – 7.21 (m, 5H), 7.09 (t, *J* = 7.0 Hz, 1H), 7.02 – 6.99 (m, 1H), 4.29 – 4.24 (m, 4H), 2.68 (t, *J* = 6.2 Hz, 2H). **^{13}C NMR** (101 MHz, DMSO): δ = 170.49, 159.34, 139.22, 133.02, 128.21, 127.25, 126.69, 120.20, 112.70, 65.37, 41.85, 35.69. **HRMS (ESI)**: *m/z* = 336.0995 calcd. for [$C_{16}H_{18}NO_5P$ + H]$^+$; found: 336.0986.

Synthesis of 2-(2-(phenethylcarbamoyl)ethyloxy)phenylphosphonic acid (**16**)

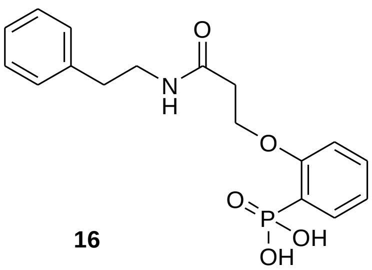

16

The phenylphosphonic acid derivative **16** was synthesized via general method C and general method F. General method C employed the acid **SI-4** (0.137 g, 0.500 mmol), EDC (0.383 g, 2.000 mmol), HOBt (0.270 g, 2.000 mmol), DIPEA (0.51 mL, 3.000 mmol) and phenethylamine (126 µL, 1.00 mmol) in DCM (25 mL). The crude intermediate was deprotected via general method F using TMSBr (0.33 mL, 2.500 mmol) in dry DCM (3 mL) and MeOH/H_2O (3:1, 3 mL). The crude product was purified by HPLC to obtain **16** (8.9 mg, 0.025 mmol, 5 % over two steps) as a white solid. **LC-MS (ESI)**: t_R = 5.54 min; *m/z* = 350.01 [M + H]$^+$. **^{1}H NMR** (400 MHz, DMSO): δ = 8.24 (s, 1H), 7.67 – 7.62 (m, 1H), 7.48 (t, *J* = 7.8 Hz, 1H), 7.26 – 7.22 (m, 2H), 7.18 – 7.17 (m, 3H), 7.08 – 6.99 (m, 2H), 4.18 (t, *J* = 6.1 Hz, 2H), 3.31 - 3.26 (m, 2H), 2.72 (t, *J* = 7.5 Hz, 2H), 2.58 (t, *J* = 6.1 Hz, 2H). **^{13}C NMR** (101 MHz, DMSO): δ = 170.36, 159.37, 139.48, 132.93, 128.61, 128.24, 126.00, 120.12, 112.45, 64.91, 40.30, 35.62, 34.97. **HRMS (ESI)**: *m/z* = 350.1152 calcd. for $C_{17}H_{20}NO_5P$ + H]$^+$; found: 350.1145.

Synthesis of 2-(2-(3-phenylpropylcarbamoyl)ethoxy)phenylphosphonic acid (**17**)

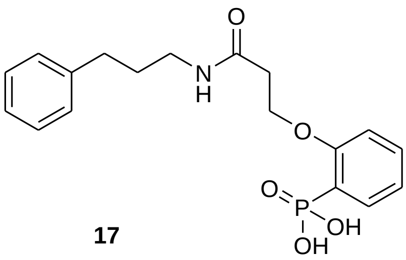

17

The phenylphosphonic acid derivative **17** was synthesized via general method C and general method F. General method C employed the acid **SI-4** (0.137 g, 0.500 mmol), EDC (0.383 mg, 2.000 mmol), HOBt (0.270 g, 2.000 mmol), DIPEA (0.51 mL, 3.000 mmol) and phenethylamine (126 µL, 1.000 mmol) in DCM (25 mL). The crude intermediate was deprotected via general method F using TMSBr (0.33 mL, 2.500 mmol) in dry DCM (3 mL) and MeOH/H_2O (3:1, 3 mL). The crude product was purified by HPLC to obtain **17** (9.3 mg, 0.026 mmol, 5 % over two steps) as a white solid. **LC-MS (ESI)**: t_R = 5.94 min; *m/z* = 364.11 [M + H]$^+$. **^{1}H NMR** (400 MHz, DMSO): δ = 8.18 (s, 1H), 7.66 – 7.60 (m, 1H), 7.47 (t, *J* = 7.9 Hz, 1H), 7.25 (t, *J* = 7.8 Hz, 2H), 7.17 (d, *J* = 7.2 Hz, 3H), 7.07 (t, *J* = 7.2 Hz, 1H), 6.01 – 6.98 (m, 1H), 4.22 (t, *J* = 6.1 Hz, 2H), 3.10 – 3.06 (m, 2H), 2.62 – 2.57 (m, 2H), 1.70 (t, *J* = 7.3 Hz, 2H). **^{13}C NMR** (101 MHz, DMSO): δ = 170.36 159.38, 141.90, 132.86, 128.38, 125.63, 120.28, 112.93, 65.09, 38.34, 35.61, 32.58, 30.78. **HRMS (ESI)**: *m/z* = 364.1308 calcd. for [$C_{18}H_{22}NO_5P$ + H]$^+$; found: 364.1291.

Synthesis of the derivatives 18 - 23

Synthesis of 2-bromo-N-phenethylacetamide (**SI-5**)

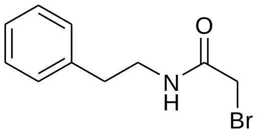

SI-5

The bromoacetamide **SI-5** was synthesized via general method D using phenethylamine (0.63 mL, 5.00 mmol), triethylamine (0.76 mL, 5.50 mmol) and bromoacatyl bromide (0.44 mL, 5.00 mmol), in dry DCM (25 mL). The crude product was purified by HPLC to obtain **SI-5** (0.96 g, 3.99 mmol, 80 %) as a white solid. **LC-MS (ESI)**: t_R = 7.75 min; *m/z* = 242.05 $[M + H]^+$. **^{1}H NMR** (400 MHz, $CDCl_3$): δ = 7.28 - 7.24 (m, 2H), 7.20 – 7.14 (m, 3H), 6.89 (s, 1H), 3.73 (s, 2H), 3.49 – 3.44 (m, 2H), 2.79 (t, *J* = 7.0 Hz, 2H). **^{13}C NMR** (101 MHz, $CDCl_3$): δ = 165.79, 138.36, 128.66, 128.56, 126.53, 41.29, 35.24, 29.02. **HRMS (ESI)**: *m/z* = 242.0175 calcd. for $[C_{10}H_{12}BrNO + H]^+$; found: 242.017.

Synthesis of dimethyl o-tolyl phosphate (**SI-6**)

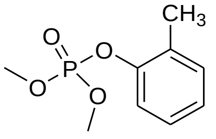

SI-6

The phosphate **SI-6** was synthesized via general method A using *o*-cresol (2.08 mL, 20.00 mmol), tetrachlormethane (1.92 mL, 20.00 mmol), triethylamine (2.98 mL, 20.00 mmol) and dimethyl phosphite (2.77 mL, 30.00 mmol) in dry DCM (15 mL). The crude product was purified by column chromatography (cyclohexane/ethyl acetate 2:1 → 1:1) to obtain **SI-6** (1.30 g, 6.03 mmol, 30 %) as a colorless oil. **TLC** (cyclohexane:ethyl acetate, 1:1 v/v): R_f = 0.3. **LC-MS (ESI)**: t_R = 7.76 min; *m/z* = 217.07 $[M + H]^+$. **^{1}H NMR** (400 MHz, $CDCl_3$): δ = 7.37 - 7.26 (m, 3H), 7.20 (t, *J* = 7.4 Hz, 1H), 3.99 (d, *J* = 11.5 Hz, 6H), 2.43 (s, 3H). **^{13}C NMR** (400 MHz, $CDCl_3$): δ = 148.91, 131.52, 129.23, 127.19, 125.36, 119.60, 55.19, 16.25. **HRMS (ESI)**: *m/z* = 217.0624 calcd. for $[C_9H_{13}O_4P + H]^+$; found: 217.0622.

Synthesis of dimethyl 2-hydroxy-3-methylphenylphosphonate (**SI-7**)

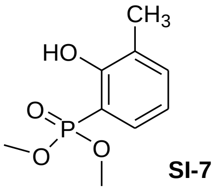

SI-7

The phosphonate **SI-7** was synthesized via general method B using di-*iso*propyl amine (1.42 mL, 10.17 mmol), *n*-BuLi (2.5 M, 3.59 mL, 9.07 mmol) and phosphate **SI-6** (1.31 g, 6.03 mmol) in dry THF (30 mL + 4 mL). The crude product was purified by column chromatography (cyclohexane/ethyl acetate 2:1) to obtain **SI-7** (0.45 g, 2.08 mmol, 34 %) as a yellow oil. **TLC** (cyclohexane:ethyl acetate, 2:1 v/v): R_f = 0.5. **LC-MS (ESI)**: t_R = 7.77 min; *m/z* = 217.03 $[M + H]^+$, calcd. for $C_9H_{13}O_4P$: 216.06. **^{1}H NMR** (400 MHz, $CDCl_3$): δ = 10.02 (s, 1H), 7.23 – 7.19 (m, 1H), 6.89 – 6.74 (m, 2H), 3.74 (d, *J* = 11.4 Hz, 6H), 2.34 (s, 3H). **^{13}C NMR** (101 MHz, $CDCl_3$): δ = 157.59, 148.52, 131.36, 120.94, 118.06, 52.87, 21.78.

Synthesis of 2-((phenethylcarbamoyl)methoxy)-3-methylphenylphosphonic acid (**18**)

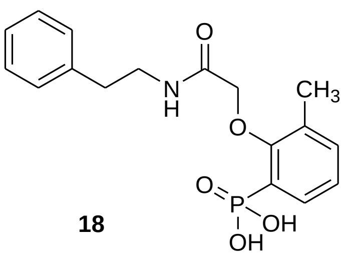

18

The phenylphosphonic acid derivative **18** was synthesized via general method E and general method F. General method E employed phosphonate **SI-7** (93 mg, 0.43 mmol), potassium carbonate (0.12 g, 0.86 mmol) and **SI-5** (116 mg, 0.48 mmol) in acetone (11 mL). The crude intermediate was deprotected via general method F using TMSBr (0.28 mL, 2.15 mmol) in dry DCM (2.5 mL) and MeOH/H_2O (3:1, 2.4 mL). The crude product was purified by HPLC to obtain **18** (38 mg, 0.11 mmol, 25 % over two steps) as a white solid. **LC-MS (ESI)**: t_R = 6.50 min; *m/z* = 350.08 $[M + H]^+$. **^{1}H NMR** (400 MHz, DMSO): δ = 8.63 (s, 1H), 7.53 – 7.48 (m, 1H), 7.38 (d, *J* = 7.4 Hz, 1H), 7.31 – 7.24 (m, 4H), 7.22 – 7.18 (m, 1H), 7.13 – 7.07 (m, 1H), 4.40 (s, 2H), 3.42 – 3.38 (m, 2H), 2.80 (t, *J* = 7.5 Hz, 2H), 2.25 (s, 3H). **^{13}C NMR** (101 MHz, DMSO): δ = 168.28, 143.33, 139.30, 130.58, 128.59, 128.32, 126.09, 71.66, 34.97, 33.72, 16.24. **HRMS (ESI)**: *m/z* = 350.1152 calcd. for $[C_{17}H_{20}NO_5P + H]^+$; found: 350.1135.

Synthesis of dimethyl m*-tolyl phosphate* (**SI-8**)

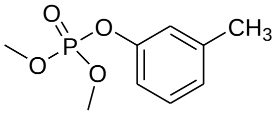

SI-8

The phosphate **SI-8** was synthesized via general method A using *m*-cresol (2.10 mL, 20.00 mmol), tetrachlormethane (1.92 mL, 20.00 mmol), triethylamine (2.98 mL, 20.0 mmol) and dimethyl phosphite (2.77 mL, 30.00 mmol) in dry DCM (15 mL). The crude product was purified by column chromatography (cyclohexane/ethyl acetate 2:1 → 1:1) to obtain **SI-8** (1.90 g, 8.79 mmol, 44 %) as a colorless oil. **TLC** (cyclohexane:ethyl acetate, 1:1 v/v): R_f = 0.4. **LC-MS (ESI)**: t_R = 7.47 min; *m/z* = 216.99 $[M + H]^+$. **^{1}H NMR** (400 MHz, $CDCl_3$): δ = 7.16 - 7.11 (m, 1H), 6.97 - 6.90 (m, 3H), 3.99 (d, *J* = 11.5 Hz, 6H), 2.27 (s, 3H). **^{13}C NMR** (101 MHz, $CDCl_3$): δ = 150.43, 139.88, 129.29, 125.78, 120.24, 116.60, 54.65, 21.11. **HRMS (ESI)**: *m/z* = 217.0624 calcd. for $[C_9H_{13}O_4P + H]^+$; found: 217.0621.

Synthesis of dimethyl 2-hydroxy-4-methylphenylphosphonate (**SI-9**)

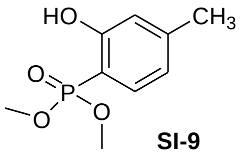

SI-9

The phosphonate **SI-9** was synthesized via general method B using di-*iso*propyl amine (2.07 mL, 14.82 mmol), *n*-BuLi (2.5 M, 5.23 mL, 13.22 mmol) and phosphate **SI-8** (1.91 g, 8.79 mmol) in dry THF (45 mL + 5 mL). The crude product was purified by column chromatography (cyclohexane/ethyl acetate 2:1) to obtain **SI-9** (0.59 g, 2.71 mmol, 31 %) as a white solid. **TLC** (cyclohexane:ethyl acetate, 2:1 v/v): R_f = 0.3. **LC-MS (ESI)**: t_R = 6.84 min; *m/z* = 216.97 $[M + H]^+$. **^{1}H NMR** (400 MHz, $CDCl_3$): δ = 9.90 (s, 1H), 7.26 – 7.21 (m, 1H), 6.81 – 6.75 (m, 2H), 3.73 (d, *J* = 11.5 Hz, 6H), 2.34 (s, 3H). **^{13}C NMR** (101 MHz, $CDCl_3$): δ = 162.39, 146.67, 131.35, 121.10, 117.90, 103.06, 52.87, 21.75. **HRMS (ESI)**: *m/z* = 217.0624 calcd. for $[C_9H_{13}O_4P + H]^+$; found: 217.0621.

Synthesis of 2-((phenethylcarbamoyl)methoxy)-4-methylphenylphosphonic acid (**19**)

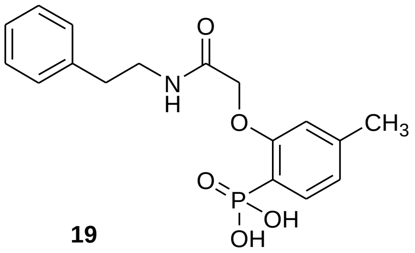

19

The phenylphosphonic acid derivative **19** was synthesized via general method E and general method F. General method E employed phosphonate **SI-9** (82 mg, 0.38 mmol), potassium carbonate (0.11 g, 0.76 mmol) and **SI-5** (116 mg, 0.42 mmol) in acetone (10 mL). The crude intermediate was deprotected via general method F using TMSBr (0.25 mL, 1.90 mmol) in dry DCM (2 mL) and MeOH/H_2O (3:1, 2 mL). The crude product was purified by HPLC to obtain **19** (50 mg, 0.14 mmol, 38 % over two steps) as a white solid. **LC-MS (ESI)**: t_R = 5.58 min; *m/z* = 350.01 $[M + H]^+$. **^{1}H NMR** (400 MHz, DMSO): δ = 9.20 (s, 1H), 7.53 – 7.48 (m, 1H), 7.25 – 7.22 (m, 2H), 7.18 – 7.16 (m, 3H), 6.99 (d, *J* = 5.8 Hz, 1H), 6.89 (d, *J* = 7.7 Hz, 1H), 7.13 – 7.07 (m, 1H), 4.59 (s, 2H), 3.39 – 3.29 (m, 2H), 2.74 (t, *J* = 8.6 Hz, 2H), 2.33 (s, 3H). **^{13}C NMR** (101 MHz, DMSO): δ = 167.94, 158.97, 143.58, 139.34, 132.57, 128.61, 128.33, 126.11, 121.88, 120.00, 118.18, 113.84, 67.59, 34.98, 21.32. **HRMS (ESI)**: *m/z* = 350.1152 calcd. for $[C_{17}H_{20}NO_5P + H]^+$; found: 350.1145.

Synthesis of dimethyl p*-tolyl phosphate* (**SI-10**)

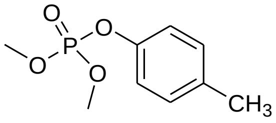

SI-10

The phosphate **SI-10** was synthesized via general method A using *p*-cresol (2.10 mL, 20.00 mmol), tetrachlormethane (1.92 mL, 20.00 mmol), triethylamine (2.98 mL, 20.00 mmol) and dimethyl phosphite (2.77 mL, 30.00 mmol) in dry DCM (15 mL). The crude product was purified by column chromatography (cyclohexane/ethyl acetate 2:1 → 1:1) to obtain **SI-10** (2.23 g, 10.32 mmol, 52 %) as a colorless oil. **TLC** (cyclohexane:ethyl acetate, 1:1 v/v): R_f = 0.3. **LC-MS (ESI)**: t_R = 7.80 min; *m/z* = 217.07 $[M + H]^+$. **^{1}H NMR** (400 MHz, $CDCl_3$): δ = 7.09 - 7.03 (m, 4H), 3.78 (d, *J* = 11.3 Hz, 6H), 2.26 (s, 3H). **^{13}C NMR** (101 MHz, $CDCl_3$): δ = 148.37, 134.70, 130.14, 119.49, 54.81, 20.60. **HRMS (ESI)**: *m/z* = 217.0624 calcd. for $[C_9H_{13}O_4P + H]^+$; found: 217.0623.

Synthesis of dimethyl 2-hydroxy-5-methylphenylphosphonate (**SI-11**)

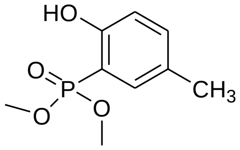

SI-11

The phosphonate **SI-11** was synthesized via general method B using di-*iso*propyl amine (2.43 mL, 17.38 mmol), *n*-BuLi (2.5 M, 6.13 mL, 15.51 mmol) and phosphate **SI-10** (2.24 g, 10.31 mmol) in dry THF (50 mL + 5 mL). The crude product was purified by column chromatography (cyclohexane/ethyl acetate 2:1) to obtain **SI-10** (1.48 g, 6.83 mmol, 66 %) as a white solid. **TLC** (cyclohexane:ethyl acetate, 2:1 v/v): R_f = 0.3. **LC-MS (ESI)**: t_R = 6.84 min; *m/z* = 216.97 $[M + H]^+$. **^{1}H NMR** (400 MHz, $CDCl_3$): δ = 9.55 (s, 1H), 7.21 (d, *J* = 8.5 Hz, 1H), 7.17 – 7.07 (m, 1H), 6.89 – 6.79 (m, 1H), 3.71 (d, *J* = 11.6 Hz, 6H), 2.23 (s, 3H). **^{13}C NMR** (101 MHz, $CDCl_3$): δ = 159.88, 136.62, 131.31, 129.18, 117.60, 107.53, 53.12, 20.19. **HRMS (ESI)**: *m/z* = 217.0624 calcd. for $[C_9H_{13}O_4P + H]^+$; found: 217.0621.

Synthesis of 2-((phenethylcarbamoyl)methoxy)-5-methylphenylphosphonic acid (**20**)

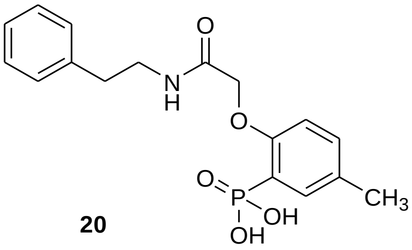

20

The phenylphosphonic acid derivative **20** was synthesized via general method E and general method F. General method E employed phosphonate **SI-10** (151 mg, 0.70 mmol), potassium carbonate (0.19 g, 1.40 mmol) and **SI-5** (186 mg, 0.77 mmol) in acetone (18 mL). The crude intermediate was deprotected via general method F using TMSBr (0.46 mL, 3.50 mmol) in dry DCM (4.2 mL) and MeOH/H_2O (3:1, 4 mL). The crude product was purified by HPLC to obtain **20** (24 mg, 0.07 mmol, 10 % over two steps) as a white solid. **LC-MS (ESI)**: t_R = 6.21 min; *m/z* = 350.10 $[M + H]^+$. **^{1}H NMR** (400 MHz, DMSO): δ = 9.18 (s, 1H), 7.40 (d, *J* = 14.5 Hz, 1H), 7.30 – 7.18 (m, 3H), 7.21 – 7.16 (m, 3H), 7.04 – 7.00 (m, 1H), 4.56 (s, 2H), 3.35 – 3.31 (m, 2H), 2.73 (t, *J* = 7.6 Hz, 2H), 2.27 (s, 3H). **^{13}C NMR** (101 MHz, DMSO): δ = 167.98, 156.94, 139.32, 133.36, 128.58, 128.30, 126.07, 122.83, 121.05, 113.20, 67.83, 40.06, 34.94, 20.10. **HRMS (ESI)**: *m/z* = 350.1152 calcd. for $[C_{17}H_{20}NO_5P + H]^+$; found: 350.1137.

Synthesis of 2-fluorophenyl dimethyl phosphate (**SI-11**)

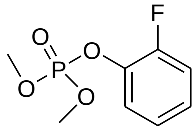

SI-11

The phosphate **SI-11** was synthesized via general method A using *o*-fluorophenol (1.79 mL, 20.00 mmol), tetrachlormethane (1.92 mL, 20.00 mmol), triethylamine (2.98 mL, 20.00 mmol) and dimethyl phosphite (2.77 mL, 30.00 mmol) in dry DCM (15 mL). The crude product was purified by column chromatography (cyclohexane/ethyl acetate 2:1 → 1:1) to obtain **SI-11** (1.75 g, 7.96 mmol, 40 %) as a colorless oil. **TLC** (cyclohexane:ethyl acetate, 1:1 v/v): R_f = 0.4. **LC-MS (ESI)**: t_R = 7.40 min; *m/z* = 221.04 $[M + H]^+$. **^{1}H NMR** (400 MHz, $CDCl_3$): δ = 7.34 - 7.30 (m, 1H), 7.11. - 7.06 (m, 3H), 3.88 - 3.84 (m, 6H). **^{13}C NMR** (101 MHz, $CDCl_3$): δ = 154.27, 152.56, 138.44, 126.20, 124.67, 122.39, 116.92, 55.18. **HRMS (ESI)**: *m/z* = 221.0373 calcd. for $[C_8H_{10}FO_4P + H]^+$; found: 221.0371.

Synthesis of dimethyl 3-fluoro-2-hydroxyphenylphosphonate (**SI-12**)

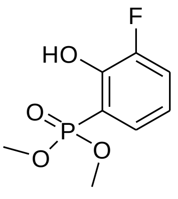

SI-12

The phosphonate **SI-12** was synthesized via general method B using di-*iso*propyl amine (1.88 mL, 13.42 mmol), *n*-BuLi (2.5 M, 4.73 mL, 11.97 mmol) and phosphate **SI-11** (1.75 g, 7.96 mmol) in dry THF (35 mL + 5 mL). The crude product was purified by column chromatography (cyclohexane/ethyl acetate 2:1) to obtain **SI-12** (0.34 g, 1.55 mmol, 19 %) as a slightly yellow solid. **TLC** (cyclohexane:ethyl acetate, 2:1 v/v): R_f = 0.2. **LC-MS (ESI)**: t_R = 6.23 min; *m/z* = 220.98 $[M + H]^+$. **^{1}H NMR** (400 MHz, $CDCl_3$): δ = 9.27 (s, 1H), 7.21 - 7.16 (m, 1H), 7.11. - 7.05 (m, 1H), 6.84. – 6.78 (m, 1H), 3.72 (d, *J* = 11.6 Hz, 6H). **^{13}C NMR** (101 MHz, $CDCl_3$): δ = 152.98, 150.57, 126.84, 121.56, 119.96, 110.96, 109.20, 53.43. **HRMS (ESI)**: *m/z* = 221.0373 calcd. for $[C_8H_{10}FO_4P + H]^+$; found: 221.0361.

Synthesis of 2-((phenethylcarbamoyl)methoxy)-3-fluorophenylphosphonic acid (**21**)

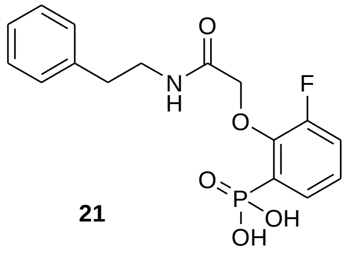

21

The phenylphosphonic acid derivative **21** was synthesized via general method E and general method F. General method E employed phosphonate **SI-12** (97 mg, 0.44 mmol), potassium carbonate (0.12 g, 0.88 mmol) and **SI-5** (116 mg, 0.48 mmol) in acetone (11 mL). The crude intermediate was deprotected via general method F using TMSBr (0.29 mL, 2.20 mmol) in dry DCM (2.6 mL) and MeOH/H_2O (3:1, 2.8 mL). The crude product was purified by HPLC to obtain **21** (63 mg, 0.18 mmol, 40 % over two steps) as a white solid. **LC-MS (ESI)**: t_R = 5.95 min; *m/z* = 354.10 $[M + H]^+$. **^{1}H NMR** (400 MHz, DMSO): δ = 9.27 (s, 1H), 7.49 – 7.44 (m, 2H), 7.29 – 7.18 (m, 6H), 4.56 (d, *J* = 1.9 Hz, 2H), 3.40 – 3.35 (m, 2H), 2.78 (t, *J* = 7.6 Hz, 2H). **^{13}C NMR** (101 MHz, DMSO): δ = 168.02, 139.32, 128.64, 128.41, 126.19, 123.92, 120.58, 72.12, 40.22, 34.98. **HRMS (ESI)**: *m/z* = 354.0901 calcd. for $[C_{16}H_{17}FO_5P + H]^+$; found: 354.0896.

Synthesis of 3-fluorophenyl dimethyl phosphate (**SI-13**)

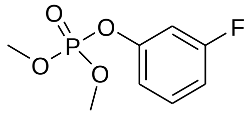

SI-13

The phosphate **SI-13** was synthesized via general method A using *m*-fluorophenol (1.81 mL, 20.00 mmol), tetrachlormethane (1.92 mL, 20.00 mmol), triethylamine (2.98 mL, 20.00 mmol) and dimethyl phosphite (2.77 mL, 30.00 mmol) in dry DCM (15 mL). The crude product was purified by column chromatography (cyclohexane/ethyl acetate 2:1 → 1:1) to obtain **SI-13** (1.65 g, 7.47 mmol, 34 %) as a colorless oil. **TLC** (cyclohexane:ethyl acetate, 1:1 v/v): R_f = 0.4. **LC-MS (ESI)**: t_R = 7.95 min; *m/z* = 220.96 $[M + H]^+$, calcd. for $C_8H_{10}FO_4P$: 220.03. **^{1}H NMR** (400 MHz, $CDCl_3$): δ = = 7.32 - 7.26 (m, 1H), 7.03. – 6.95 (m, 2H), 6.91 – 6.87 (m, 1H), 3.87 - 3.84 (m, 6H). **^{13}C NMR** (101 MHz, $CDCl_3$): δ = 164.23, 161.77, 151.46, 130.53, 115.70, 112.32, 107.88, 54.98.

Synthesis of dimethyl 4-fluoro-2-hydroxyphenylphosphonate (**SI-14**)

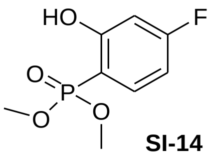

SI-14

The phosphonate **SI-14** was synthesized via general method B using di-*iso*propyl amine (1.78 mL, 12.69 mmol), *n*-BuLi (2.5 M, 4.45 mL, 11.21 mmol) and phosphate **SI-13** (1.64 g, 7.47 mmol) in dry THF (30 mL + 5 mL). The crude product was purified by column chromatography (cyclohexane/ethyl acetate 2:1) to obtain **SI-14** (0.37 g, 1.68 mmol, 23 %) as a white solid. **TLC** (cyclohexane:ethyl acetate, 2:1 v/v): R_f = 0.5. **LC-MS (ESI)**: t_R = 7.50 min; *m/z* = 220.98 $[M + H]^+$, calcd. for $C_8H_{10}FO_4P$: 220.03. **^{1}H NMR** (400 MHz, $CDCl_3$): δ = 10.77 (s, 1H), 7.44 - 7.38 (m, 1H), 6.79. – 6.76 (m, 1H), 6.62. – 6.56 (m, 1H), 3.82 – 3.77 (m, 6H). **^{13}C NMR** (101 MHz, $CDCl_3$): δ = 164.15, 162.60, 136.49, 114.08, 106.47, 53.83.

Synthesis of 2-((phenethylcarbamoyl)methoxy)-4-fluorophenylphosphonic acid (**22**)

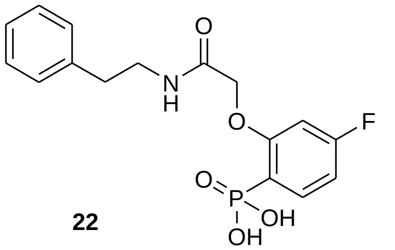

22

The phenylphosphonic acid derivative **22** was synthesized via general method E and general method F. General method E employed phosphonate **SI-14** (79 mg, 0.36 mmol), potassium carbonate (0.10 g, 0.72 mmol) and **SI-5** (97 mg, 0.40 mmol) in acetone (9 mL). The crude intermediate was deprotected via general method F using TMSBr (0.24 mL, 1.80 mmol) in dry DCM (2.2 mL) and MeOH/H_2O (3:1, 2.2 mL). The crude product was purified by HPLC to obtain **22** (22 mg, 0.06 mmol, 17 % over two steps) as a white solid. **LC-MS (ESI)**: t_R = 4.92 min; *m/z* = 354.07 [M + H]$^+$. **^{1}H NMR** (400 MHz, DMSO): δ = 9.40 (s, 1H), 7.53 – 7.47 (m, 2H), 7.28 – 7.16 (m, 5H), 6.94 – 6.83 (m, 2H), 4.66 (s, 2H), 3.38 – 3.33 (m, 2H), 2.79 – 2.75 (m, 2H). **^{13}C NMR** (101 MHz, DMSO): δ = 167.53, 159.82, 139.31, 133.88, 128.60, 128.35, 126.12, 109.28, 67.95, 34.97. **HRMS (ESI)**: *m/z* = 354.0901 calcd. for [$C_{16}H_{17}FO_5P$ + H]$^+$; found: 354.0895.

Synthesis of 4-fluorophenyl dimethyl phosphate (**SI-15**)

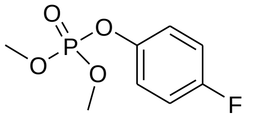

SI-15

The phosphate **SI-15** was synthesized via general method A using *p*-fluorophenol (2.24 g, 20.00 mmol), tetrachlormethane (1.92 mL, 20.00 mmol), triethylamine (2.98 mL, 20.00 mmol) and dimethyl phosphite (2.77 mL, 30.00 mmol) in dry DCM (15 mL). The crude product was purified by column chromatography (cyclohexane/ethyl acetate 2:1 → 1:1) to obtain **SI-15** (2.52 g, 11.46 mmol, 57 %) as a colorless oil. **TLC** (cyclohexane:ethyl acetate, 1:1 v/v): R_f = 0.3. **LC-MS (ESI)**: t_R = 7.39 min; *m/z* = 221.04 [M + H]$^+$. **^{1}H NMR** (400 MHz, $CDCl_3$): δ = 7.19 - 7.16 (m, 2H), 7.04. - 7.00 (m, 2H), 3.87 - 3.84 (m, 6H). **^{13}C NMR** (101 MHz, $CDCl_3$): δ = 161.13, 158.71, 146.66, 121.53, 116.60, 116.36, 55.15. **HRMS (ESI)**: *m/z* = 221.0373 calcd. for [$C_8H_{10}FO_4P$ + H]$^+$; found: 221.0369.

Synthesis of dimethyl 5-fluoro-2-hydroxyphenylphosphonate (**SI-16**)

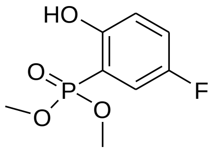

SI-16

The phosphonate **SI-16** was synthesized via general method B using di-*iso*propyl amine (2.74 mL, 19.48 mmol), *n*-BuLi (2.5 M, 6.88 mL, 17.19 mmol) and phosphate **SI-15** (2.31 g, 11.46 mmol) in dry THF (55 mL + 8 mL). The crude product was purified by column chromatography (cyclohexane/ethyl acetate 2:1) to obtain **SI-16** (1.33 g, 6.03 mmol, 53 %) as a white solid. **TLC** (cyclohexane:ethyl acetate, 2:1 v/v): R_f = 0.4. **LC-MS (ESI)**: t_R = 6.40 min; *m/z* = 220.96 [M + H]$^+$. **^{1}H NMR** (400 MHz, $CDCl_3$): δ = 8.83 (bs, 1H), 7.17 - 7.12 (m, 1H), 7.04 - 6.97 (m, 1H), 6.94 - 6.88 (m, 1H), 3.74 (d, *J* = 11.5 Hz, 6H). **^{13}C NMR** (101 MHz, $CDCl_3$): δ = 158.80, 157.66, 154.85, 122.96, 119.50, 116.56, 108.64, 107.08, 53.34. **HRMS (ESI)**: *m/z* = 221.0373 calcd. for [$C_8H_{10}FO_4P$ + H]$^+$; found: 221.068.

Synthesis of 2-((phenethylcarbamoyl)methoxy)-5-fluorophenylphosphonic acid (**23**)

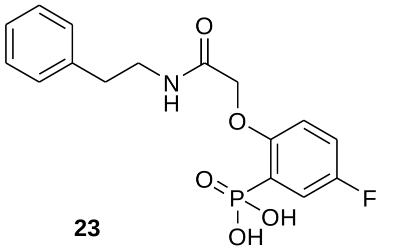

23

The phenylphosphonic acid derivative **23** was synthesized via general method E and general method F. General method E employed phosphonate **SI-16** (115 mg, 0.52 mmol), potassium carbonate (0.14 g, 1.04 mmol) and **SI-5** (138 mg, 0.57 mmol) in acetone (13 mL). The crude intermediate was deprotected via general method F using TMSBr (0.34 mL, 2.60 mmol) in dry DCM (3 mL) and MeOH/H_2O (3:1, 3.2 mL). The crude product was purified by HPLC to obtain **23** (42 mg, 0.12 mmol, 23 % over two steps) as a white solid. **LC-MS (ESI)**: t_R = 5.86 min; *m/z* = 354.07 [M + H]. **^{1}H NMR** (400 MHz, DMSO): δ = 9.07 (s, 1H), 7.35 – 7.30 (m, 2H), 7.24 – 7.15 (m, 5H), 4.59 (s, 2H), 3.36 – 3.31 (m, 2H), 2.73 (t, *J* = 7.6 Hz, 2H). **^{13}C NMR** (101 MHz, DMSO): δ = 167.8, 155.28, 139.24, 128.57, 128.30, 126.02, 115.41, 68.37, 40.05, 34.97. **HRMS (ESI)**: *m/z* = 354.0901 calcd. for [$C_{16}H_{17}FO_5P$ + H]$^+$; found: 354.0892.

Synthesis of the derivatives 24 - 26

Synthesis of N-(2-fluorophenethyl)-2-bromoacetamide (**SI-17**)

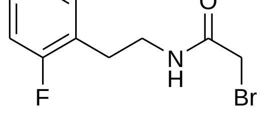

SI-17

The bromoacetamide **SI-17** was synthesized via general method D using 2-(2-fluorophenethyl)amine (0.52 mL, 4.00 mmol), triethylamine (0.61 mL, 4.40 mmol) and bromoacetyl bromide (0.35 mL, 4.00 mmol) in dry DCM (20 mL). The crude product was purified by HPLC to obtain **SI-17** (0.92 g, 3.54 mmol, 88 %) as a white solid. **LC-MS (ESI)**: t_R = 7.92 min; *m/z* = 260.09 [M + H]$^+$. **^{1}H NMR** (400 MHz, $CDCl_3$): δ = 7.24 – 7.17 (m, 1H), 7.10 – 7.01 (m, 3H), 6.69 (s, 1H), 3.82 (s, 2H), 3.55 – 3.51 (m, 2H), 2.88 (t, *J* = 7.0 Hz, 2H). **^{13}C NMR** (101 MHz, $CDCl_3$): δ = 166.25,

162.97, 160.47, 131.18, 128.68, 125.46, 124.35, 115.40, 40.34, 29.15. **HRMS (ESI)**: *m/z* = 260.0081 calcd. for $[C_{10}H_{11}BrFNO + H]^+$; found: 260.0075.

Synthesis of 2-((2-fluorophenethylcarbamoyl)methoxy)phenylphosphonic acid (**24**)

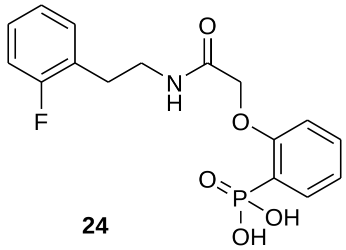

24

The phenylphosphonic acid derivative **24** was synthesized via general method E and general method F. General method E employed phosphonate **SI-2** (133 mg, 0.66 mmol), potassium carbonate (0.18 g, 1.32 mmol) and **SI-17** (190 mg, 0.73 mmol) in acetone (17 mL). The crude intermediate was deprotected via general method F using TMSBr (0.44 mL, 3.30 mmol) in dry DCM (4.0 mL) and MeOH/H_2O (3:1, 4.0 mL). The crude product was purified by HPLC to obtain **24** (124 mg, 0.35 mmol, 53 % over two steps) as a white solid. **LC-MS (ESI)**: t_R = 5.94 min; *m/z* = 353.98 $[M + H]^+$. **^{1}H NMR** (400 MHz, DMSO): δ = 9.25 (s, 1H), 7.65 – 7.60 (m, 1H), 7.51 – 7.48 (m, 1H), 7.25 – 7.21 (m, 2H), 7.15 – 7.02 (m, 4H), 4.61 (s, 2H), 3.37 – 3.32 (m, 2H), 2.78 (t, *J* = 7.6 Hz, 2H). **^{13}C NMR** (101 MHz, DMSO): δ = 167.96, 161.87, 159.34, 133.31, 131.91, 128.81, 125.53, 123.83, 123.28, 121.31, 114.57, 113.44, 67.63, 38.48, 28.33. **HRMS (ESI)**: *m/z* = 354.0901 calcd. for $[C_{16}H_{17}FNO_5P + H]^+$; found: 354.0894.

Synthesis of N-(3-fluorophenethyl)-2-bromoacetamide (**SI-18**)

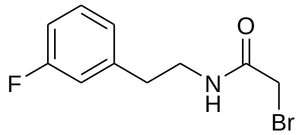

SI-18

The bromoacetamide **SI-18** was synthesized via general method D using 2-(3-fluorophenethyl)amine (0.52 mL, 4.00 mmol), triethylamine (0.61 mL, 4.40 mmol) and bromoacetyl bromide (0.35 mL, 4.00 mmol) in dry DCM (20 mL). The crude product was purified by HPLC to obtain **SI-18** (0.91 g, 3.49 mmol, 87 %) as a white solid. **LC-MS (ESI)**: t_R = 7.93 min; *m/z* = 259.03 $[M + H]^+$. **^{1}H NMR** (400 MHz, $CDCl_3$): δ = 7.24 – 7.21 (m, 1H), 6.94 – 6.86 (m, 3H), 6.46 (s, 1H), 3.80 (s, 2H), 3.52 – 3.47 (m, 2H), 2.80 (t, *J* = 7.0 Hz, 2H). **^{13}C NMR** (101 MHz, $CDCl_3$): δ = 165.53, 141.03, 130.39, 124.54, 115.90, 113.92, 41.22, 35.30, 29.30. **HRMS (ESI)**: *m/z* = 260.0081 calcd. for $[C_{10}H_{11}BrFNO + H]^+$; found: 260.0074.

Synthesis of 2-((3-fluorophenethylcarbamoyl)methoxy)phenylphosphonic acid (**25**)

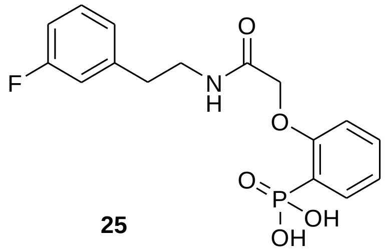

25

The phenylphosphonic acid derivative **25** was synthesized via general method E and general method F. General method E employed phosphonate **SI-2** (148 mg, 0.73 mmol), potassium carbonate (0.20 g, 1.46 mmol) and **SI-18** (209 mg, 0.80 mmol) in acetone (18 mL). The crude intermediate was deprotected via general method F using TMSBr (0.48 mL, 3.65 mmol) in dry DCM (4.4 mL) and MeOH/H_2O (3:1, 4.4 mL). The crude product was purified by HPLC to obtain **25** (180 mg, 0.51 mmol, 70 % over two steps) as a white solid. **LC-MS (ESI)**: t_R = 5.97 min; *m/z* = 354.07 $[M + H]^+$. **^{1}H NMR** (400 MHz, DMSO): δ = 9.18 (s, 1H), 7.65 – 7.60 (m, 1H), 7.51 – 7.48 (m, 1H), 7.28 – 7.23 (m, 1H), 7.15 – 6.97 (m, 5H), 4.60 (s, 2H), 3.39 – 3.34 (m, 2H), 2.78 (t, *J* = 7.4 Hz, 2H). **^{13}C NMR** (101 MHz, DMSO): δ = 167.82, 163.33, 160.92, 158.88, 142.30, 133.13, 132.58, 129.99, 124.74, 121.18, 115.37, 112.72, 67.65, 39.61, 34.43. **HRMS (ESI)**: *m/z* = 354.0901 calcd. for $[C_{16}H_{17}FNO_5P + H]^+$; found: 354.0892.

Synthesis of N-(4-fluorophenethyl)-2-bromoacetamide (**SI-19**)

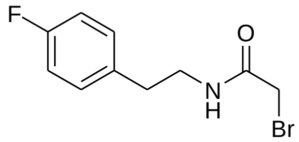

SI-19

The bromoacetamide **SI-19** was synthesized via general method D using 2-(4-fluorophenethyl)amine (0.52 mL, 4.00 mmol), triethylamine (0.61 mL, 4.40 mmol) and bromoacetyl bromide (0.35 mL, 4.00 mmol) in dry DCM (20 mL). The crude product was purified by HPLC to obtain **SI-19** (0.91 g, 3.51 mmol, 88 %) as a white solid. **LC-MS (ESI)**: t_R = 7.90 min; *m/z* = 260.08 $[M + H]^+$. **^{1}H NMR** (400 MHz, $CDCl_3$): δ = 7.15 – 7.12 (m, 2H), 7.00 – 6.96 (m, 2H), 6.52 (s, 1H), 3.81 (s, 2H), 3.51 – 3.47 (m, 2H), 2.79 (t, *J* = 6.7 Hz, 2H). **^{13}C NMR** (101 MHz, $CDCl_3$): δ = 165.52, 163.06, 160.63, 134.07, 130.25, 115.74, 41.49, 34.72, 29.28. **HRMS (ESI)**: *m/z* = 260.0081 calcd. for $[C_{10}H_{11}BrFNO + H]^+$; found: 260.0073.

Synthesis of 2-((4-fluorophenethylcarbamoyl)methoxy)phenylphosphonic acid (**26**)

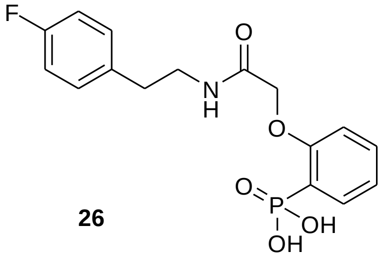

26

The phenylphosphonic acid derivative **26** was synthesized via general methods E and F. General method E employed phosphonate **SI-2** (123 mg, 0.61 mmol), potassium carbonate (0.17 g, 1.22 mmol) and **SI-19** (174 mg, 0.67 mmol) in acetone (15 mL). The crude intermediate was deprotected via general method F using TMSBr (0.4 mL, 3.05 mmol) in dry DCM (4.0 mL) and MeOH/H_2O (3:1, 4.0 mL). The crude product was purified by HPLC to obtain **26** (79 mg, 0.22 mmol, 36 % over two steps) as a white solid. **LC-MS (ESI)**: t_R = 5.93 min; *m/z* = 354.03 [M + H]$^+$. **^{1}H NMR** (400 MHz, DMSO): *δ* = 9.15 (s, 1H), 7.66 – 7.61 (m, 1H), 7.52 – 7.48 (m, 1H), 7.21 – 7.00 (m, 6H), 4.60 (s, 2H), 3.36 – 3.31 (m, 2H), 2.73 (t, *J* = 7.4 Hz, 2H). **^{13}C NMR** (101 MHz, DMSO): *δ* = 168.48, 162.66, 159.86, 135.76, 132.96, 130.43, 121.81, 115.07, 113.94, 67.90, 34.22. **HRMS (ESI)**: *m/z* = 354.0901 calcd. for [$C_{16}H_{17}FNO_5P$ + H]$^+$; found: 354.0891.

Synthesis of the derivatives 27 - 29

Synthesis of N-(2-(trifluoromethyl)phenethyl)-2-bromoacetamide (**SI-20**)

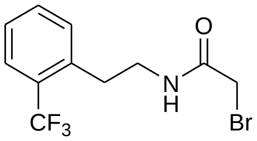

SI-20

The bromoacetamide **SI-20** was synthesized via general method D using 2-(2-trifluoromethyl)phenethylamine (0.8 mL, 5.0 mmol), triethylamine (0.76 mL, 5.5 mmol) and bromoacetyl bromide (0.44 mL, 5.0 mmol) in dry DCM (25 mL). The crude product was purified by HPLC to obtain **SI-20** (1.16 g, 3.73 mmol, 75 %) as a white solid. **LC-MS (ESI)**: t_R = 8.81 min; *m/z* = 310.00 [M + H]$^+$. **^{1}H NMR** (400 MHz, $CDCl_3$): *δ* = 7.63 (t, *J* = 7.9 Hz, 1H), 7.50 (t, *J* = 7.6 Hz, 1H), 7.38 – 7.32 (m, 2H), 6.66 (s, 1H), 3.84 (s, 2H), 3.58 – 3.53 (m, 2H), 3.03 (t, *J* = 7.4 Hz, 2H). **^{13}C NMR** (101 MHz, $CDCl_3$): *δ* = 165.65, 137.05, 131.69, 128.80, 126.28, 123.27, 41.23, 32.28, 29.18. **HRMS (ESI)**: *m/z* = 310.0049 calcd. for [$C_{11}H_{11}BrF_3NO$ + H]$^+$; found: 310.004.

Synthesis of 2-((2-(trifluoromethyl)phenethylcarbamoyl)methoxy)phenylphosphonic acid (**27**)

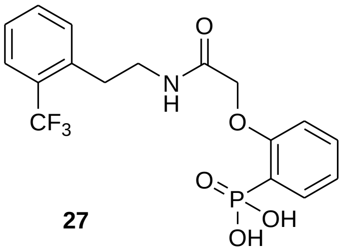

27

The phenylphosphonic acid derivative **27** was synthesized via general methods E and F. General method E employed phosphonate **SI-2** (99 mg, 0.49 mmol), potassium carbonate (0.14 g, 0.98 mmol) and **SI-20** (168 mg, 0.54 mmol) in acetone (12 mL). The crude intermediate was deprotected via general method F using TMSBr (0.32 mL, 2.45 mmol) in dry DCM (3.0 mL) and MeOH/H_2O (3:1, 3.0 mL). The crude product was purified by HPLC to obtain **27** (47 mg, 0.12 mmol, 24 % over two steps) as a white solid. **LC-MS (ESI)**: t_R = 5.59 min; *m/z* = 404.02 [M + H]$^+$. **^{1}H NMR** (400 MHz, DMSO): *δ* = 9.33 (s, 1H), 7.67 – 7.60 (m, 2H), 7.53 – 7.49 (m, 2H), 7.43 – 7.38 (m, 2H), 7.17 – 7.13 (m, 1H), 7.09 – 7.05 (m, 1H), 4.62 (s, 2H), 3.39 – 3.34 (m, 2H), 2.92 (t, *J* = 7.5 Hz, 2H). **^{13}C NMR** (101 MHz, DMSO): *δ* = 167.94, 158.97, 137.56, 132.42, 131.76, 126.83, 125.72, 123.11, 121.08, 113.24, 67.72, 31.82. **HRMS (ESI)**: *m/z* = 404.0869 calcd. for [$C_{17}H_{17}F_3NO_5P$ + H]$^+$; found: 404.0856.

Synthesis of N-(3-(trifluoromethyl)phenethyl)-2-bromoacetamide (**SI-21**)

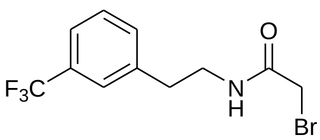

SI-21

The bromoacetamide **SI-21** was synthesized via general method D using 2-(3-trifluoromethyl)phenethylamine (0.80 mL, 5.00 mmol), triethylamine (0.76 mL, 5.50 mmol) and bromoacetyl bromide (0.44 mL, 5.00 mmol) in dry DCM (25 mL). The crude product was purified by HPLC to obtain **SI-21** (1.34 g, 4.33 mmol, 87 %) as a white solid. **LC-MS (ESI)**: t_R = 8.82 min; *m/z* = 310.02 [M + H]$^+$. **^{1}H NMR** (400 MHz, $CDCl_3$): *δ* = 7.56 – 7.36 (m, 4H), 6.66 (s, 1H), 3.83 (s, 2H), 3.57 – 3.52 (m, 2H), 2.89 (t, *J* = 7.1 Hz, 2H). **^{13}C NMR** (101 MHz, $CDCl_3$): *δ* = 165.89, 139.41, 132.29, 123.73, 41.25, 35.31, 29.06. **HRMS (ESI)**: *m/z* = 310.0049 calcd. for [$C_{11}H_{11}BrF_3NO$ + H]$^+$; found: 310.004.

Synthesis of 2-((3-(trifluoromethyl)phenethylcarbamoyl)methoxy)phenylphosphonic acid (**28**)

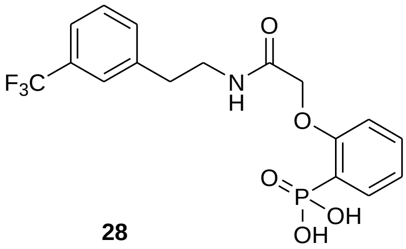

28

The phenylphosphonic acid derivative **28** was synthesized via general methods E and F. General method E employed phosphonate **SI-2** (101 mg, 0.5 mmol), potassium carbonate (0.14 g, 1.0 mmol) and **SI-21** (171 mg, 0.55 mmol) in acetone (12 mL). The crude intermediate was deprotected via general method F using TMSBr (0.33 mL, 2.5 mmol) in dry DCM (3.0 mL) and MeOH/H_2O (3:1, 3.0 mL). The crude product was purified by HPLC to obtain **28** (75 mg, 0.17 mmol, 37 % over two steps) as a white solid. **LC-MS (ESI)**: t_R = 6.77 min; *m/z* = 404.02 [M + H]$^+$. **^{1}H NMR** (400 MHz, DMSO): *δ* = 9.22 (s, 1H), 7.66 – 7.60 (m, 1H),

7.56 – 7.42 (m, 5H), 7.14 – 7.04 (m, 2H), 4.58 (s, 2H), 3.41 – 3.37 (m, 2H), 2.86 (t, J = 7.3 Hz, 2H). ^{13}**C NMR** (101 MHz, DMSO): δ = 167.88, 158.94, 140.78, 132.61, 129.21, 125.16, 123.05, 121.07, 113.26, 99.55, 67.67, 34.42. **HRMS (ESI)**: m/z = 404.0869 calcd. for $[C_{17}H_{17}F_3NO_5P + H]^+$; found: 404.0857.

Synthesis of N-(4-(trifluoromethyl)phenethyl)-2-bromoacetamide (**SI-22**)

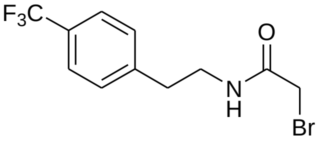

SI-22

The bromoacetamide **SI-22** was synthesized via general method D using 2-(4-trifluoromethyl)phenethylamine (0.80 mL, 5.00 mmol), triethylamine (0.76 mL, 5.50 mmol) and bromoacetyl bromide (0.44 mL, 5.00 mmol) in dry DCM (25 mL). The crude product was purified by HPLC to obtain **SI-22** (1.52 g, 4.91 mmol, 98 %) as a white solid. **LC-MS (ESI)**: t_R = 8.85 min; m/z = 310.08 $[M + H]^+$. ^{1}**H NMR** (400 MHz, $CDCl_3$): δ = 7.53 (d, J = 7.7 Hz, 2H), 7.31 (d, J = 7.7 Hz, 2H), 6.79 (s, 1H), 3.80 (s, 2H), 3.55 – 3.50 (m, 2H), 2.89 (t, J = 6.9 Hz, 2H). ^{13}**C NMR** (101 MHz, $CDCl_3$): δ = 165.90, 142.64, 129.17, 125.55, 122.89, 41.10, 35.26, 28.98. **HRMS (ESI)**: m/z = 310.0049 calcd. for $[C_{11}H_{11}BrF_3NO + H]^+$; found: 310.0041.

Synthesis of 2-((4-(trifluoromethyl)phenethylcarbamoyl)methoxy)phenylphosphonic acid (**29**)

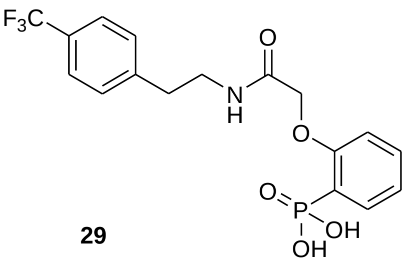

The phenylphosphonic acid derivative **29** was synthesized via general method E and general method F. General method E employed phosphonate **SI-2** (103 mg, 0.51 mmol), potassium carbonate (0.14 g, 1.02 mmol) and **SI-22** (174 mg, 0.56 mmol) in acetone (12 mL). The crude intermediate was deprotected via general method F using TMSBr (0.34 mL, 2.55 mmol) in dry DCM (3.0 mL) and MeOH/H_2O (3:1, 3.0 mL). The crude product was purified by HPLC to obtain **29** (47 mg, 0.12 mmol, 23 % over two steps) as a white solid. **LC-MS (ESI)**: t_R = 6.76 min; m/z = 404.05 $[M + H]^+$. ^{1}**H NMR** (400 MHz, DMSO): δ = 9.20 (s, 1H), 7.66 – 7.60 (m, 2H), 7.54 (d, J = 8.0 Hz, 2H), 7.51 – 7.48 (m, 2H), 7.40 (d, J = 8.0 Hz, 2H), 7.15 – 7.05 (m, 2H), 4.60 (s, 2H), 3.42 – 3.37 (m, 2H), 2.85 (t, J = 7.1 Hz, 2H). ^{13}**C NMR** (101 MHz, DMSO): δ = 167.91, 158.88, 144.29, 133.11, 129.45, 125.04, 121.34, 113.17, 67.60, 34.49. **HRMS (ESI)**: m/z = 404.0869 calcd. for $[C_{17}H_{17}F_3NO_5P + H]^+$; found: 404.086.

Synthesis of the derivatives 30 - 32

Synthesis of N-(2-hydroxyphenethyl)-2-bromoacetamide (**SI-23**)

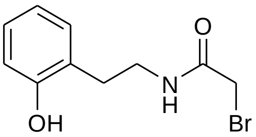

SI-23

The bromoacetamide **SI-23** was synthesized via general method D using 2-(2-methoxyphenyl)ethanamine (0.73 mL, 5.00 mmol), triethylamine (0.76 mL, 5.50 mmol) and bromoacetyl bromide (0.44 mL, 5.00 mmol) in dry DCM (25 mL). The crude intermediate was dissolved in dry DCM (25 mL), boron tribromide (0.95 mL, 10.00 mmol, 2.00eq) was added and the resulting solution was stirred for 2 h at room temperature. The mixture was poured into water, the product was extracted with DCM and the combined organic layers were dried over $MgSO_4$. The crude product was purified by HPLC to obtain **SI-23** (1.01 g, 3.93 mmol, 79 %) as a white solid. **LC-MS (ESI)**: t_R = 6.76 min; m/z = 257.97 $[M + H]^+$. ^{1}**H NMR** (400 MHz, DMSO): δ = 8.33 (s, 1H), 7.05 – 7.00 (m, 2H), 6.81 (d, J = 7.9 Hz, 1H), 6.71 (t, J = 7.2 Hz, 1H), 3.83 (s, 2H), 3.31 – 3.26 (m, 2H), 2.69 (t, J = 7.2 Hz, 2H). ^{13}**C NMR** (101 MHz, DMSO): δ = 165.90, 155.32, 130.26, 127.35, 125.21, 118.95, 114.95, 61.78, 38.57, 29.62. **HRMS (ESI)**: m/z = 258.0124 calcd. for $[C_{10}H_{12}BrNO_2 + H]^+$; found: 258.012.

Synthesis of dimethyl 2-((2-hydroxyphenethylcarbamoyl)methoxy) phenylphosphonate (**SI-24**)

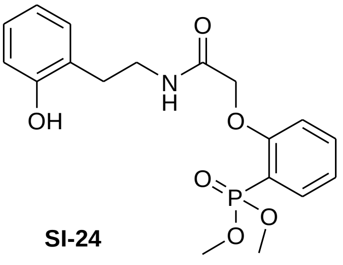

The phenylphosphonic acid derivative **SI-24** was synthesized via general method E using phosphonate **SI-2** (0.86 g, 4.27 mmol), potassium carbonate (1.18 g, 8.54 mmol) and **SI-23** (1.31 g, 4.70 mmol) in acetone (100 mL). The crude product was purified by HPLC to obtain **SI-24** (1.04 g, 2.73 mmol, 64 %) as a white solid. **LC-MS (ESI)**: t_R = 6.83 min; m/z = 380.08 $[M + H]^+$. ^{1}**H NMR** (400 MHz, $CDCl_3$): δ = 8.85 (s, 1H), 7.71 – 7.65 (m, 1H), 7.56 – 7.52 (m, 2H), 7.11 – 7.03 (m, 2H), 6.91 (t, J = 8.0 Hz, 1H), 6.85 (d, J = 7.9 Hz, 1H), 6.78 (t, J = 7.3 Hz, 1H), 4.65 (s, 2H), 3.85 (d, J = 11.4 Hz, 6H), 3.56 – 3.53 (m, 2H), 2.88 (t, J = 6.3 Hz, 2H). ^{13}**C NMR** (101 MHz, $CDCl_3$): δ = 169.30, 159.45, 154.82, 135.40, 134.12, 130.61, 128.08, 125.60, 122.18, 120.28, 115.98, 113.99, 112.60, 67.84, 53.59, 41.71, 29.33. **HRMS (ESI)**: m/z = 380.1258 calcd. for $[C_{18}H_{22}NO_6P + H]^+$; found: 380.1248.

Synthesis of 2-((2-(benzyloxy)phenethylcarbamoyl)methoxy)phenylphosphonic acid (**30**)

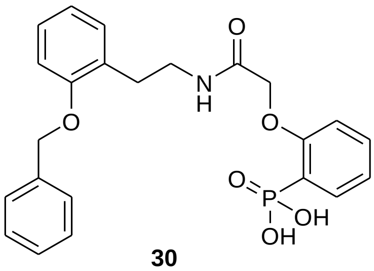

30

The phenylphosphonic acid derivative **30** was synthesized via general method E and general method F. General method E employed phosphonate **SI-24** (258 mg, 0.68 mmol), potassium carbonate (0.19 g, 1.36 mmol) and benzyl bromide (89 µL, 0.75 mmol) in acetone (17 mL). The crude intermediate was deprotected via general method F using TMSBr (0.45 mL, 3.40 mmol) in dry DCM (4.0 mL) and MeOH/H_2O (3:1, 4.0 mL). The crude product was purified by HPLC to obtain **30** (66 mg, 0.15 mmol, 22 % over two steps) as a white solid. **LC-MS (ESI)**: t_R = 7.57 min; *m/z* = 442.07 $[M + H]^+$.**^{1}H NMR** (400 MHz, DMSO): δ = 9.13 (s, 1H), 7.66 – 7.61 (m, 1H), 7.48 (d, *J* = 7.7 Hz, 2H), 7.40 (t, *J* = 7.5 Hz, 2H), 7.31 (t, *J* = 7.3 Hz, 1H), 7.23 - 7.06 (m, 3H), 7.09 - 6.97 (m, 2H), 6.80 (t, *J* = 7.3 Hz, 1H), 5.13 (s, 2H), 4.60 (s, 2H), 3.39 - 3.34 (m, 2H), 2.80 (t, *J* = 8.6 Hz, 2H). **^{13}C NMR** (101 MHz, DMSO): δ = 167.82, 158.93, 156.13, 137.40, 133.15, 132.59, 130.18, 128.51, 127.59, 127.50, 127.14, 123.16, 121.37, 121.04, 120.52, 113.26, 112.05, 69.06, 67.68, 38.55, 29.83. **HRMS (ESI)**: *m/z* = 442.1414 calcd. for $[C_{23}H_{24}NO_6P + H]^+$; found: 442.1405.

Synthesis of N-(3-hydroxyphenethyl)-2-bromoacetamide (**SI-25**)

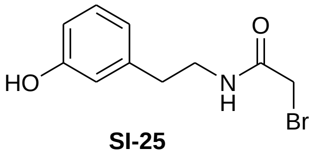

SI-25

The bromoacetamide **SI-25** was synthesized via general method D using 2-(3-methoxyphenyl)ethanamine (0.73 mL, 5.00 mmol), triethylamine (0.76 mL, 5.50 mmol) and bromoacetyl bromide (0.44 mL, 5.00 mmol) in dry DCM (25 mL). The crude intermediate was dissolved in dry DCM (25 mL), boron tribromide (0.95 mL, 10.00 mmol, 2.00eq) was added and the resulting solution was stirred for 2 h at room temperature. The mixture was poured into water, the product extracted with DCM and the combined organic layers were dried over $MgSO_4$. The crude product was purified by HPLC to obtain **SI-25** (1.10 g, 4.28 mmol, 86 %) as a white solid. **LC-MS (ESI)**: t_R = 6.31 min; *m/z* = 258.02 $[M + H]^+$. **^{1}H NMR** (400 MHz, $CDCl_3$): δ = 7.10 (t, *J* = 7.7 Hz, 1H), 6.83 – 6.78 (m, 1H), 6.72 – 6.64 (m, 2H), 6.37 (s, 1H), 3.77 (s, 2H), 3.48 – 3.43 (m, 2H), 2.71 (t, *J* = 6.9 Hz, 2H). **^{13}C NMR** (101 MHz, $CDCl_3$): δ = 166.76, 156.63, 139.88, 129.97, 120.66, 115.82, 114.04, 41.48, 35.12, 28.97. **HRMS (ESI)**: *m/z* = 258.0124 calcd. for $[C_{10}H_{12}BrNO_2 + H]^+$; found: 258.0121.

Synthesis of dimethyl 2-((3-hydroxyphenethylcarbamoyl)methoxy) phenylphosphonate (**SI-26**)

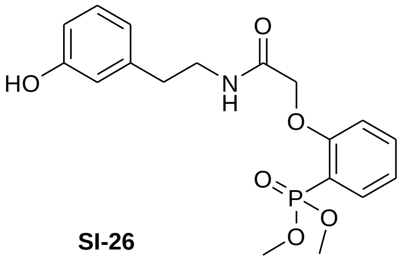

SI-26

The phenylphosphonic acid derivative **SI-26** was synthesized via general method E using phosphonate **SI-2** (0.93 g, 4.58 mmol), potassium carbonate (1.27 g, 9.16 mmol) and **SI-25** (1.40 g, 5.04 mmol) in acetone (100 mL). The crude product was purified by HPLC to obtain **SI-26** (0.86 g, 2.27 mmol, 50 %) as a white solid. **LC-MS (ESI)**: t_R = 7.21 min; *m/z* = 380.09 $[M + H]^+$. **^{1}H NMR** (400 MHz, $CDCl_3$): δ = 8.30 (s, 1H), 7.69 – 7.64 (m, 1H), 7.53 (t, *J* = 7.8 Hz, 1H), 7.10 – 7.04 (m, 2H), 6.90 (t, *J* = 7.5 Hz, 1H), 6.80 (s, 1H), 6.71 – 6.55 (m, 3H), 6.50 (s, 1H), 4.59 (s, 2H), 3.72 (d, *J* = 11.4 Hz, 6H), 3.59 – 3.55 (m, 2H), 2.81 (t, *J* = 6.9 Hz, 2H). **^{13}C NMR** (101 MHz, $CDCl_3$): δ = 168.40, 159.25, 157.02, 140.39, 135.27, 134.20, 129.55, 122.04, 120.25, 115.80, 113.92, 113.61, 112.64, 67.51, 53.22, 40.60, 35.29. **HRMS (ESI)**: *m/z* = 380.1258 calcd. for $[C_{18}H_{22}NO_6P + H]^+$; found: 380.1249.

Synthesis of 2-((3-(benzyloxy)phenethylcarbamoyl)methoxy)phenylphosphonic acid (**31**)

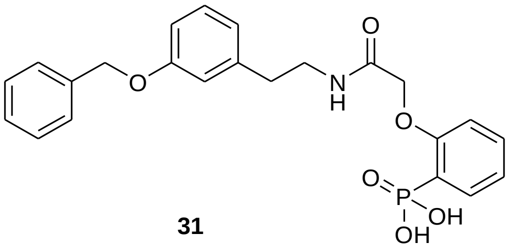

31

The phenylphosphonic acid derivative **31** was synthesized via general method E and general method F. General method E employed phosphonate **SI-26** (171 mg, 0.45 mmol), potassium carbonate (0.12 g, 0.90 mmol) and benzyl bromide (59 µL, 0.50 mmol) in acetone (11 mL). The crude intermediate was deprotected via general method F using TMSBr (0.30 mL, 2.25 mmol) in dry DCM (3.0 mL) and MeOH/H_2O (3:1, 3.0 mL). The crude product was purified by HPLC to obtain **31** (50 mg, 0.15 mmol, 25 % over two steps) as a white solid. **LC-MS (ESI)**: t_R = 7.57 min; *m/z* = 442.01 $[M + H]^+$. **^{1}H NMR** (400 MHz, DMSO): δ = 9.18 (s, 1H), 7.65 – 7.59 (m, 1H), 7.49 (t, *J* = 7.6 Hz, 1H), 7.51 - 7.30 (m, 5H), 7.17 – 7.11 (m, 1H), 7.07 – 7.03 (m, 1H), 6.83 -6-76 (m, 2H), 5.04 (s, 2H), 4.60 (s, 2H), 3.37 - 3.32 (m, 2H), 2.73 (t, *J* = 7.5 Hz, 2H). **^{13}C NMR** (101 MHz, DMSO): δ = 167.81, 158.91, 158.39, 140.94, 137.18, 133.14, 132.55, 127.76, 123.06, 121.06, 114.92, 113.26, 112.59, 69.05, 67.71, 34.94. **HRMS (ESI)**: *m/z* = 442.1414 calcd. for $[C_{23}H_{24}NO_6P + H]^+$; found: 442.1394.

Synthesis of N-(4-hydroxyphenethyl)-2-bromoacetamide (**SI-27**)

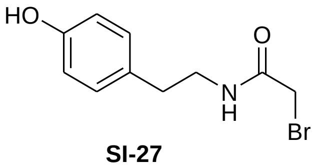

SI-27

The bromoacetamide **SI-27** was synthesized via general method D using tyramine (0.69 g, 5.00 mmol), triethylamine (0.76 mL, 5.50 mmol) and bromoacetyl bromide (0.44 mL, 5.00 mmol) in dry DCM (25 mL) and additional dry methanol (2.5 mL). The crude product was purified by HPLC to obtain **SI-27** (0.62 g, 2.41 mmol, 48 %) as a white solid. **LC-MS (ESI)**: t_R = 6.11 min; *m/z* = 258.07 $[M + H]^+$. **^{1}H NMR** (400 MHz, MeOD): δ = 7.05 (d, *J* = 8.5 Hz, 2H), 6.75 (d, *J* = 8.5 Hz, 2H), 3.81 (s, 2H), 3.40 (t, *J* = 7.3 Hz, 2H), 2.73 (t, *J* = 7.3 Hz, 2H). **^{13}C NMR** (101 MHz, MeOD): δ = 169.32, 156.94, 130.74, 116.24, 42.81, 35.34, 28.78. **HRMS (ESI)**: *m/z* = 258.0124 calcd. for $[C_{10}H_{12}BrNO_2 + H]^+$; found: 258.0122.

Synthesis of dimethyl 2-((4-hydroxyphenethylcarbamoyl)methoxy) phenylphosphonate (**SI-28**)

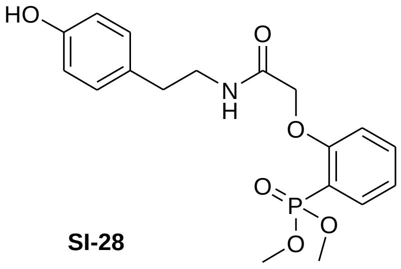

SI-28

The phenylphosphonic acid derivative **SI-28** was synthesized via general method E using phosphonate **SI-2** (185 mg, 0.72 mmol), potassium carbonate (0.2 g, 1.44 mmol) and **SI-27** (185 mg, 0.79 mmol) in acetone (20 mL). The crude product was purified by HPLC to obtain **SI-28** (186 mg, 0.49 mmol, 69 %) as a white solid. **LC-MS (ESI)**: t_R = 6.61 min; *m/z* = 380.00 $[M + H]^+$. **^{1}H NMR** (400 MHz, $CDCl_3$): δ = 8.43 (s, 1H), 7.72 – 7.66 (m, 1H), 7.59 – 7.49 (m, 2H), 7.12 (s, 1H), 6.98 – 6.92 (m, 3H), 6.74 (d, *J* = 7.1 Hz, 2H), 4.65 (s, 2H), 3.77 (d, *J* = 7.1 Hz, 6H), 3.57 – 3.52 (m, 2H), 2.78 (t, *J* = 7.2 Hz, 2H). **^{13}C NMR** (101 MHz, $CDCl_3$): δ = 169.08, 159.23, 155.01, 135.62, 134.42, 130.05, 129.90, 122.23, 115.53, 113.63, 112.76, 67.30, 53.47, 41.31, 34.62. **HRMS (ESI)**: *m/z* = 380.1258 calcd. for $[C_{18}H_{22}NO_6P + H]^+$; found: 380.1248.

Synthesis of 2-((4-(benzyloxy)phenethylcarbamoyl)methoxy)phenylphosphonic acid (**32**)

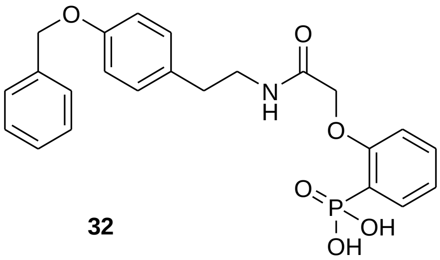

32

The phenylphosphonic acid derivative **32** was synthesized via general methods E and F. General method E employed phosphonate **SI-28** (243 mg, 0.64 mmol), potassium carbonate (0.18 g, 1.28 mmol) and benzyl bromide (83 µL, 0.70 mmol) in acetone (16 mL). The crude intermediate was deprotected via general method F using TMSBr (0.42 mL, 3.20 mmol) in dry DCM (4.0 mL) and $MeOH/H_2O$ (3:1, 4.0 mL). The crude product was purified by HPLC to obtain **32** (46 mg, 0.10 mmol, 16 % over two steps) as a white solid. **LC-MS (ESI)**: t_R = 6.76 min; *m/z* = 442.13 $[M + H]^+$. **^{1}H NMR** (400 MHz, DMSO): δ = 9.13 (s, 1H), 7.66 – 7.60 (m, 1H), 7.49 (t, *J* = 7.6 Hz, 1H), 7.44 – 7.36 (m, 4H), 7.34 – 7.30 (m, 1H), 7.15 – 7.07 (m, 4H), 6.87 (d, *J* = 8.5 Hz, 2H), 5.04 (s, 2H), 4.60 (s, 2H), 3.32 – 3.27 (m, 2H), 2.67 (t, *J* = 7.5 Hz, 2H). **^{13}C NMR** (101 MHz, DMSO): δ = 167.92, 158.96, 156.79, 137.31, 133.24, 132.64, 131.52, 129.64, 128.50, 127.85, 127.70, 123.09, 121.14, 114.68, 113.29, 69.21, 67.68, 40.37, 34.10. **HRMS (ESI)**: *m/z* = 442.1414 calcd. for $[C_{23}H_{24}NO_6P + H]^+$; found: 442.1405.

Synthesis of the derivatives 33 – 35

Synthesis of N-(4-chlorophenethyl)-2-bromoacetamide (**SI-29**)

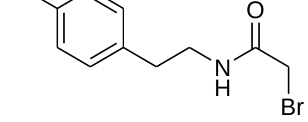

SI-29

The bromoacetamide **SI-29** was synthesized via general method D using 2-(4-chlorophenyl)ethanamine (0.28 mL, 2.00 mmol), triethylamine (0.31 mL, 2.20 mmol) and bromoacetyl bromide (0.17 mL, 2.00 mmol) in dry DCM (10 mL). The crude product was purified by HPLC to obtain **SI-29** (0.44 g, 1.60 mmol, 80 %) as a white solid. **LC-MS (ESI)**: t_R = 8.54 min; *m/z* = 277.97 $[M + H]^+$. **^{1}H NMR** (400 MHz, $CDCl_3$): δ = 7.19 (d, *J* = 8.2 Hz, 2H), 7.06 (d, *J* = 7.1 Hz, 2H), 6.51 (s, 1H), 3.75 (s, 2H), 3.46 – 3.41 (m, 2H), 2.73 (t, *J* = 6.8 Hz, 2H). **^{13}C NMR** (101 MHz, $CDCl_3$): δ = 165.57, 136.90, 132.60, 130.19, 128.89, 41.27, 34.86, 29.23. **HRMS (ESI)**: *m/z* = 275.9785 calcd. for $[C_{10}H_{11}BrClNO + H]^+$; found: 275.9778.

Synthesis of 2-((4-chlorophenethylcarbamoyl)methoxy)phenylphosphonic acid (**33**)

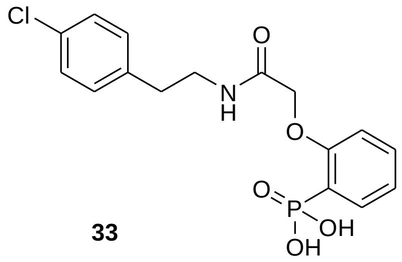

33

The phenylphosphonic acid derivative **33** was synthesized via general method E and general method F. General method E employed phosphonate **SI-2** (34 mg, 0.17 mmol), potassium carbonate (0.05 g, 0.34 mmol) and **SI-29** (53 mg, 0.19 mmol) in acetone (4 mL). The crude intermediate was deprotected via general method F using TMSBr (0.11 mL, 0.85 mmol) in dry DCM (1.0 mL) and MeOH/H_2O (3:1, 1.0 mL). The crude product was purified by HPLC to obtain **33** (13 mg, 0.04 mmol, 23 % over two steps) as a white solid. **LC-MS (ESI)**: t_R = 5.70 min; *m/z* = 370.16 $[M + H]^+$. **^{1}H NMR** (400 MHz, MeOD): *δ* = 7.77 – 7.71 (m, 1H), 7.50 (t, *J* = 7.8 Hz, 1H), 7.20 – 7.13 (m, 4H), 7.10 – 7.06 (m 1H), 7.01 – 6.98 (m, 1H), 4.59 (s, 2H), 3.47 (t, *J* = 7.4 Hz, 2H), 2.79 (t, *J* = 7.5 Hz, 2H). **^{13}C NMR** (101 MHz, MeOD): δ = 170.90, 160.40, 139.08, 133.08, 131.45, 129.54, 129.44, 122.59, 113.63, 68.95, 41.49, 35.63. **HRMS (ESI)**: *m/z* = 370.0606 calcd. for $[C_{16}H_{17}ClNO_5P + H]^+$; found: 370.0602.

Synthesis of 2-((4-hydroxyphenethylcarbamoyl)methoxy)phenylphosphonic acid (**34**)

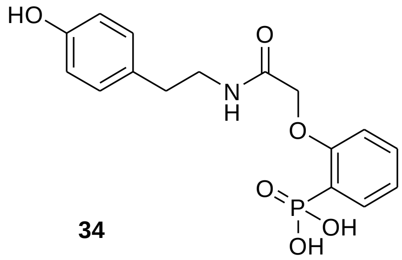

34

The phenylphosphonic acid derivative **34** was synthesized via general methods E and F. General method E employed phosphonate **SI-2** (85 mg, 0.42 mmol), potassium carbonate (0.12 g, 0.84 mmol) and **SI-27** (118 mg, 0.46 mmol) in acetone (11 mL). The crude intermediate was deprotected via general method F using TMSBr (0.28 mL, 2.10 mmol) in dry DCM (2.5 mL) and MeOH/H_2O (3:1, 2.4 mL). The crude product was purified by HPLC to obtain **33** (45 mg, 0.13 mmol, 30 % over two steps) as a white solid. **LC-MS (ESI)**: t_R = 4.20 min; *m/z* = 352.01 $[M + H]^+$. **^{1}H NMR** (400 MHz, MeOD): *δ* = 7.66 – 7.60 (m, 1H), 7.39 (t, *J* = 7.8 Hz, 1H), 6.99 – 6.95 (m, 1H), 7.34 – 7.30 (m, 1H), 6.90 – 6.86 (m, 3H), 6.54 (d, *J* = 8.4 Hz, 2H), 4.48 (s, 2H), 3.30 (t, *J* = 7.6 Hz, 2H), 2.59 (t, *J* = 7.6 Hz, 2H). **^{13}C NMR** (101 MHz, MeOD): *δ* = 170.78, 160.45, 156.66, 135.09, 134.33, 131.00, 130.71, 122.76, 120.72, 116.20, 113.62, 68.42, 42.12, 35.61. **HRMS (ESI)**: *m/z* = 352.0945 calcd. for $[C_{16}H_{18}NO_6P$: 351.09 + $H]^+$; found: 352.0934.

Synthesis of N-(4-bromophenethyl)-2-bromoacetamide (**SI-30**)

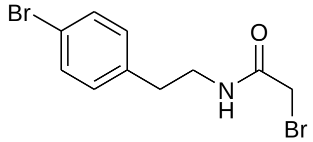

SI-30

The bromoacetamide **SI-30** was synthesized via general method D using 2-(4-bromophenyl)ethanamine (0.31 mL, 2.00 mmol), triethylamine (0.31 mL, 2.20 mmol) and bromoacetyl bromide (0.17 mL, 2.00 mmol) in dry DCM (10 mL). The crude product was purified by HPLC to obtain **SI-30** (0.57 g, 1.76 mmol, 88 %) as a white solid. **LC-MS (ESI)**: t_R = 8.72 min; *m/z* = 321.87 $[M + H]^+$. **^{1}H NMR** (400 MHz, $CDCl_3$): *δ* = 7.41 (d, *J* = 8.0 Hz, 2H), 7.08 (d, *J* = 7.1 Hz, 2H), 6.60 (s, 1H), 3.82 (s, 2H), 3.52 – 3.48 (m, 2H), 2.79 (t, *J* = 6.8 Hz, 2H). **^{13}C NMR** (101 MHz, $CDCl_3$): *δ* = 165.60, 137.41, 131.85, 130.58, 120.65, 41.21, 34.93, 29.21. **HRMS (ESI)**: *m/z* = 319.928 calcd. for $[C_{10}H_{11}Br_2NO + H]^+$; found: 319.9271.

Synthesis of 2-((4-bromophenethylcarbamoyl)methoxy)phenylphosphonic acid (**35**)

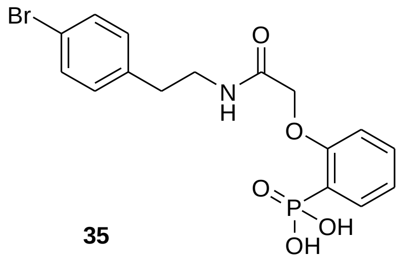

35

The phenylphosphonic acid derivative **35** was synthesized via general method E and general method F. General method E employed phosphonate **SI-2** (26 mg, 0.13 mmol), potassium carbonate (0.05 g, 0.26 mmol) and **SI-30** (45 mg, 0.14 mmol) in acetone (3 mL). The crude intermediate was deprotected via general method F using TMSBr (90 µL, 0.65 mmol) in dry DCM (1.0 mL) and MeOH/H_2O (3:1, 1.0 mL). The crude product was purified by HPLC to obtain **35** (8 mg, 0.02 mmol, 15 % over two steps) as a white solid. **LC-MS (ESI)**: t_R = 5.83 min; *m/z* = 414.15 $[M + H]^+$. **^{1}H NMR** (400 MHz, MeOD): *δ* = 8.05 – 7.99 (m, 1H), 7.77 (t, *J* = 7.8 Hz, 1H), 7.61 – 7.59 (m, 1H), 7.40 – 7.36 (m, 3H), 7.10 – 7.06 (m 1H), 7.29 – 7.25 (m, 1H), 4.87 (s, 2H), 3.75 (t, *J* = 7.3 Hz, 2H), 3.06 (t, *J* = 7.5 Hz, 2H). **^{13}C NMR** (101 MHz, MeOD): δ = 170.49, 161.58, 144.96, 139.32, 137.24, 135.07, 132.67, 132.06, 121.22, 115.03, 82.27, 49.64, 29.52, 27.21. **HRMS (ESI)**: *m/z* = 414.0100 calcd. for $[C_{16}H_{17}BrNO_5P + H]^+$; found: 414.0100.

Synthesis of the derivatives 36 - 37

Synthesis of 2-bromo-N-((S)-2-hydroxy-2-phenylethyl)acetamide (**SI-31**)

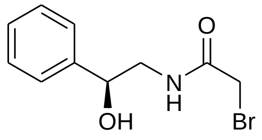

SI-31

The bromoacetamide **SI-31** was synthesized via general method D using (*S*)-2-amino-1-phenylethanol (0.69 g, 5.00 mmol), triethylamine (0.76 mL, 5.50 mmol) and bromoacetyl bromide (0.44 mL, 5.00 mmol) in dry DCM (25 mL). The crude product was purified by HPLC to obtain **SI-31** (0.59 g, 2.28 mmol, 46 %) as a white solid. **LC-MS (ESI)**: t_R = 6.20 min; *m/z* = 257.68 [M + H]$^+$, 240.08 [M-H_2O]. **^{1}H NMR** (400 MHz, $CDCl_3$): *δ* = 7.35 – 7.30 (m, 5H), 7.03 (s, 1H), 4.83 (d,*J* = 4.9 Hz, 1H), 3.84 (s, 2H), 3.70 – 3.66 (m, 1H), 3.39 – 3.32 (m, 1H). **^{13}C NMR** (101 MHz, $CDCl_3$): *δ* = 166.93, 141.27, 128.77, 128.29, 125.93, 72.99, 47.64, 29.02. **HRMS (ESI)**: *m/z* = 258.0124 calcd. for [$C_{10}H_{12}BrNO_2$ + H]$^+$; found: 258.0119.

Synthesis of 2-(((S)-2-hydroxy-2-phenylethylcarbamoyl)methoxy)phenylphosphonic acid (**36**)

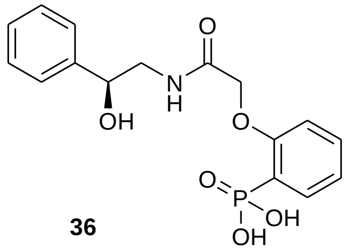

36

The phenylphosphonic acid derivative **36** was synthesized via general method E and general method F. General method E employed phosphonate **SI-2** (63 mg, 0.31 mmol), potassium carbonate (0.09 g, 0.62 mmol) and **SI-31** (88 mg, 0.34 mmol) in acetone (8 mL). The crude intermediate was deprotected via general method F using TMSBr (0.20 mL, 1.55 mmol) in dry DCM (2.0 mL) and MeOH/H_2O (3:1, 2.0 mL). The crude product was purified by HPLC to obtain **36** (36 mg, 0.10 mmol, 33 % over two steps) as a white solid. **LC-MS (ESI)**: t_R = 4.49 min; *m/z* = 351.89 [M + H]$^+$. **^{1}H NMR** (400 MHz, MeOD): *δ* = 7.73 – 7.67 (m, 1H), 7.48 (t, *J* = 7.9 Hz, 1H), 7.28 – 7.16 (m, 5H), 7.07 – 7.02 (m 1H), 7.00 – 6.96 (m, 1H), 4.78 – 4.75 (m, 1H), 4.59 (s, 2H), 3.52 – 3.27 (m, 2H). **^{13}C NMR** (101 MHz, MeOD): δ = 170.90, 160.40, 139.08, 133.08, 131.45, 129.54, 129.44, 122.59, 113.63, 68.95, 41.49, 35.63. **HRMS (ESI)**: *m/z* = 352.0945 calcd. for [$C_{16}H_{18}NO_6P$ + H]$^+$; found: 352.0934.

Synthesis of 2-bromo-N-((R)-2-hydroxy-2-phenylethyl)acetamide (**SI-32**)

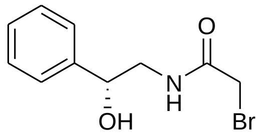

SI-32

The bromoacetamide **SI-32** was synthesized via general method D using (*R*)-2-amino-1-phenylethanol (0.69 g, 5.00 mmol), triethylamine (0.76 mL, 5.50 mmol) and bromoacetyl bromide (0.44 mL, 5.00 mmol) in dry DCM (25 mL). The crude product was purified by HPLC to obtain **SI-32** (0.71 g, 3.50 mmol, 70 %) as a white solid. **LC-MS (ESI)**: t_R = 6.20 min; *m/z* = 257.67 [M + H]$^+$, 240.06 [M - H_2O]. calcd. for $C_{10}H_{12}BrNO_2$: 257.01. **^{1}H NMR** (400 MHz, $CDCl_3$): *δ* = 7.30 – 7.23 (m, 5H), 7.03 (s, 1H), 4.78 (d,*J* = 7.7 Hz, 1H), 3.79 (s, 2H), 3.65 – 3.60 (m, 1H), 3.34 – 3.28 (m, 1H). **^{13}C NMR** (101 MHz, $CDCl_3$): *δ* = 166.89, 141.32, 128.72, 128.22, 125.92, 72.89, 47.62, 29.02. **HRMS (ESI)**: *m/z* = 258.0124 calcd. for [$C_{10}H_{12}BrNO_2$ + H]$^+$; found: 258.0118.

Synthesis of 2-(((R)-2-hydroxy-2-phenylethylcarbamoyl)methoxy)phenylphosphonic acid (**37**)

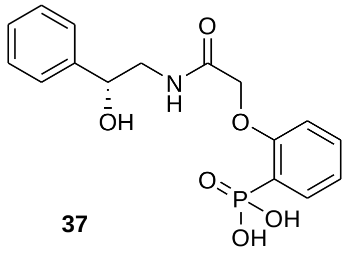

37

The phenylphosphonic acid derivative **37** was synthesized via general method E and general method F. General method E employed phosphonate **SI-2** (53 mg, 0.26 mmol), potassium carbonate (0.07 g, 0.52 mmol) and **SI-32** (75 mg, 0.29 mmol) in acetone (7 mL). The crude intermediate was deprotected via general method F using TMSBr (0.17 mL, 1.30 mmol) in dry DCM (1.5 mL) and MeOH/H_2O (3:1, 1.5 mL). The crude product was purified by HPLC to obtain **37** (22 mg, 0.06 mmol, 24 % over two steps) as a white solid. **LC-MS (ESI)**: t_R = 4.50 min; *m/z* = 351.89 [M + H]$^+$. **^{1}H NMR** (400 MHz, MeOD): *δ* = 7.76 – 7.70 (m, 1H), 7.51 (t, *J* = 7.6 Hz, 1H), 7.35 – 7.19 (m, 5H), 7.10 – 7.05 (m 1H), 7.03 – 6.99 (m, 1H), 4.80 – 4.77 (m, 1H), 4.62 (s, 2H), 3.53 – 3.37 (m, 2H). **^{13}C NMR** (101 MHz, MeOD): δ = 171.47, 160.87, 144.38, 135.66, 134.53, 129.75, 128.90, 127.87, 123.39, 120.84, 114.09, 73.82, 68.86, 48.31. **HRMS (ESI)**: *m/z* = 352.0945 calcd. for [$C_{16}H_{18}NO_6P$ + H]$^+$; found: 352.0933.

Synthesis of the derivatives 38 - 39

Synthesis of 1-bromo-5-phenylpentan-2-one (**SI-33**)

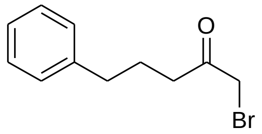

SI-33

Generation of the Grignard reagent: Magnesium (0.58 g, 24.00 mmol, 1.2 eq) was suspended in dry diethyl ether (10 mL) and 1,2-dibromoethane (4 drops) was added. 1-bromo-3-phenylpropane (3.04 mL, 20.00 mmol, 1.0 eq) was added dropwise. The reaction mixture was refluxed for 2 h and then cooled down to room temperature. Bromoacetylchloride (2.00 mL, 24.00 mmol, 1.2 eq) was dissolved in dry diethyl ether (8 mL) and cooled to 0 °C. The Grignard reagent was added dropwise. The

resulting mixture was stirred for 16 h, allowing the mixture to slowly reach room temperature. The reaction mixture was quenched with saturated NH_4Cl solution (5 mL). The resulting mixture was extracted with diethyl ether, the combined organic layers were dried over $MgSO_4$ and removed under reduced pressure. The crude product was purified by column chromatography (cyclohexane/ethyl acetate 9:1) to obtain **SI-33** (390 mg, 1.60 mmol, 8 %) as a yellow oil. **TLC** (cyclohexane:ethyl acetate, 9:1 v/v): R_f = 0.3. **^{1}H NMR** (400 MHz, $CDCl_3$): δ = 7.32 – 7.26 (m, 2H), 7.23 – 7.17 (m, 3H), 3.88 (s, 2H), 3.39 (t, *J* = 6.3 Hz, 2H), 2.78 (t, *J* = 7.3 Hz, 2H), 2.20 – 2.12 (m, 2H). **^{13}C NMR** (101 MHz, $CDCl_3$): δ = 201.60, 167.40, 141.17, 128.82, 126.36, 65.91, 32.02, 30.88, 26.21.

Synthesis of 2-(2-oxo-5-phenylpentyloxy)phenylphosphonic acid (**38**)

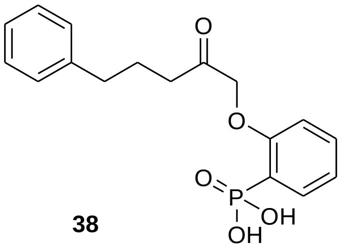

38

The phenylphosphonic acid derivative **38** was synthesized via general method E and general method F. General method E employed phosphonate **SI-2** (30 mg, 0.15 mmol), potassium carbonate (0.04 g, 0.30 mmol) and **SI-33** (41 mg, 0.17 mmol) in acetone (4 mL). The crude intermediate was deprotected via general method F using TMSBr (99 µL, 0.75 mmol) in dry DCM (1 mL) and MeOH/H_2O (3:1, 1 mL). The crude product was purified by HPLC to obtain **38** (15 mg, 0.05 mmol, 32 % over two steps) as a white solid. **LC-MS (ESI)**: t_R = 7.19 min; *m/z* = 334.95 $[M + H]^+$. **^{1}H NMR** (400 MHz, DMSO): δ = 10.77 (s, 1H), 7.71 – 7.05 (m, 1H), 7.43 (t, *J* = 7.5 Hz, 1H), 7.29 – 7.23 (m, 2H), 7.20 – 7.15 (m, 3H), 7.03 - 6.90 (m, 1H), 6.89 - 6.86 (m, 1H), 4.75 (s, 2H), 2.67 (t, *J* = 7.3 Hz, 2H), 2.57 – 2.53 (m, 2H), 1.83 - 1.76 (m, 2H). **^{13}C NMR** (101 MHz, DMSO): δ = 207.56, 158.97, 141.77, 133.33, 132.81, 128.35, 125.80, 121.64, 120.38, 112.64, 72.71, 37.79, 34.59, 24.69. **HRMS (ESI)**: *m/z* = 335.1043 calcd. for $[C_{17}H_{19}NO_5P + H]^+$; found: 335.1035.

Synthesis of 2-bromo-N-methyl-N-phenethylacetamide (**SI-34**)

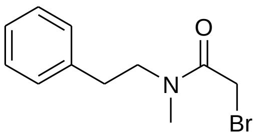

SI-34

The bromoacetamide **SI-34** was synthesized via general method D using *N*-methylphenethylamine (0.73 mL, 5.00 mmol), triethylamine (0.76 mL, 5.50 mmol) and bromoacetyl bromide (0.44 mL, 5.00 mmol) in dry DCM (25 mL). The crude product was purified by HPLC to obtain **SI-34** (1.18 g, 4.62 mmol, 92 %) as a white solid. **LC-MS (ESI)**: t_R = 8.38 min; *m/z* = 256.14 $[M + H]^+$. **^{1}H NMR** (400 MHz, $CDCl_3$): δ = 7.24 - 7.08 (m, 5H), 3.77 (s, 1H), 3.54 – 3.50 (m, 2H), 3.39 (s, 1H), 2.92 – 2.77 (m, 5H). **^{13}C NMR** (101 MHz, $CDCl_3$): δ = 166.73, 138.76, 128.95, 126.57, 50.72, 37.10, 34.69, 26.49. **HRMS (ESI)**: *m/z* = 256.0332 calcd. for $[C_{11}H_{14}BrNO + H]^+$; found: 256.0326.

Synthesis of 2-((N-methyl-N-phenethylcarbamoyl)methoxy)phenylphosphonic acid (**39**)

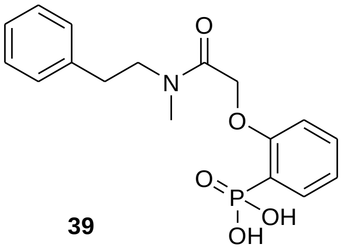

39

The phenylphosphonic acid derivative **39** was synthesized via general method E and general method F. General method E employed phosphonate **SI-2** (133 mg, 0.47 mmol), potassium carbonate (0.13 g, 0.94 mmol) and **SI-34** (174 mg, 0.52 mmol) in acetone (12 mL). The crude intermediate was deprotected via general method F using TMSBr (0.31 mL, 2.35 mmol) in dry DCM (3.0 mL) and MeOH/H_2O (3:1, 3.0 mL). The crude product was purified by HPLC to obtain **39** (50 mg, 0.14 mmol, 30 % over two steps) as a white solid. **LC-MS (ESI)**: t_R = 6.10 min; *m/z* = 350.13 $[M + H]^+$. **^{1}H NMR** (400 MHz, DMSO): δ = 7.64 – 7.58 (m, 1H), 7.50 – 7.40 (m, 1H), 7.31 – 7.18 (m, 5H), 7.09 – 7.00 (m, 2H), 4.74 (s, 2H), 3.57 – 3.50 (m, 2H), 2.91 (s, 3H), 2.79 – 2.74 (m, 2H). **^{13}C NMR** (101 MHz, DMSO): δ = 168.96, 159.12, 138.56, 132.94, 129.15, 128.72, 126.30, 123.39, 121.63, 120.98, 113.65, 66.28, 49.32, 34.11, 33.32. **HRMS (ESI)**: *m/z* = 350.1152 calcd. for $[C_{17}H_{20}NO_5P + H]^+$; found: 350.1146.

Supplementary References

1. Thiel, P. *et al.* Virtual screening and experimental validation reveal novel small-molecule inhibitors of 14-3-3 protein-protein interactions. *Chemical Communications* **49**, 8468–8470 (2013).

2. Berthold, M. R. *et al.* KNIME: The konstanz information miner. *4th International Industrial Simulation Conference 2006, ISC 2006* 58–61 (2006) doi:10.1145/1656274.1656280.

3. Mazanetz, M. P., Marmon, R. J., Reisser, C. B. T. & Morao, I. Drug discovery applications for KNIME: an open source data mining platform. *Current topics in medicinal chemistry* **12**, 1965–79 (2012).

4. Keiser, M. J. *et al.* Predicting new molecular targets for known drugs. *Nature* **462**, 175–181 (2009).

5. RDKit, Open-Source Cheminformatics. http://www.rdkit.org.

6. Sherman, W., Day, T., Jacobson, M. P., Friesner, R. A. & Farid, R. Novel procedure for modeling ligand/receptor induced fit effects. *Journal of Medicinal Chemistry* **49**, 534–553 (2006).

7. Sherman, W., Beard, H. S. & Farid, R. Use of an induced fit receptor structure in virtual screening. *Chemical Biology and Drug Design* **67**, 83–84 (2006).

8. Sherman, W., Beard, H. S. & Farid, R. Use of an induced fit receptor structure in virtual screening. *Chemical Biology and Drug Design* **67**, 83–84 (2006).

9. Yilmaz, E. *et al.* Mono- and Bivalent 14-3-3 Inhibitors for Characterizing Supramolecular “Lysine Wrapping” of Oligoethylene Glycol (OEG) Moieties in Proteins. *Chemistry - A European Journal* **24**, 13807–13814 (2018).
